# Synthesis of Highly Reactive Ketenimines via Photochemical Rearrangement of Isoxazoles

**DOI:** 10.1021/acs.orglett.3c02556

**Published:** 2023-08-24

**Authors:** Cormac Bracken, Marcus Baumann

**Affiliations:** School of Chemistry, University College Dublin, Science Centre South, Dublin 4, Ireland

## Abstract

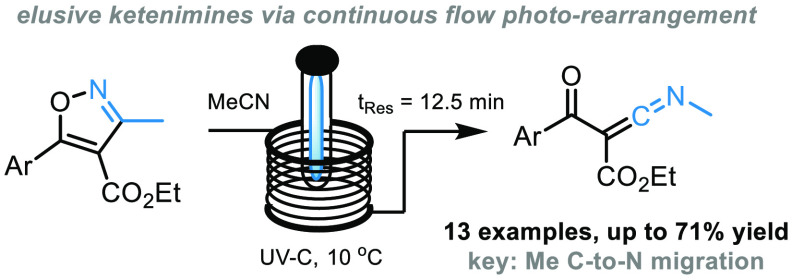

Ketenimines are highly electrophilic species with multiple applications as building blocks in organic synthesis; however, the effective preparation of these versatile entities remains a synthetic challenge. Here we report a continuous photochemical process that generates ketenimines via skeletal rearrangement of trisubstituted isoxazoles. The resulting flow process is noteworthy, as it provides for the first time a straightforward entry into these ketenimines that were previously only observed spectroscopically. The value of this methodology toward heterocyclic transposition reactions is demonstrated by converting isoxazoles via isolable ketenimines into pharmaceutically relevant pyrazoles.

Isoxazoles are a class of privileged heterocyclic scaffolds with a rich history in photochemistry. Specifically, the photoisomerization of isoxazoles has been studied extensively identifying oxazoles as the most common products.^1^ This process requires UV light (200–330 nm) for the direct irradiation of the substrate and proceeds via homolysis of the O–N bond to form an acyl azirine as a key intermediate. Although many decades have passed since the initial synthetic report by Ullman and Singh (Scheme 1A),^2^ renewed interest in this intriguing and highly atom-efficient process has arisen over the last years. Recent synthetic studies by Opatz and co-workers^3^ for instance exploited the intermediacy of photochemically generated azirines by their intermolecular conversion to densely functionalized pyrroles, whereas Zhang and co-workers^4^ demonstrated their intramolecular trapping via phenols to afford tricyclic aziridines (Scheme 1B). Spectroscopic studies using 3,5-dimethylisoxazole as substrate additionally provided insights into the formation of nitrile ylide intermediates as reported by Nunes and Reva.^5^ These species were trapped exploiting low temperature argon matrix experiments, which also reported scarce ketenimine intermediates.

**Scheme 1:**
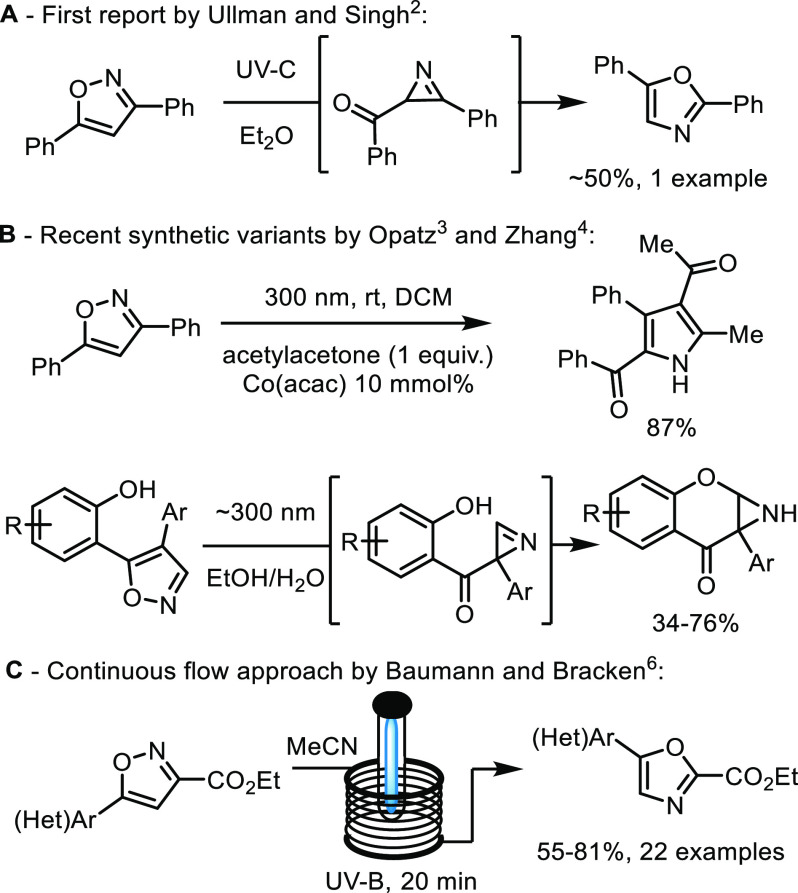
Studies of Isoxazole Photoisomerization Reactions

We recently demonstrated a robust synthetic approach to convert isoxazoles into their oxazole counterparts exploiting continuous flow reactor technology in combination with a medium-pressure Hg-lamp and a low-pass filter (Scheme 1C).^6^ This scalable approach facilitated the accessibility of a variety of druglike oxazoles that were obtained in short residence times (ca. 20 min) and high yields. Importantly this study provided for the first time a practical means to generate gram quantities of oxazole products from isoxazole precursors, thus allowing further exploitations of this neglected transformation.

Encouraged by our earlier studies, we wished to investigate whether the superb control imparted by microreactor technology^7^ can also be exploited to generate the elusive ketenimine intermediate postulated for variants of this isoxazole photoisomerization process.^5^ Not only would this underline the synthetic value of miniaturized flow setups, but moreover, the in situ generation of such highly reactive species would enable their telescoped transformation into various downstream products of interest. Therefore, a set of trisubstituted isoxazoles was identified and prepared via literature-known methods (see SI for details).^8^ Specifically, isoxazoles functionalized with an aryl group in the 5-position, an ester in the 4-position, and a methyl group in the 3-position were chosen as these systems would be sufficiently conjugated to absorb UV-light emitted from medium-pressure Hg-lamps that are easily integrated with standardized flow reactors such as the Vapourtec UV-150 system (Scheme 2).

**Scheme 2:**
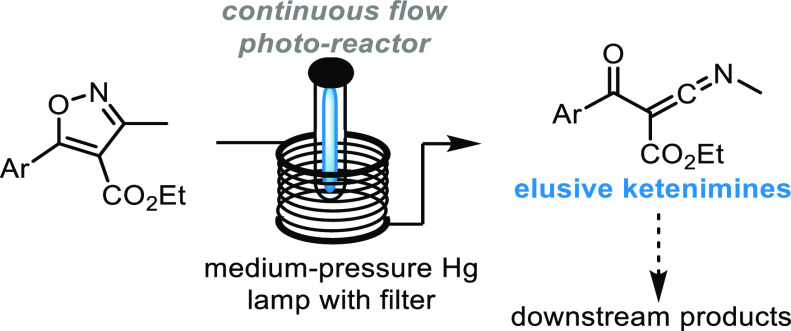
Synthetic Strategy to Photochemically Generate Ketenimines from Isoxazoles

Initial studies using this flow setup in combination with a medium-pressure Hg-lamp (intensity of 90%, ∼135 W input power) and a low-pass filter blocking wavelengths above 400 nm quickly revealed that the isoxazole substrate (10 mM MeCN) was fully consumed within 20 min residence time affording a new product. This material was characterized by the downfield shift of the methyl group in its ^1^H NMR spectrum from 2.5 ppm to 3.7 ppm. The absence of diagnostic resonances confirming the identity of this product prompted us to assign its structure based on derivatization by a hydrogenation reaction. This was achieved by passing a freshly prepared solution of the crude reaction mixture through a H-Cube flow hydrogenation reactor^9^ fitted with a 10% Pd/C cartridge (Scheme 3). Analysis of the ^1^H NMR spectrum showed two new products that were assigned to the corresponding enamines as a mixture of *E*/*Z* isomers (ratio ca. 2:1, see SI for details) that matched previous literature reports.^10^ A final piece of data in support of the proposed ketenimine structures can be seen in their IR spectra, showing a distinct broad band around 2200 cm^–1^ (see SI for details).

**Scheme 3:**
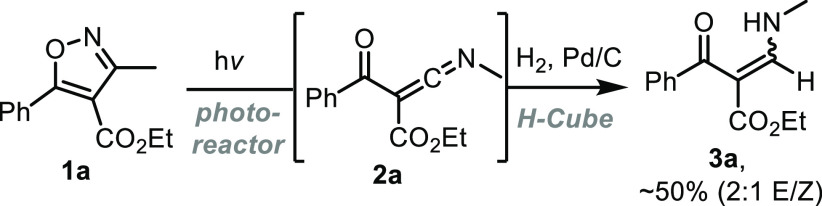
Derivatization into Enamines **3a** via Hydrogenation

Having secured evidence that isoxazoles can indeed be transformed into isolable ketenimines, we next optimized this flow-based transformation (Table 1). This revealed that solutions of 25 mM in MeCN give the best results with shorter residence times of 12.5 min being preferred due to minimized side reactions (entries 1–3). Solvents other than MeCN (acetone, EtOAc, DCM; entries 4–6) are tolerated albeit at lower efficacy. Whereas deoxygenation of the stock solution had little effect on the reaction performance, reduction of the reactor temperature from ∼35 to 10 °C generated fewer colored impurities and higher product yields. The removal of the low pass filter as well as increasing the lamp input power resulted in more decomposition, while the use of a LED emitting at 365 nm (90 W input power) gave no conversion, showcasing the need for UV-B/C light which agrees with UV–vis data of the substrate (λ_max_ 266 nm). Neither the absence of light nor thermal processing afforded the desired ketenimine product. Interestingly, no azirine or oxazole products were observed in the crude reaction mixtures. To reduce the amounts of unidentified side products, conditions leaving small amounts of residual substrate (10–15%) in the reaction mixture were favored going forward.

**Table 1:** Screening of the Reaction Conditions toward **2a**

entry	solvent	*t*_Res_ (min)	lamp	temp (°C)	^1^H NMR yield^c^ (%)
1	MeCN	20	Hg-lamp^a^	35	18
2	MeCN	40	Hg-lamp	35	11
3	MeCN	12.5	Hg-lamp	35	45
4	acetone	12.5	Hg-lamp	35	6
5	EtOAc	12.5	Hg-lamp	35	14
6	DCM	12.5	Hg-lamp	35	10
7	MeCN	12.5	Hg-lamp	10	54
8	MeCN	12.5	365 nm LED	10	0^b^
9	MeCN	12.5	none	25	0
10	MeCN	30	none	85	0

a Used with low-pass filter (<400 nm).

b Some insoluble residue after evaporation of MeCN.

c Using 1,3,5-trimethoxybenzene.

With the initial objective to generate reactive ketenimine species via the photoisomerization of trisubstituted isoxazoles achieved, we proceeded to evaluate the generality of this process. It was found that a variety of isoxazole substrates characterized by differing substitution patterns on the aryl moiety afforded the desired ketenimine products using the optimized reaction conditions (Figure 1). Alkyl, alkoxy, and various halide groups were well tolerated giving the desired ketenimine in synthetically useful yields. A general trend was observed throughout this series whereby the absorbance of the substrate correlates with the yield of the ketenimine product; i.e., substrates with λ_max_ values in the range 260–280 nm gave the highest yields, whereas substrates with λ_max_ values below 240 nm or above 300 nm were less efficient. Isoxazoles substituted by a cinnamyl moiety or nonconjugated appendages (cyclohexyl, phenethyl) did not undergo photoisomerization under these conditions. As expected, longer runs under steady state conditions generated the desired ketenimine in larger amounts (i.e., 2 mmol reaction for **2a** with 57% yield, equiv. to 263 mg, Figure 1) indicating the scalability of the photochemical flow method.^11^

**Figure 1:**
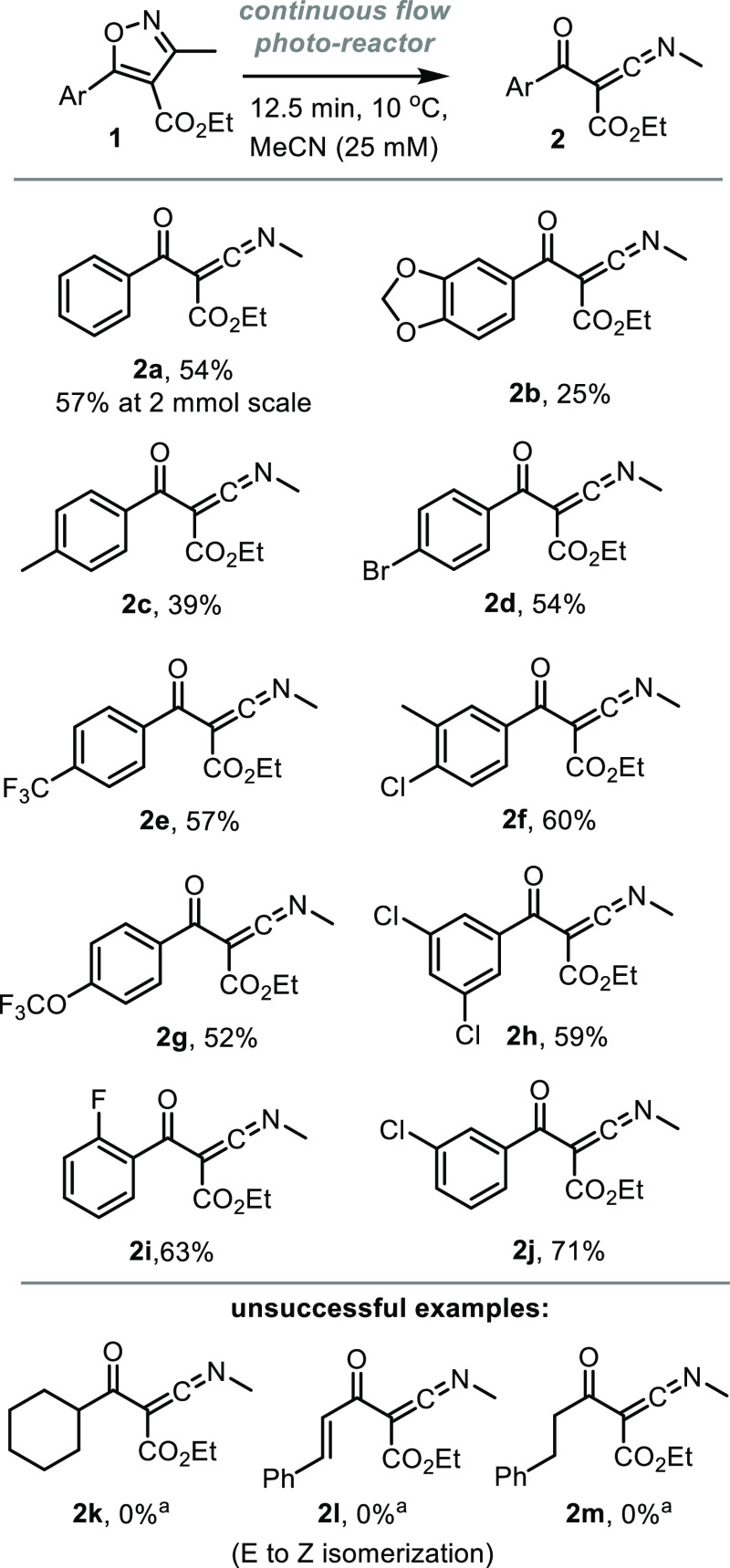
Reaction scope of ketenimine formation (all yields by ^1^H NMR with 1,3,5-trimethoxybenzene as internal standard). ^*a*^No conversion of substrate.

As the isolation of the ketenimine species via silica gel chromatography was hampered by material loss and degradation that we ascribe to the instability of these highly electrophilic products,^12^ we devised a strategy for their direct derivatization into medicinally relevant heterocyclic scaffolds such as pyrazoles. Therefore, the crude ketenimine products were collected into a flask containing either hydrazine-hydrate or phenyl hydrazine as suitable nucleophiles. This simple process rendered the aromatic products through clean reactions by stirring the reaction mixtures at ambient temperature, as shown in Scheme 4 (see SI for details). N–H-Pyrazoles formed as single isomers (**4a**–**f**) whereas mixtures of regioisomers were obtained for trisubstituted aryl pyrazoles (e.g., **5a**/**a′**). Crystallization facilitated the separation of pyrazole regioisomers followed by single crystal X-ray diffraction analysis for pyrazole **5a** which allowed assignment of the other regioisomers in analogy.^13^ In addition, the crystal structure of **5a** confirms the presence of the methyl group on the amine moiety, consistent with its migration from C-3 of the isoxazole substrate, which is a key feature of the underlying mechanism (*vide infra*).

**Scheme 4:**
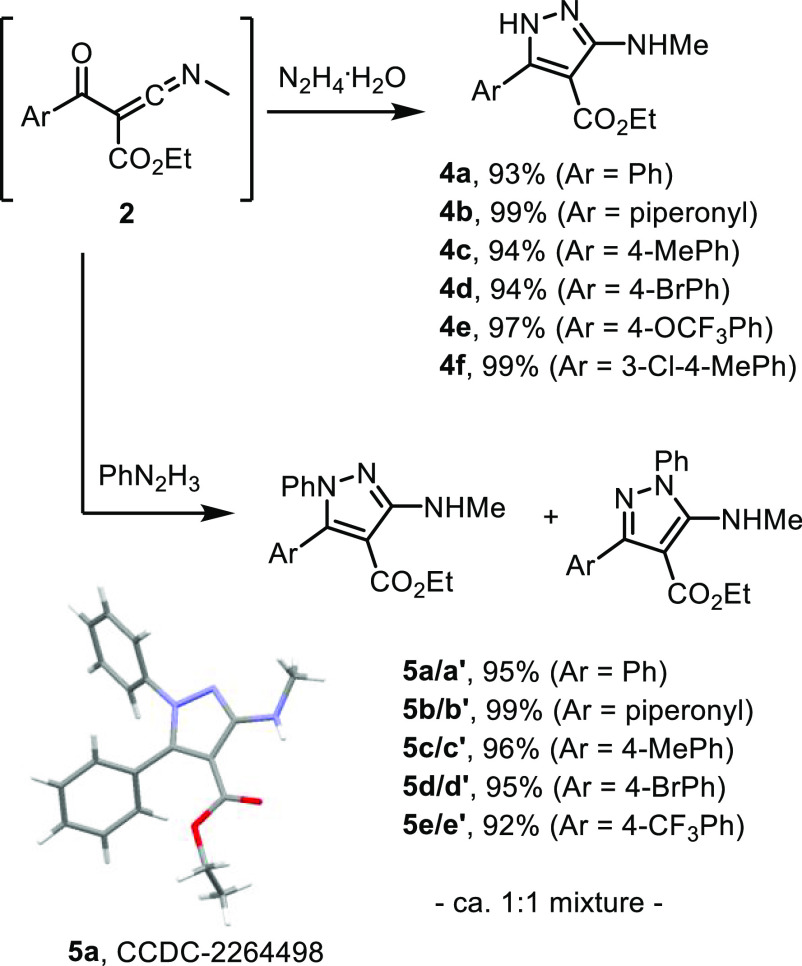
Telescoped Process for the Derivatization of Ketenimines into Densely Functionalized Pyrazoles

Having realized a practical light-driven method for the generation of highly reactive ketenimines from isoxazoles, one remaining task concerned the underlying reaction mechanism that governs this photochemical process. Specifically, we wished to understand why the subtle difference in the substitution pattern of isomeric isoxazoles **1** and **6** has such profound effects in converting these structures into oxazoles (**7**) and ketenimines (**2**), respectively (Figure 2).

**Figure 2:**
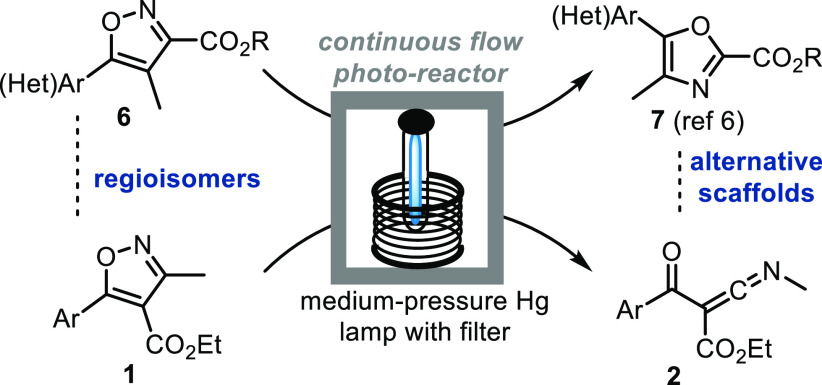
Reaction outcome for isomeric isoxazoles **1** and **6**.

Our mechanistic proposal is based on the photoinduced homolysis of the N–O single bond of the isoxazole substrate analogous to previously reported studies.^2−6^ The following step deviates as C–C bond rotation renders a reactive conformation of the resulting biradical (**Int-1**) in which the oxygen-centered radical can abstract a hydrogen atom from the nearby methyl group via a six-membered transition state (Scheme 5). The intermediary carbon-centered radical rapidly forms an azirine species (**Int-2**) via C–N bond formation. This azirine can either ring open, giving a dipolar nitrilium species that forms the observed ketenimine after proton transfer, or undergo a concerted [1,5]-H shift via a pericyclic process. Crucially, the vicinity of the methyl group adjacent to the imine-type radical in **Int-2** allows for this mechanistic deviation that generates an isomeric azirine compared to the scenario with isoxazole **6** (Figure 2).

**Scheme 5:**
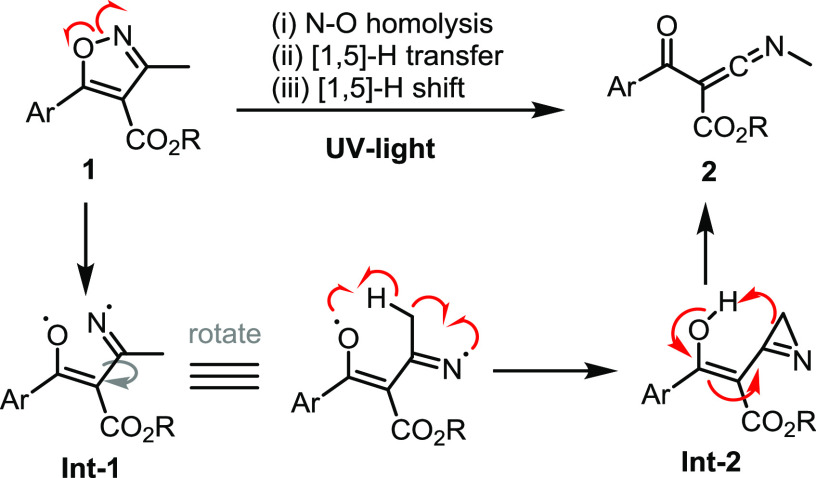
Proposed Reaction Mechanism

In addition, it was found that isoxazole **6** is a colorless oil at room temperature (which slowly solidifies at ca. 15 °C) whereas isoxazole **1** is a crystalline solid, indicating different conformations (i.e., twisting of phenyl and isoxazole rings) and thus altered π-conjugations of these species. This is furthermore visible in the UV–vis spectra of both isomers, showing that isoxazole **1** has a single maximum absorbance at ca. 267 nm, whereas isoxazole **6** is characterized by a maximum absorbance at 273 nm as well as a further area of high absorbance at ca. 244 nm (Figure 3). The slight red-shifting of the absorbance, as well as a second distinct transition at lower wavelengths for isoxazole **1** thus indicates its different photochemical behavior and provides a valuable starting point for future computational studies.

**Figure 3:**
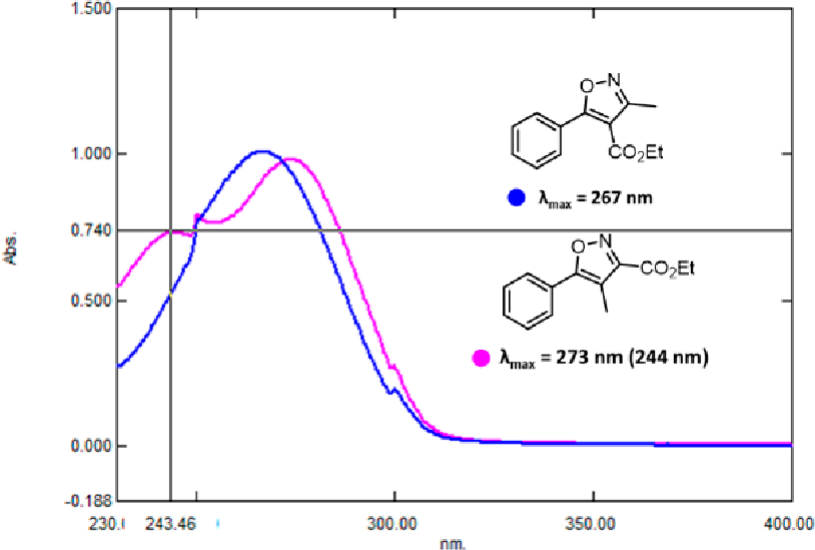
Overlay of UV–vis spectra of the isomeric isoxazoles.

In conclusion, we demonstrate the synthesis of highly reactive ketenimines from trisubstituted isoxazoles under photochemical conditions. The use of a medium-pressure Hg-lamp in combination with a flow reactor setup facilitates the access to these previously elusive reaction products. This approach renders a selection of isolable ketenimines bearing different electron-withdrawing groups. Importantly, these underexplored ketenimine species can serve as valuable intermediates in reactions with hydrazines to generate sets of highly functionalized pyrazoles. Overall, this photochemical approach proves the synthetic accessibility of ketenimines via photolysis of trisubstituted isoxazoles and presents a new methodology for the transposition of isoxazoles into other pharmaceutically relevant heterocycles, such as pyrazoles.

## Data Availability

The data underlying this study are available in the published article and its online Supporting Information.

## Supporting Information Available

Complete experimental procedures and compound characterization (PDF)

## References

[ref1] aSauers R. R.; Hadel L. M.; Scimone A. A.; Stevenson T. A. Photochemistry of 4-Acylisoxazoles. J. Org. Chem. 1990, 55, 4011–4019. 10.1021/jo00300a013.

[ref2] aUllman E. F.; Singh B. Photochemical Transposition of Ring Atoms in Five-Membered Heterocycles. The Photorearrangement of 3,5-Diarylisoxazole. J. Am. Chem. Soc. 1966, 88, 1844–1845. 10.1021/ja00960a066.

[ref3] Pusch S.; Kowalczyk D.; Opatz T. A Photoinduced Cobalt-Catalyzed Synthesis of Pyrroles through in Situ-Generated Acylazirines. J. Org. Chem. 2016, 81, 4170–4178. 10.1021/acs.joc.6b00511. 27081704

[ref4] Wang Q.; Zhang Z.; Zhang X.; Zhang J.; Kang Y.; Peng J. Synthesis of 7a-phenyl-1a,7a-dihydrobenzopyrano[2,3-b]azirin-7-ones via photoisomerization reaction. RSC Adv. 2015, 5, 4788–4794. 10.1039/C4RA12542H.

[ref5] Nunes C. M.; Reva I.; Fausto R. Capture of an Elusive Nitrile Ylide as an Intermediate in Isoxazole–Oxazole Photoisomerization. J. Org. Chem. 2013, 78, 10657–10665. 10.1021/jo4015672. 24073594

[ref6] Bracken C.; Baumann M. Development of a Continuous Flow Photoisomerization Reaction Converting Isoxazoles into Diverse Oxazole Products. J. Org. Chem. 2020, 85, 2607–2617. 10.1021/acs.joc.9b03399. 31927926

[ref7] aGutmann B.; Cantillo D.; Kappe C. O. Continuous-Flow Technology-A Tool for the Safe Manufacturing of Active Pharmaceutical Ingredients. Angew. Chem., Int. Ed. 2015, 54, 6688–6728. 10.1002/anie.201409318. 25989203

[ref8] Cheng H.; Wan J.; Lin M.-I.; Liu Y.; Lu X.; Liu J.; Xu Y.; Chen J.; Tu Z.; Cheng Y.-S.E.; Ding K. Design, Synthesis, and in Vitro Biological Evaluation of 1H-1,2,3-Triazole-4-carboxamide Derivatives as New Anti-influenza A Agents Targeting Virus Nucleoprotein. J. Med. Chem. 2012, 55, 2144–2153. 10.1021/jm2013503. 22332894

[ref9] aBryan M. C.; Wernick D.; Hein C. D.; Petersen J. V.; Eschelbach J. D.; Doherty E. M. Evaluation of a Commercial Packed Bed Flow Hydrogenator for Reaction Screening, Optimization, and Synthesis. Beilstein J. Org. Chem. 2011, 7, 1141–1149. 10.3762/bjoc.7.132. 21915219 PMC3170201

[ref10] Kumar D.; Kommi D. N.; Chopra P.; Ansari M. I.; Chakraborti A. K. L-Proline-Catalyzed Activation of Methyl Ketones or Active Methylene Compounds and DMF-DMA for Syntheses of (2E)-3-Dimethylamino-2- propen-1-ones. Eur. J. Org. Chem. 2012, 2012, 6407–6413. 10.1002/ejoc.201200778.

[ref11] aDonnelly K.; Baumann M. Scalability of photochemical reactions in continuous flow mode. J. Flow Chem. 2021, 11, 223–241. 10.1007/s41981-021-00168-z.

[ref12] Preliminary evidence points towards the formation of amides as hydration products of crude ketenimine samples upon exposure to silica gel (e.g., based on IR, HRMS, NMR data).

[ref13] Data of the crystal structure was deposited with CCDC and is available from www.ccdc.cam.ac.uk/data_request/cif; **5a**, CCDC 2264498.

