# Supplementary material for: Synthesis of Highly Reactive Ketenimines via Photochemical Rearrangement of Isoxazoles
Source: Org Lett. 2023 Aug 24;25(35):6593–7. doi: 10.1021/acs.orglett.3c02556 (PMC10496124; doi:10.1021/acs.orglett.3c02556)
Supplement: Supplementary file 1 — ol3c02556_si_001.pdf [file ol3c02556_si_001.pdf]

Supporting Information

Synthesis of Highly Reactive Ketenimines via Photochemical Rearrangement of Isoxazoles

Cormac Bracken, Marcus Baumann*

School of Chemistry, University College Dublin, Science Centre South, Dublin 4, Dublin, Ireland.

Table of contents:

1. Materials and Methods SI-2
2. Single-Crystal X-ray Crystallography SI-3
3. Experimental Procedures and Spectroscopic Data of all Compounds SI-10
4. Hydrogenation Studies on crude Ketenimine **2a** SI-28
5. Exemplary IR Spectrum of Ketenimine **2j** SI-29
6. Copies of ^{1}H, ^{19}F, ^{13}C as well as HSQC/HMBC NMR Spectra
    a. Isoxazoles **1a**-**1m** SI-30
    b. Ketenimines **2a-j** (crude materials) SI-46
    c. Pyrazoles **4a-f** and **5a/a'-e/e'** SI-61

1. Materials and Methods

Substrates, reagents, and solvents were purchased from Alfa Aesar, Fischer, Fluorochem or Sigma-Aldrich and used as received unless otherwise stated. NMR spectra were recorded at 25 °C using Varian VNMRS 400 MHz, 500 MHz and 600 MHz spectrometers. Deuterated chloroform was acquired from Sigma-Aldrich or Fluorochem and used as supplied. NMR spectra are reported in δ/ppm using the chosen solvent peak as a reference. Data reported for ^{1}H NMR are as follows: chemical shift (δ/ppm) (multiplicity, coupling constant (Hz), integration). Multiplicities are reported as follows: s = singlet, d = doublet, t = triplet, q = quartet, p = pentet, h = heptet, m = multiplet. Data for ^{13}C NMR are reported in terms of chemical shift (δ/ppm) and multiplicity (C, CH, CH_2, or CH_3). Additional experiments, such as gCOSY, HSQC and HMBC were used in the structural assignment. UV absorbance was recorded on an Agilent Technologies Cary 60 UV-Vis spectrometer. TLC was performed on Merck pre-coated silica gel 60 F254 aluminium plates with realization by UV irradiation at 254 nm and $KMnO_4$ stain. Flash chromatography was performed using Macherey-Nagel Silica gel 60 M, with a particle range of 0.04-0.063 mm. IR spectra were recorded with a Bruker Platinum spectrophotometer (neat, ATR sampling), which utilized a universal attenuated total reflectance sampling accessory, where peaks are listed as strong (s, >71% of the tallest signal), medium (m, 21−70% of the tallest signal), or weak (w, <20% of the tallest signal). High-resolution mass spectrometry (HRMS) was performed using the indicated techniques with a micromass LCT orthogonal time-of-flight mass spectrometer with leucine-enkephalin (Tyr-Gly-Phe-Leu) as an internal lock mass or a Bruker Daltronics Apex III 4.7 T using positive electron spray ionisation (+ESI) for HRMS, or a Fisons Instrument VG Autospec using positive electron impact (+EI) for LRMS and using either methanol or acetonitrile as a solvent. All flow reactions were performed while using a Vapourtec E-Series UV-150 photoflow reactor with a medium-pressure mercury lamp (ca. 200-600 nm, + low-pass filter with cut-off at ca. 400 nm) housed inside a tubular reactor coil (volume: 10 mL, made of FEP).

2. Single-Crystal X-ray Crystallography

The X-ray single crystal data for **5a** have been collected at temperature 120.0(2)K using MoKα radiation (λ =0.71073Å) on a Bruker D8Venture (Photon III MM C7 CPAD detector, IµS-microsource, focusing mirrors) 3-circle diffractometer equipped with a Cryostream (Oxford Cryosystems) open-flow nitrogen cryostat. The structure was solved by direct method and refined by full-matrix least squares on F^2 for all data using Olex2 [1] and SHELXTL [2] software. All non-hydrogen atoms were refined in anisotropic approximation, hydrogen atoms were located in the difference Fourier maps and refined isotropically. Crystal data and parameters of refinement are listed in Tables 1-8. Crystallographic data for the structure (see images below, depicted at 50% probability level) have been deposited with the Cambridge Crystallographic Data Centre as supplementary publication CCDC-2264498.

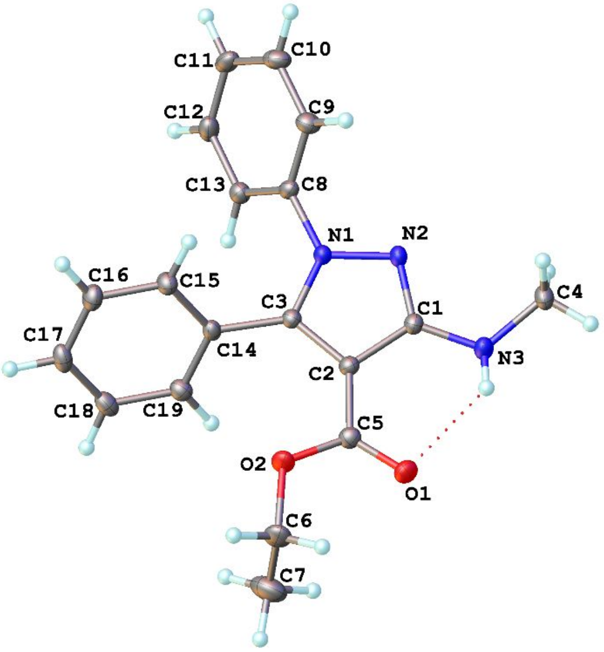

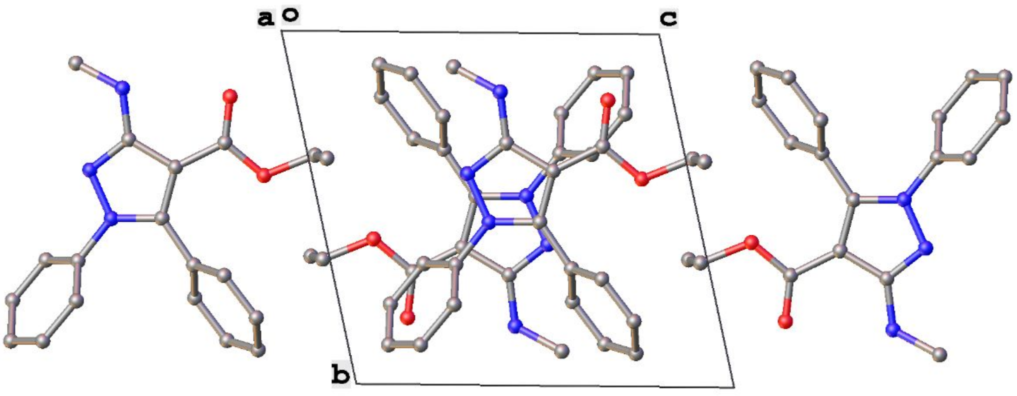

Table 1 Crystal data and structure refinement for CCDC-2264498.	
Identification code	MBaum2
Empirical formula	$C_{19}H_{19}N_3O_2$
Formula weight	321.37
Temperature/K	120.00
Crystal system	triclinic
Space group	P-1
a/Å	8.9357(5)
b/Å	9.5491(6)
c/Å	10.3730(6)
α/°	75.580(2)
β/°	72.585(2)
γ/°	81.586(2)
Volume/Å^3	815.45(8)
Z	2
ρ_{calc}g/cm^3	1.309
μ/mm^{-1}	0.087
F(000)	340.0
Crystal size/mm^3	0.21 × 0.19 × 0.02
Radiation	MoKα (λ = 0.71073)
2Θ range for data collection/°	4.216 to 59.994
Index ranges	-12 ≤ h ≤ 12, -13 ≤ k ≤ 13, -14 ≤ l ≤ 14
Reflections collected	21081
Independent reflections	4746 [R_{int} = 0.0437, R_{sigma} = 0.0418]
Data/restraints/parameters	4746/0/293
Goodness-of-fit on F^2	1.089
Final R indexes [I>=2σ (I)]	R_1 = 0.0546, wR_2 = 0.1169
Final R indexes [all data]	R_1 = 0.0723, wR_2 = 0.1241
Largest diff. peak/hole / e Å^{-3}	0.32/-0.30

Table 2 Fractional Atomic Coordinates (×10^4) and Equivalent Isotropic Displacement Parameters (Å^2×10^3) for CCDC-2264498. U_{eq} is defined as 1/3 of the trace of the orthogonalised U_{IJ} tensor.				
**Atom**	***x***	***y***	***z***	**U(eq)**
O1	2312.8(14)	8110.4(11)	1732.2(11)	23.0(2)
O2	3097.3(14)	5885.5(11)	1273.8(10)	23.4(2)
N1	2406.2(14)	4622.9(12)	5566.6(12)	15.8(2)
N2	2077.1(15)	5992.5(12)	5863.4(12)	17.1(2)
N3	1624.7(17)	8321.5(13)	4541.1(14)	22.0(3)
C1	2011.0(17)	6868.4(15)	4663.0(14)	16.1(3)
C2	2331.1(17)	6094.4(15)	3580.8(14)	15.7(3)
C3	2562.8(16)	4642.1(15)	4226.1(14)	14.7(3)
C4	1625(2)	9018.7(17)	5633.2(17)	21.3(3)
C5	2554.3(17)	6804.9(15)	2129.2(15)	17.5(3)
C6	3218(2)	6473.8(19)	-188.8(16)	27.7(4)
C7	1700(3)	6390(3)	-486(2)	42.3(5)
C8	2448.2(17)	3412.1(15)	6694.1(14)	15.9(3)
C9	3402.3(19)	3426.4(17)	7533.2(15)	21.1(3)
C10	3420(2)	2270.7(18)	8648.7(16)	24.6(3)
C11	2502(2)	1118.6(17)	8923.3(15)	24.0(3)
C12	1554(2)	1125.2(16)	8077.8(16)	22.4(3)
C13	1516.5(18)	2276.6(16)	6960.1(15)	19.0(3)
C14	2948.5(17)	3325.8(14)	3646.0(14)	15.3(3)
C15	4270.7(18)	2390.4(16)	3799.6(15)	19.2(3)
C16	4658.1(19)	1194.3(16)	3186.4(16)	21.8(3)
C17	3744.4(19)	938.2(16)	2412.6(16)	22.1(3)
C18	2412.0(19)	1847.8(17)	2284.5(15)	21.1(3)
C19	2007.4(18)	3035.8(16)	2899.8(15)	19.0(3)

Table 3 Anisotropic Displacement Parameters ($Å^2×10^3$) for CCDC-2264498. The Anisotropic displacement factor exponent takes the form: -$2\pi^2[h^2a^{*2}U_{11}+2hka^*b^*U_{12}+…]$.						
**Atom**	**U_{11}**	**U_{22}**	**U_{33}**	**U_{23}**	**U_{13}**	**U_{12}**
O1	32.2(6)	15.9(5)	19.6(5)	0.0(4)	-8.2(5)	-2.5(4)
O2	34.8(6)	20.6(5)	11.5(5)	-2.9(4)	-3.7(4)	1.5(4)
N1	21.1(6)	13.0(5)	13.7(5)	-3.9(4)	-5.6(5)	1.0(4)
N2	22.3(6)	13.4(5)	16.4(6)	-6.0(4)	-5.5(5)	1.3(5)
N3	34.2(8)	14.0(6)	17.6(6)	-4.1(5)	-7.6(5)	0.7(5)
C1	17.0(7)	14.4(6)	16.9(6)	-4.1(5)	-5.0(5)	0.2(5)
C2	16.8(7)	15.4(6)	14.9(6)	-3.2(5)	-5.1(5)	0.5(5)
C3	15.3(6)	14.9(6)	14.1(6)	-3.7(5)	-4.6(5)	0.3(5)
C4	25.2(8)	17.9(7)	24.0(8)	-9.4(6)	-8.7(6)	1.1(6)
C5	17.5(7)	17.9(7)	15.8(6)	-2.4(5)	-3.4(5)	-1.6(5)
C6	38.9(10)	28.6(8)	11.9(7)	-2.8(6)	-4.1(6)	0.1(7)
C7	47.6(12)	58.4(14)	26.0(9)	-15.4(9)	-16.1(9)	2.7(11)
C8	18.8(7)	15.3(6)	12.1(6)	-3.1(5)	-3.7(5)	2.1(5)
C9	23.1(8)	23.8(7)	18.4(7)	-4.8(6)	-8.4(6)	-2.1(6)
C10	26.8(8)	30.5(8)	17.2(7)	-3.8(6)	-9.9(6)	1.8(6)
C11	32.9(9)	19.8(7)	14.0(7)	-0.6(5)	-4.3(6)	5.1(6)
C12	30.8(8)	15.9(7)	18.0(7)	-4.3(5)	-2.5(6)	-1.6(6)
C13	22.7(7)	17.7(7)	16.8(7)	-5.0(5)	-5.6(6)	0.5(5)
C14	19.4(7)	13.0(6)	12.2(6)	-2.9(5)	-2.1(5)	-1.9(5)
C15	19.8(7)	19.2(7)	19.9(7)	-7.4(5)	-6.1(6)	1.3(5)
C16	22.6(8)	17.9(7)	22.8(7)	-7.0(6)	-2.6(6)	1.7(6)
C17	26.4(8)	18.3(7)	19.6(7)	-8.7(6)	1.5(6)	-4.5(6)
C18	24.4(8)	23.5(7)	16.9(7)	-6.9(6)	-3.2(6)	-7.8(6)
C19	20.3(7)	20.5(7)	16.7(7)	-4.7(5)	-4.7(6)	-2.4(6)

Table 4 Bond Lengths for CCDC-2264498.						
**Atom**	**Atom**	**Length/Å**		**Atom**	**Atom**	**Length/Å**
O1	C5	1.2203(17)		C6	C7	1.495(3)
O2	C5	1.3413(17)		C8	C9	1.392(2)
O2	C6	1.4575(18)		C8	C13	1.387(2)
N1	N2	1.3876(16)		C9	C10	1.388(2)
N1	C3	1.3509(17)		C10	C11	1.391(2)
N1	C8	1.4305(17)		C11	C12	1.388(2)
N2	C1	1.3283(18)		C12	C13	1.390(2)
N3	C1	1.3635(18)		C14	C15	1.397(2)
N3	C4	1.4499(19)		C14	C19	1.396(2)
C1	C2	1.4308(19)		C15	C16	1.394(2)
C2	C3	1.3967(18)		C16	C17	1.385(2)
C2	C5	1.4555(19)		C17	C18	1.387(2)
C3	C14	1.4787(19)		C18	C19	1.388(2)

Table 5 Bond Angles for CCDC-2264498.								
**Atom**	**Atom**	**Atom**	**Angle/˚**		**Atom**	**Atom**	**Atom**	**Angle/˚**
C5	O2	C6	117.08(12)		O2	C6	C7	110.34(15)
N2	N1	C8	117.40(11)		C9	C8	N1	118.54(13)
C3	N1	N2	113.10(11)		C13	C8	N1	120.16(13)
C3	N1	C8	129.40(12)		C13	C8	C9	121.27(13)
C1	N2	N1	103.92(11)		C10	C9	C8	118.85(14)
C1	N3	C4	120.40(13)		C9	C10	C11	120.58(15)
N2	C1	N3	121.95(13)		C12	C11	C10	119.77(14)
N2	C1	C2	112.02(12)		C11	C12	C13	120.40(15)
N3	C1	C2	125.99(13)		C8	C13	C12	119.14(14)
C1	C2	C5	123.33(12)		C15	C14	C3	120.85(13)
C3	C2	C1	104.63(12)		C19	C14	C3	119.63(13)
C3	C2	C5	131.52(13)		C19	C14	C15	119.50(13)

N1	C3	C2	106.31(12)		C16	C15	C14	119.97(14)
N1	C3	C14	123.45(12)		C17	C16	C15	120.17(15)
C2	C3	C14	130.23(12)		C16	C17	C18	119.91(14)
O1	C5	O2	123.59(13)		C17	C18	C19	120.41(14)
O1	C5	C2	123.23(13)		C18	C19	C14	120.00(14)
O2	C5	C2	113.15(12)					

Table 6 Hydrogen Bonds for CCDC-2264498.						
**D**	**H**	**A**	**d(D-H)/Å**	**d(H-A)/Å**	**d(D-A)/Å**	**D-H-A/°**
N3	H3	O1	0.87(2)	2.19(2)	2.8446(17)	132.5(18)

Table 7 Selected Torsion Angles for CCDC-2264498.										
**A**	**B**	**C**	**D**	**Angle/˚**		**A**	**B**	**C**	**D**	**Angle/˚**
C1	C2	C5	O1	-8.6(2)		C6	O2	C5	O1	-7.6(2)
C1	C2	C5	O2	169.49(13)		C7	C6	O2	C5	-87.87(19)
C2	C1	N3	C4	-168.07(14)		C9	C8	N1	N2	-52.89(18)
C2	C3	C14	C15	124.24(17)		C9	C8	N1	C3	130.96(16)
C2	C3	C14	C19	-53.8(2)		C13	C8	N1	N2	125.15(14)
C2	C5	O2	C6	174.39(13)		C13	C8	N1	C3	-51.0(2)
C3	C2	C5	O1	-178.95(15)		C15	C14	C3	N1	-53.9(2)
C3	C2	C5	O2	-0.9(2)		C19	C14	C3	N1	128.00(15)
C4	N3	C1	N2	14.3(2)						

Table 8 Hydrogen Atom Coordinates (Å×10^4) and Isotropic Displacement Parameters (Å^2×10^3) for CCDC-2264498.				
**Atom**	***x***	***y***	***z***	**U(eq)**
H3	1880(30)	8790(20)	3690(20)	39(6)
H4A	2590(30)	8900(30)	5860(20)	55(7)
H4B	810(30)	8650(30)	6510(30)	54(7)
H4C	1360(30)	10060(30)	5370(20)	52(7)
H6A	3540(20)	7500(20)	-440(20)	32(5)
H6B	4080(30)	5850(20)	-680(20)	39(6)
H7A	1800(30)	6700(30)	-1490(30)	54(7)
H7B	830(30)	7030(30)	-10(30)	58(7)
H7C	1390(30)	5380(30)	-200(30)	63(8)
H9	4060(20)	4279(19)	7356(18)	23(5)
H10	4080(30)	2270(20)	9260(20)	39(6)
H11	2490(20)	300(20)	9734(19)	25(5)
H12	890(20)	330(20)	8270(20)	31(5)
H13	840(20)	2304(18)	6361(18)	21(4)
H15	4900(20)	2597(19)	4327(18)	22(5)
H16	5590(20)	557(19)	3306(18)	22(4)
H17	4050(20)	110(19)	1948(19)	23(5)
H18	1760(20)	1690(20)	1730(20)	37(6)
H19	1050(20)	3662(19)	2803(18)	23(5)

1. O. V. Dolomanov, L. J. Bourhis, R. J. Gildea, J. A. K. Howard and H. Puschmann, J. *Appl. Cryst.* (2009), **42**, 339-341.

2. G.M. Sheldrick, *Acta Cryst.* (2008), **A64**, 112-122

3. Experimental Procedures: Synthesis of Isoxazole 1a

To a solution of ethyl acetoacetate (4.86 mL, 38.5 mmol, 1 equiv.) in cyclohexane (8.5 mL, 4.5 M) was added aq. NaOH (1.75 mL, 33% w/w), and the mixture was stirred for 30 min at 0 ℃. Benzoyl chloride (5.7 mL, 49.05 mmol, 1.27 equiv.) and aq. NaOH (9 mL, 33% w/w), respectively, were then added dropwise. The mixture was stirred for 1 hr at 0 ℃, then heated to 40 ℃ and stirred for 1 hr. The reaction mixture was cooled to room temperature and stirred overnight. A yellow precipitate which formed was filtered, washed with Et_2O and cyclohexane, before being dried under suction. Aqueous HCl (1 M, 39 mL, 39 mmol, 1.0 equiv.) was then added, and the solution was stirred before being diluted in EtOAc and washed with water. The organic layer was then further washed with sat. $NaHCO_3$ (50 mL) and brine (50 mL) before the organic layer was dried over anhydrous Na_2SO_4. The organic solvent was then evaporated to afford ethyl 2-benzoyl-3-oxobutanoate as a yellow oil 7.15 g (30.5 mmol, yield 81%) that was of sufficient purity to be used directly in the next step.

To a stirred solution of ethyl 2-benzoyl-3-oxobutanoate (5.3 g, 22.6 mmol) in EtOH (40 mL, 0.57 M) was added $H_2NOH.HCl$ (6.0 g, 86.35 mmol, 3.82 equiv.) in water (30 mL, 2.9 M) dropwise under stirring. Using a DrySyn heating mantle, the reaction mixture was heated to 90 ℃ overnight and allowed to cool before then being diluted in EtOAc (30 mL). The organic layer was washed with water (50 mL) and then brine (50 mL). The organic layer was then dried over anhydrous Na_2SO_4 before being concentrated under vacuum to afford the crude product which was subsequently purified by column chromatography over silica gel (5% EtAOc/cHex) to afford pure compound **1a** as a colourless oil which crystallized upon standing to give a white solid (18.3 mmol, 4.23 g, 81%).

Ethyl 3-methyl-5-phenylisoxazole-4-carboxylate (1a)

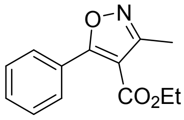

Chemical Formula: $C_{13}H_{13}NO_3$
Exact Mass: 231.0895

Yield = 18.3 mmol, 4.23 g, 81%. Appearance: white solid. **^{1}H NMR (500 MHz, $CDCl_3$) δ/ppm** 7.91-7.88 (m, 2H), 7.53-7.46 (m, 3H), 4.33-4.32 (q, *J* = 7.5 Hz, 2H), 2.52 (s, 3H), 1.31 (t, *J* = 7.5 Hz, 3H). **^{13}C NMR (126 MHz, $CDCl_3$) δ/ppm** 173.0 (C), 162.2 (C), 160.8 (C), 131.2 (CH), 129.2 (2xCH), 128.2 (2xCH), 127.1 (C), 108.2 (C), 60.9 (CH_2), 14.0 (CH_3), 12.3 (CH_3). **IR (neat)** v/cm^{-1} 2992 (w), 1709 (s), 1608 (w), 1442 (m), 1442 (m), 1306 (m), 1098 (s), 767 (s), 703 (s).

HRMS (TOF-EI+) *m/z* [M$^+$] calculated for $C_{13}H_{13}NO_3$ 231.0895; found 231.0897. **Melting point** 54-56 °C. **UV-vis (λ_{max}, MeCN)** = 266 nm.

Synthesis of Benzoyl Chlorides, General Procedure A

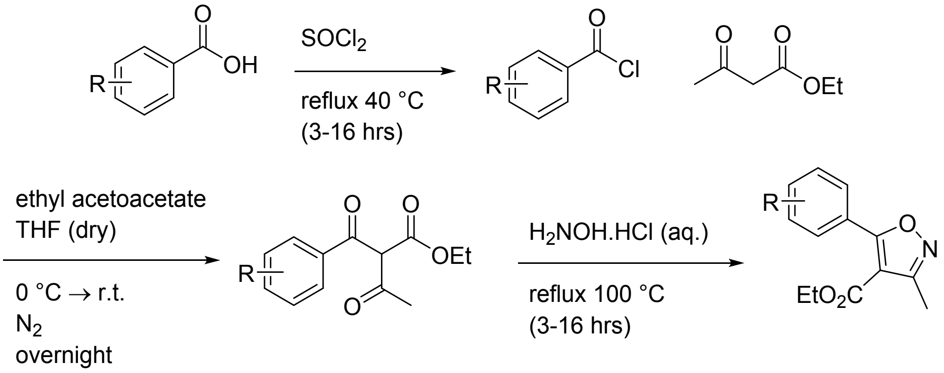

The corresponding benzoic acid (typically 5 mmol) was suspended in DCM (20 mL, 0.25 M) at room temperature. $SOCl_2$ (2 mL, 27.6 mmol, 5.5 equiv.) was then added dropwise along with a few drops of DMF (cat.). The suspension was heated at reflux (3-16 h, using a DrySyn heating mantle) while being monitored by TLC. Following total consumption of the starting material the reaction was ceased and the flask allowed to cool. The solvent was evaporated under reduced pressure before being flushed with N_2. The acid chloride product was used directly in the next step without further purification.

Synthesis of Isoxazoles 1b-1i General Procedure B

A pre-dried flask that had been flushed with N_2 was charged with a stir bar and KO^{t}Bu (5 mmol, 0.561 g, 1.0 equiv.), and left under N_2 for 15 mins. Dry THF (11 mL, 0.45 M) was then added, and the solution was stirred at 0 °C for 30 mins. Ethyl acetoacetate (5 mmol, 0.651 g, 0.638 mL, 1.0 equiv.) was then added and the solution was stirred for a further hour at 0 °C. To the now colourless solution, the appropriate benzoyl chloride reagent (5 mmol, 1.0 equiv.) was added dropwise (or dissolved in minimum volume of dry THF and added dropwise). A precipitate formed in the flask and the reaction was gradually warmed to room temperature before being left to stir overnight under N_2. The solution was then diluted in EtOAc (30 mL) before aqueous HCl (1 M, 1.0 equiv., 5.1 mL) was added. The solution was washed with water, followed by aqueous $NaHCO_3$ and brine (2x50 mL). The organic layer was separated and dried

over anhydrous Na_2SO_4 before the solvent was evaporated to provide the crude adduct as an orange oil which was used directly in the next step without further purification.

To a stirred solution of the freshly prepared adduct (5 mmol, 1.0 equiv.) in EtOH (10 mL, 0.2 M) was added $H_2NOH{\cdot}HCl$ (1.33 g, 19.1 mmol, 3.82 equiv.) in water (11 mL, 1.74 M) dropwise under stirring. Using a DrySyn heating mantle, the reaction mixture was then heated to reflux overnight and allowed to cool before then being diluted in EtOAc (30 mL). The organic layer was washed with water (50 mL) and then brine (50 mL). The organic layer was dried over anhydrous Na_2SO_4 before being concentrated under vacuum to afford the crude product which was subsequently purified by column chromatography using silica gel (5/10% EtAOc/cHex) to afford the pure isoxazole target compound as an oil, which in some cases crystallized over time.

Ethyl 5-(benzo[*d*][1,3]dioxol-5-yl)-3-methylisoxazole-4-carboxylate (1b)

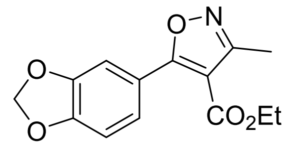

Chemical Formula: $C_{14}H_{13}NO_5$
Exact Mass: 275.0794

Appearance: white solid. Yield = 676 mg, 2.2 mmol, 44%. **^{1}H NMR (500 MHz, $CDCl_3$) δ/ppm** 7.69 (dd, *J* = 8.5, 2.0 Hz, 1H), 7.47 (d, *J* = 1.5 Hz, 1H), 6.87 (d, *J* = 8.5 Hz, 1H), 6.07 (s, 2H), 4.19 (q, *J* = 7.5 Hz, 2H), 2.45 (s, 3H), 1.29 (t, *J* = 7.5 Hz, 3H). **^{13}C NMR (126 MHz, $CDCl_3$) δ/ppm** 166.0 (C), 164.2 (C), 163.2 (C), 152.4 (C), 147.9 (C), 126.3 (C), 122.9 (CH_2), 111.0 (CH), 109.8 (CH), 108.2 (CH), 102.0 (C), 60.2 (CH_2), 19.4 (CH_3), 15.4 (CH_3). **IR (neat)** ν/cm^{-1} 2956 (w), 2917 (w), 1720 (s), 1622 (m), 1479 (s), 1446 (s), 1249 (s), 1095 (s), 1025 (s), 812 (m), 782 (m). **HRMS (TOF-EI+)** *m/z* [M+H$^+$] calculated for $C_{14}H_{14}NO_5$ 276.0872; found 276.0871. **Melting point** 80-82 °C. **UV-vis (λ_{max}, MeCN)** = 309 nm.

Ethyl 3-methyl-5-(p-tolyl)isoxazole-4-carboxylate (1c)

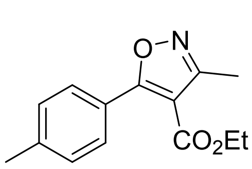

Chemical Formula: $C_{14}H_{15}NO_3$
Exact Mass: 245.1052

Appearance: white solid. Yield = 623 mg, 2.6 mmol, 51%. **^{1}H NMR (500 MHz, $CDCl_3$) δ/ppm** 7.81 (d, *J* = 8.5 Hz, 2H), 7.29 (d, *J* = 8.5 Hz, 2H), 4.32 (q, *J* = 7.5 Hz, 2H), 2.51 (s, 3H), 2.43 (s, 3H), 1.33 (t, *J* = 7.5 Hz, 3H). **^{13}C NMR (125 MHz, $CDCl_3$) δ/ppm** 173.1 (C), 162.3 (C), 160.8 (C), 141.7 (C), 129.1 (2xCH), 129.0 (2xCH), 124.2 (C), 107.8 (C), 60.8 (CH_2), 21.6 (CH_3), 14.1 (CH_3), 12.3 (CH_3). **IR (neat)** ν/cm^{-1} 2993 (w), 1706 (s), 1579 (m), 1473 9m), 1408 (m), 1291 (m), 118 (s), 1090 (s), 824 9m), 783 (s). **HRMS**

(TOF-EI+) *m/z* [M^+] calculated for $C_{14}H_{15}NO_3$ 245.1052; found 245.1046. **Melting point** 42-44 °C. **UV-vis (λ_{max}, MeCN)** = 272 nm.

Ethyl 5-(4-bromophenyl)-3-methylisoxazole-4-carboxylate (1d)

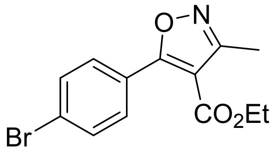

Chemical Formula: $C_{13}H_{12}BrNO_3$
Exact Mass: 309.0001

Appearance: white solid. Yield = 676 mg, 2.2 mmol, 44%. **^{1}H NMR (400 MHz, $CDCl_3$) δ/ppm** 7.79 (dt, *J* = 9.0, 2.4 Hz, 2H), 7.60 (dt, *J* = 9.0, 2.4 Hz, 2H), 4.30 (q, *J* = 7.1 Hz, 2H), 2.49 (s, 3H), 1.31 (t, *J* = 7.1 Hz, 3H). **^{13}C NMR (101 MHz, $CDCl_3$) δ/ppm** 171.8 (C), 162.0 (C), 160.9 (C), 131.6 (2xCH), 130.6 (2xCH), 126.0 (C), 125.9 (C), 108.5 (C), 61.0 (CH_2), 14.1 (CH_3), 12.3 (CH_3). **IR (neat)** ν/cm^{-1} 2988 (w), 2976 (w), 1714 (s), 1606 (m), 1476 (m), 1377 (m), 1234 (m), 1092 (s), 821 (m), 781 (m). **HRMS (TOF-EI+)** *m/z* [M^+H^+] calculated for $C_{13}H_{13}{}^{79}BrNO_3$ 310.0079; found 310.0076. **Melting point** 80-82 °C. **UV-vis (λ_{max}, MeCN)** = 275 nm.

Ethyl 3-methyl-5-(4-(trifluoromethyl)phenyl)isoxazole-4-carboxylate (1e)

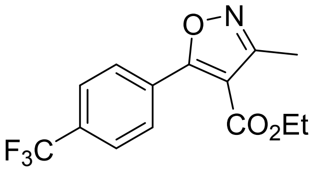

Chemical Formula: $C_{14}H_{12}F_3NO_3$
Exact Mass: 299.0769

Appearance: white solid. Yield = 656 mg, 2.2 mmol, 44%. **^{1}H NMR (400 MHz, $CDCl_3$) δ/ppm** 8.03 (d, *J* = 8.0 Hz, 2H), 7.73 (d, *J* = 8.0 Hz, 2H), 4.31 (q, *J* = 7.0 Hz, 2H), 2.51 (s, 3H), 1.32 (t, *J* = 7.0 Hz, 3H). **^{13}C NMR (101 MHz, $CDCl_3$) δ/ppm** 171.3 (C), 161.8 (C), 160.9 (C), 132.8 (q, $J_{C\text{-}F}$ = 33 Hz, C), 130.3 (C), 129.6 (2xCH), 125.2 (q, $J_{C\text{-}F}$ = 4 Hz, 2xCH), 123.6 (q, $J_{C\text{-}F}$ = 266 Hz, CF_3), 109.3 (C), 61.2 (CH_2), 14.0 (CH_3), 12.3 (CH_3). **^{19}F-NMR (376 MHz, $CDCl_3$) δ/ppm** -61.1. **IR (neat)** ν/cm^{-1} 3036 (w), 1720 (s), 1600 (m), 1417 (m), 1323 (s), 1169 (s), 1129 (s), 1018 (s), 848 (m), 788 (m). **HRMS (TOF-EI+)** *m/z* [M^+H^+] calculated for $C_{14}H_{13}F_3NO_3$ 300.0848; found 300.0845. **Melting point** 51-53 °C. **UV-vis (λ_{max}, MeCN)** = 267 nm.

Ethyl 5-(4-chloro-3-methylphenyl)-3-methylisoxazole-4-carboxylate (1f)

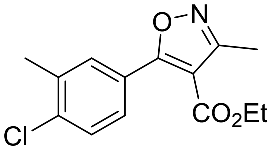

Chemical Formula: $C_{14}H_{14}ClNO_3$
Exact Mass: 279.0662

Appearance: off-white powder. Yield = 1.322 g, 4.7 mmol, 47%. **^{1}H NMR (500 MHz, $CDCl_3$) δ/ppm** 7.80 (d, *J* = 1.5 Hz, 1H), 7.70 (ddd, *J* = 8.5, 2.0, 0.5 Hz, 1H), 7.45 (d, *J* = 8.5 Hz, 1H), 4.32 (q, *J* = 7.5 Hz, 2H), 2.51 (s, 3H), 2.45 (s, 3H), 1.33 (t, *J* = 7.5 Hz, 3H). **^{13}C NMR (125 MHz, $CDCl_3$) δ/ppm** 172.0 (C),

162.1 (C), 160.9 (C), 137.6 (C), 136.3 (C), 131.5 (CH), 129.0 (CH), 127.9 (CH), 125.4 (C), 108.4 (C), 61.0 (CH_2), 20.1 (CH_3), 14.1 (CH_3), 12.3 (CH_3). **IR (neat)** ν/cm^{-1} 2982 (w), 2987 (w), 1720 (s), 1578 (m), 1453 (m), 1310 (m), 1100 (s), 869 (m), 787 (m). **HRMS (TOF-EI+)** *m/z* [M^+H^+] calculated for $C_{14}H_{15}{}^{35}ClNO_3$ 280.0740; found 280.0735. **Melting point** 65-67 °C. **UV-vis (λ_{max}, MeCN)** = 276 nm.

Ethyl 3-methyl-5-(4-(trifluoromethoxy)phenyl)isoxazole-4-carboxylate (1g)

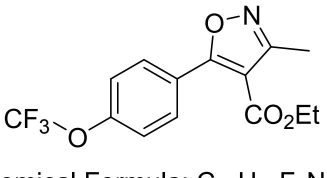

Chemical Formula: $C_{14}H_{12}F_3NO_4$
Exact Mass: 315.0718

Appearance: yellow oil. Yield = 1.35 g, 4.3 mmol, 43%. **^{1}H NMR (400 MHz, $CDCl_3$) δ/ppm** 7.99 (dt, *J* = 9.6, 2.8 Hz, 2H), 7.30-7.34 (m, 2H), 4.33 (q, *J* = 7.1, 2H), 2.51 (s, 3H), 1.33 (t, *J* = 7.1, 3H). **^{13}C NMR (101 MHz, $CDCl_3$) δ/ppm** 171.5 (C), 162.0 (C), 160.8 (C), 151.1 (q, $J_{C\text{-}F}$ = 2 Hz, C), 131.0 (2xCH), 125.5 (C), 120.4 (2CH), 120.3 (d, $J_{C\text{-}F}$ = 261 Hz, OCF_3), 108.6 (C), 61.0 (CH_2), 14.0 (CH_3), 12.3 (CH_3). **^{19}F-NMR (376 MHz, $CDCl_3$) δ/ppm** -57.7. **IR (neat)** ν/cm^{-1} 2966 (w), 1718 (m), 1598 (w), 1419 (w), 1209 (s), 1163 (s), 1092 (s), 855 (m), 786 (m). **HRMS (TOF-EI+)** *m/z* [M^+H^+] calculated for $C_{14}H_{13}F_3NO_4$ 316.0797; found 316.0791. **UV-vis (λ_{max}, MeCN)** = 264 nm.

Ethyl 5-(3,5-dichlorophenyl)-3-methylisoxazole-4-carboxylate (1h)

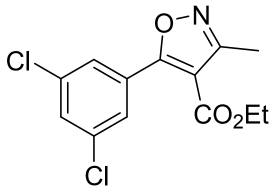

Chemical Formula: $C_{13}H_{11}Cl_2NO_3$
Exact Mass: 299.0116

Appearance: off-white powder. Yield = 0.964 g, 3.2 mmol, 32%. **^{1}H NMR (500 MHz, $CDCl_3$) δ/ppm** 7.85 (d, *J* = 2.5 Hz, 2H), 7.52 (t, *J* = 2.0 Hz, 1H), 4.34 (q, *J* = 7.0 Hz, 2H), 2.52 (s, 3H), 1.36 (t, *J* = 7.0 Hz, 3H). **^{13}C NMR (125 MHz, $CDCl_3$) δ/ppm** 169.6 (C), 161.6 (C), 161.1 (C), 135.1 (C), 131.0 (C), 129.6 (CH), 128.0 (C), 127.5 (2xCH), 109.4 (C), 61.3 (CH_2), 14.0 (CH_3), 12.2 (CH_3). **IR (neat)** ν/cm^{-1} 2984 (w), 1714 9s), 1557 (m), 1438 (m), 1404 (m), 1225 (m), 1122 (s), 835 (m), 784 (s). **HRMS (TOF-EI+)** *m/z* [M^+H^+] calculated for $C_{13}H_{12}{}^{35}Cl_2NO_3$ 300.0914; found 300.0190. **Melting point** 96-99 °C. **UV-vis (λ_{max}, MeCN)** = 268 nm.

Ethyl 5-(2-fluorophenyl)-3-methylisoxazole-4-carboxylate (1i)

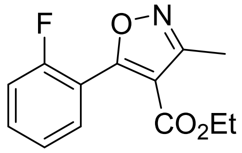

Chemical Formula: $C_{13}H_{12}FNO_3$
Exact Mass: 249.0801

Appearance: pale yellow oil. Yield = 1.55 g, 6.0 mmol, 60%. **^{1}H NMR (400 MHz, $CDCl_3$) δ/ppm** 7.57 (td, *J* = 7.3, 1.8 Hz, 1H), 7.54 – 7.47 (m, 1H), 7.28 – 7.23 (m, 1H), 7.17 (ddd, *J* =

9.8, 8.4, 1.1 Hz, 1H), 4.22 (q, *J* = 7.2 Hz, 2H), 2.51 (s, 3H), 1.18 (t, *J* = 7.2 Hz, 3H). **^{13}C NMR (100 MHz, CDCl$_3$) δ/ppm** 167.9 (C), 161.7 (C), 160.4 (C), 159.7 (d, *J* = 252 Hz, CF), 132.9 (d, *J* = 8.5 Hz, CH), 130.7 (CH), 124.0 (d, *J* = 4 Hz, CH), 116.1 (d, *J* = 14 Hz, C), 116.0 (d, *J* = 22 Hz, CH), 110.9 (C), 60.8 (CH$_2$), 13.8 (CH$_3$), 11.7 (CH$_3$). **^{19}F-NMR (376 MHz, CDCl$_3$) δ/ppm** -110.6 (m). **IR (neat)** ν/cm^{-1} 2983 (w), 1718 (s), 1630 (m), 1598 (m), 1487 (m), 1314 (s), 1242 (s), 1092 (s), 821 (m), 758 (s). **HRMS (TOF-EI+)** *m/z* [M$^+$H$^+$] calculated for $C_{13}H_{13}FNO_3$ 250.0874; found 250.0874.

Ethyl 5-(3-chlorophenyl)-3-methylisoxazole-4-carboxylate (1j)

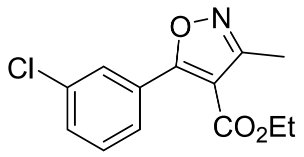

Chemical Formula: $C_{13}H_{12}ClNO_3$
Exact Mass: 265.0506

Appearance: waxy solid. Yield = 1.78 g, 6.5 mmol, 65%. **^{1}H NMR (400 MHz, CDCl$_3$) δ/ppm** 7.89 (t, *J* = 1.9 Hz, 1H), 7.75 (dt, *J* = 7.8, 1.4 Hz, 1H), 7.46 – 7.42 (m, 1H), 7.37 (t, *J* = 7.9 Hz, 1H), 4.28 (q, *J* = 7.1 Hz, 2H), 2.47 (s, 3H), 1.29 (t, *J* = 7.1 Hz, 3H). **^{13}C NMR (100 MHz, CDCl$_3$) δ/ppm** 171.1 (C), 161.8 (C), 160.9 (C), 134.2 (C), 131.1 (CH), 129.5 (CH), 129.2 (CH), 128.6 (C), 127.2 (CH), 108.8 (C), 61.0 (CH$_2$), 14.0 (CH$_3$), 12.2 (CH$_3$). **IR (neat)** ν/cm^{-1} 2983 (w), 1716 (s), 1587 (m), 1563 (m), 1431 (m), 1310 (s), 1229 (s), 1135 (m), 1093 (s), 777 (s), 678 (m). **HRMS (TOF-EI+)** *m/z* [M$^+$H$^+$] calculated for $C_{13}H_{13}{}^{35}ClNO_3$ 266.0578; found 266.0578.

Ethyl 5-cyclohexyl-3-methylisoxazole-4-carboxylate (1k)

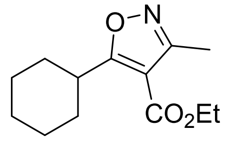

Chemical Formula: $C_{13}H_{19}NO_3$
Exact Mass: 237.1365

Appearance: pale yellow oil. Yield = 330 mg, 1.4 mmol, 28%. **^{1}H NMR (400 MHz, d$_6$-DMSO) δ/ppm** 4.21 (q, *J* = 7.0 Hz, 2H), 3.35 (tt, *J* = 12.0, 3.3 Hz, 1H), 2.30 (s, 3H), 1.86 – 1.71 (m, 4H), 1.70 – 1.62 (m, 1H), 1.48 (qd, *J* = 12.1, 3.0 Hz, 2H), 1.38 -1.28 (m, 3H), 1.26 (t, *J* = 7.1 Hz, 3H). **^{13}C NMR (100 MHz, d$_6$-DMSO) δ/ppm** 181.5 (C), 162.0 (C), 159.7 (C), 107.1 (C), 60.8 (CH$_2$), 37.0 (CH), 30.2 (2CH$_2$), 25.9 (2CH$_2$), 25.6 (CH$_2$), 14.3 (CH$_3$), 11.8 (CH$_3$). **IR (neat)** ν/cm^{-1} 2930 (s), 2854 (m), 1717 (s), 1597 (m), 1450 (m), 1293 (m), 1172 (m), 1099 (s). **HRMS (TOF-EI+)** *m/z* [M$^+$H$^+$] calculated for $C_{13}H_{20}NO_3$ 238.1443; found 238.1438. **UV-vis (λ_{max}, MeCN)** = 223 nm.

Ethyl (*E*)-3-methyl-5-styrylisoxazole-4-carboxylate (1l)

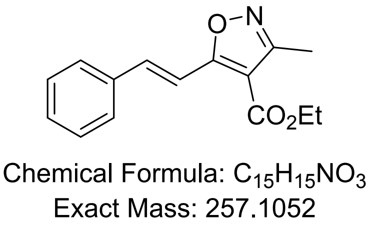

Appearance: white crystalline solid. Yield = 1.021 g, 4.0 mmol, 40%. **^{1}H NMR (500 MHz, $CDCl_3$) δ/ppm** 7.58-7.61 (m, 4H), 7.38-7.43 (m, 3H), 4.39 (q, *J* = 7.1 Hz, 2H), 2.48 (s, 3H), 1.44 (t, *J* = 7.1 Hz, 3H). **^{13}C NMR (125 MHz, $CDCl_3$) δ/ppm** 170.9 (C), 162.3 (C), 160.3 (C), 138.5 (CH), 135.3 (CH), 129.9 (CH), 128.9 (2xCH), 127.7 (2xCH), 112.9 (C), 108.0 (C), 60.8 (CH_2), 14.3 (CH_3), 12.0 (CH_3). **IR (neat)** ν/cm^{-1} 2990 (w), 1701 (s), 1633 (s), 1447 (m), 1308 (m), 1101 (s), 979 (s), 784 (m), 749 (m), 694 (s). **HRMS (TOF-EI+)** *m/z* [M^+H^+] calculated for $C_{15}H_{16}NO_3$ 258.1130; found 258.1125. **Melting point** 103-105 °C. **UV-vis (λ_{max}, MeCN)** = 302 nm.

Ethyl 3-methyl-5-phenethylisoxazole-4-carboxylate (1m)

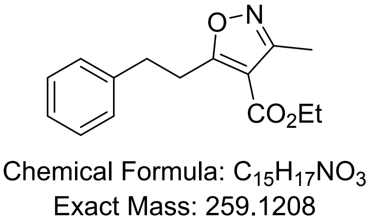

Appearance: pale yellow oil. Yield = 50 mg, 0.19 mmol, 50%. **^{1}H NMR (500 MHz, $CDCl_3$) δ/ppm** 7.32-7.28 (m, 2H), 7.24-7.20 (m, 3H), 4.31 (q, *J* = 7.3 Hz, 2H), 3.38-3.35 (m, 2H), 3.05-3.02 (m, 2H), 2.44 (s, 3H), 1.37 (t, *J* = 7.3 Hz, 3H). **^{13}C NMR (125 MHz, $CDCl_3$) δ/ppm** 177.5 (C), 162.2 (C), 159.9 (C), 134.0 (C), 128.6 (2xCH), 128.3 (2xCH), 126.5 (CH), 107.9 (C), 60.6 (CH_2), 33.4 (CH_2), 29.3 (CH_2), 14.3 (CH_3), 11.8 (CH_3). **IR (neat)** ν/cm^{-1} 2998 (w), 2930 (s), 1705 (s), 1603 (m), 1454 (m), 1298 (m), 1100 (s), 785 (w), 699 (m). **HRMS (TOF-EI+)** *m/z* [M^+H^+] calculated for $C_{15}H_{18}NO_3$ 260.1287; found 260.1282. **UV-vis (λ_{max}, MeCN)** = 200 nm.

Synthesis of Ketenimines, General Procedure C

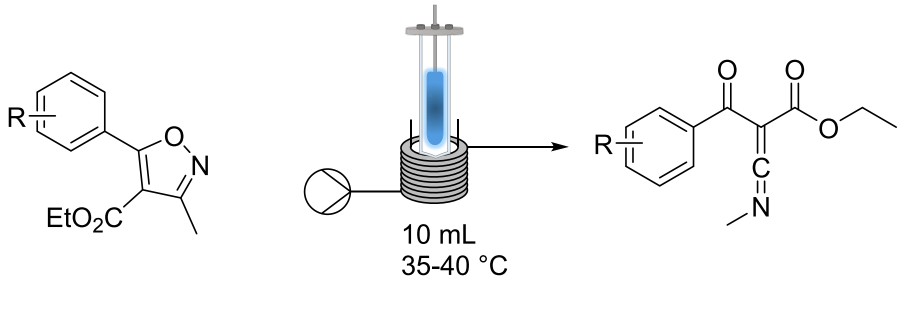

A solution of the chosen isoxazole precursor was prepared in HPLC grade MeCN (10-25 mM) and pumped through the 10 mL photoreactor coil of the UV150 Vapourtec E-series photoflow reactor which housed a medium-pressure Hg-lamp. The reactor used a 90-100% power

setting (equiv. to 135-150 W input power), 0.15 mm i.d. FEP tubing, a filter that excluded light outside 250-400 nm and a flow rate of 0.8 mL/min which gave a 12.5 min residence time. Cooling of the reactor to ca. 10 ˚C was achieved directing compressed air into a Dewar filled with dry ice and passing this into the UV-150 system. A light-yellow solution was typically produced, and the solvent of the crude solution was evaporated to provide a yellow/orange oil which was used directly in the next step without further purification due to the hydrolytic instability of these species.

Using this method, ketenimine **2a** was prepared on 2 mmol scale (20 mM MeCN, 12 min t_{Res}, 57% ^{1}H-NMR yield, equating to 263 mg of product).

Note: The NMR data of the photochemically synthesized ketenimine species is based on the analysis of crude samples of these intermediates using 1,3,5-trimethoxybenzene as an internal standard (relaxation time 25 seconds).

Ethyl 2-benzoyl-3-(methylimino)acrylate (2a)

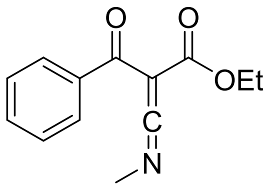

Chemical Formula: $C_{13}H_{13}NO_3$
Exact Mass: 231.0895

Appearance: yellow oil. Yield = 73 mg, 0.32 mmol, 54%. **^{1}H NMR (600 MHz, CDCl$_3$) δ/ppm** 7.59 – 7.56 (m, 2H), 7.46 – 7.34 (m, 3H), 4.17 (d, *J* = 7.1 Hz, 2H), 3.64 (s, 3H), 1.21 (t, *J* = 7.2 Hz, 3H). **^{13}C NMR (150 MHz, CDCl$_3$) δ/ppm** 191.8 (C), 165.7 (C), 142.8 (C, weak), 140.3 (C), 131.2 (CH), 127.9 (2CH), 127.7 (2CH), 68.8 (C), 60.1 (CH_2), 32.9 (CH_3), 14.4 (CH_3). **IR (neat)** ν/cm^{-1} 2992 (w), 2021 (w), 1709 (s), 1480 (m), 1379 (m), 1098 (s), 834 (m), 790 (s). **HRMS (TOF-EI+)** *m/z* [M^+H^+] calculated for $C_{13}H_{14}NO_3$ 232.0974; found 232.0967.

Ethyl 2-(benzo[*d*][1,3]dioxole-5-carbonyl)-3-(methylimino)acrylate (2b)

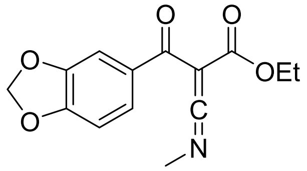

Chemical Formula: $C_{14}H_{13}NO_5$
Exact Mass: 275.0794

Appearance: yellow oil. Yield = 25 mg, 0.091 mmol, 25%. **^{1}H NMR (500 MHz, CDCl$_3$) δ/ppm** 7.51 (dd, *J* = 8.2, 1.8 Hz, 1H), 7.42 (d, *J* = 1.8 Hz, 1H), 6.89 (d, *J* = 8.2 Hz, 1H), 6.03 (s, 2H), 4.31 (q, *J* = 7.1 Hz, 2H), 3.67 (s, 3H), 1.24 (t, *J* = 7.1 Hz, 3H). **IR (neat)** ν/cm^{-1} 2997 (w), 2026 (w), 1717 (m), 1587 (m), 1438 (m), 1247 (s), 1095 (s), 1033 (s), 811 (m), 782 (m). **HRMS (TOF-EI+)** *m/z* [M^+H^+] calculated for $C_{14}H_{14}NO_5$ 276.0872; found 276.0867.

Ethyl 2-(4-methylbenzoyl)-3-(methylimino)acrylate (2c)

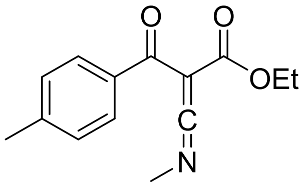

Chemical Formula: $C_{14}H_{15}NO_3$
Exact Mass: 245.1052

Appearance: yellow oil. Yield = 39 mg, 0.16 mmol, 39%. **^{1}H NMR (600 MHz, $CDCl_3$) δ/ppm** 7.50 (d, *J* = 8.1 Hz, 2H), 7.16 (d, *J* = 8.0 Hz, 2H), 4.16 (q, *J* = 7.1 Hz, 2H), 3.63 (s, 3H), 2.35 (s, 3H), 1.22 (t, *J* = 7.3 Hz, 3H). **^{13}C NMR (150 MHz, $CDCl_3$) δ/ppm** 191.3 (C), 165.8 (C), 144.6 (C, weak), 141.8 (C), 137.4 (C), 128.6 (2CH), 128.0 (2CH), 68.7 (C), 60.1 (CH_2), 33.1 (CH_3), 21.5 (CH_3), 14.4 (CH_3). **IR (neat)** ν/cm^{-1} 3096 (w), 2091 (w), 2138 (m), 1752 (m), 1709 (s), 1557 (m), 1299 (s), 1105 (m), 775 (m). **HRMS (TOF-EI+)** *m/z* [$M^{+}H^{+}$] calculated for $C_{14}H_{16}NO_3$ 246.1130; found 246.1122.

Ethyl 2-(4-bromobenzoyl)-3-(methylimino)acrylate (2d)

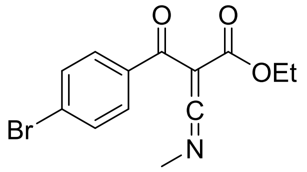

Chemical Formula: $C_{13}H_{12}BrNO_3$
Exact Mass: 309.0001

Appearance: yellow oil. Yield = 54 mg, 0.18 mmol, 54%. **^{1}H NMR (500 MHz, $CDCl_3$) δ/ppm** 7.53-7.51 (m, 2H), 7.48-7.45 (m, 2H), 4.16 (q, *J* = 7.0 Hz, 2H), 3.73 (s, 3H), 1.23 (t, *J* = 7.0 Hz, 3H). **^{13}C NMR (125 MHz, $CDCl_3$) δ/ppm** 191.3 (C), 165.6 (C), 138.9 (C), 131.0 (2xCH), 129.5 (2xCH), 129.1 (C), 125.7 (C), 68.8 (C), 60.2 (CH_2), 32.7 (CH_3), 14.4 (CH_3). **IR (neat)** ν/cm^{-1} 2996 (w), 2229 (m), 1714 (s), 1588 (s), 1308 (s), 1111 (m), 1010 (m), 840 (m), 770 (m). **HRMS (TOF-EI+)** *m/z* [$M^{+}H^{+}$] calculated for $C_{13}H_{13}{}^{79}BrNO_3$ 310.0079; found 310.0073.

Ethyl 3-(methylimino)-2-(4-(trifluoromethyl)benzoyl)acrylate (2e)

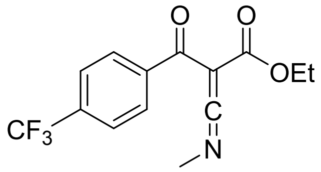

Chemical Formula: $C_{14}H_{12}F_3NO_3$
Exact Mass: 299.0769

Appearance: yellow oil. Yield = 57 mg, 0.19 mmol, 57%. **^{1}H NMR (500 MHz, $CDCl_3$) δ/ppm** 7.66-7.63 (m, 4H), 4.14 (q, *J* = 7.1 Hz, 2H), 3.77 (s, 3H), 1.20 (t, *J* = 7.1 Hz, 3H). **IR (neat)** ν/cm^{-1} 2998 (w), 2281 (s), 1730 (m), 1593 (m), 1409 (m), 1323 (s), 1125 (s), 1067 (s), 850 (m), 736 (m). **HRMS (TOF-EI+)** *m/z* [$M^{+}H^{+}$] calculated for $C_{14}H_{13}F_3NO_3$ 300.0848; found 300.0841.

Ethyl 2-(4-chloro-3-methylbenzoyl)-3-(methylimino)acrylate (2f)

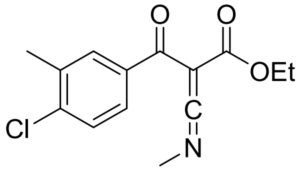

Chemical Formula: $C_{14}H_{14}ClNO_3$
Exact Mass: 279.0662

Appearance: yellow oil. Yield = 60 mg, 0.22 mmol, 60%. **^{1}H NMR (400 MHz, $CDCl_3$) δ/ppm** 7.45 (t, *J* = 1.1 Hz, 1H), 7.33 – 7.31 (m, 2H), 4.15 (q, *J* = 7.1 Hz, 2H), 3.69 (s, 3H), 2.37 (s, 3H),

1.21 (t, *J* = 7.1 Hz, 3H). **^{13}C NMR (100 MHz, $CDCl_3$) δ/ppm** 191.2 (C), 165.6 (C), 141.1 (C, weak), 138.6 (C), 137.4 (C), 135.8 (C), 130.3 (CH), 128.4 (CH), 126.6 (CH), 68.7 (C), 60.1 (CH_2), 32.8 (CH_3), 20.0 (CH_3), 14.4 (CH_2). **IR (neat)** ν/cm^{-1} 3090 (w), 2132 (m), 1744 (m), 1578 (m), 1293 (m), 1100 (m), 882 (s), 775 (m). **HRMS (TOF-EI+)** *m/z* [M$^+$H$^+$] calculated for $C_{14}H_{15}{}^{35}ClNO_3$ 280.0740; found 280.0735.

Ethyl 3-(methylimino)-2-(4-(trifluoromethoxy)benzoyl)acrylate (2g)

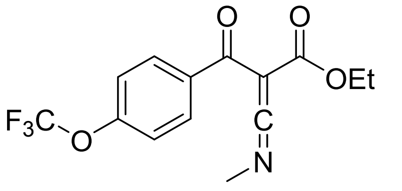

Chemical Formula: $C_{14}H_{12}F_3NO_4$
Exact Mass: 315.0718

Appearance: yellow oil. Yield = 52 mg, 0.17 mmol, 52%. **^{1}H NMR (500 MHz, $CDCl_3$) δ/ppm** 7.63 (d, *J* = 8.4 Hz, 2H), 7.21 (d, *J* = 8.3 Hz, 2H), 4.15 (q, *J* = 7.3 Hz, 2H), 3.74 (s, 3H), 1.21 (t, *J* = 7.1 Hz, 3H). **^{13}C NMR (125 MHz, $CDCl_3$) δ/ppm** 191.1 (C), 165.6 (C), 151.0 (C), 138.5 (C), 129.7 (2CH), 119.8 (2CH), 68.8 (C), 60.1 (CH_2), 32.6 (CH_3), 14.3 (CH_3); 2 C resonances not identified. **^{19}F NMR (470 MHz, $CDCl_3$) δ/ppm** -57.7 (s). **IR (neat)** ν/cm^{-1} 3083 (w), 2260 (m), 2097 (w), 1755 (m), 1731 (m), 1600 (m), 1150 (s), 909 (m), 854 (m), 731 (m). **HRMS (TOF-EI+)** *m/z* [M$^+$H$^+$] calculated for $C_{14}H_{13}F_3NO_4$ 316.0797; found 316.0790.

Ethyl 2-(3,5-dichlorobenzoyl)-3-(methylimino)acrylate (2h)

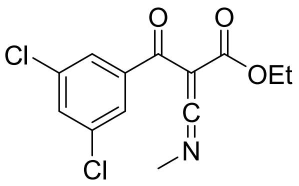

Chemical Formula: $C_{13}H_{11}Cl_2NO_3$
Exact Mass: 299.0116

Appearance: yellow oil. Yield = 55 mg, 0.18 mmol, 59%. **^{1}H NMR (500 MHz, $CDCl_3$) δ/ppm** 7.45 – 7.35 (m, 3H), 4.15 (q, *J* = 7.1 Hz, 2H), 3.82 (s, 3H), 1.21 (t, *J* = 7.2 Hz, 3H). **^{13}C NMR (125 MHz, $CDCl_3$) δ/ppm** 190.2 (C), 165.4 (C), 142.8 (C), 134.5 (2C), 133.5 (C, weak), 130.6 (CH), 126.2 (2CH), 68.5 (C), 60.4 (CH_2), 32.6 (CH_3), 14.5 (CH_3). **IR (neat)** ν/cm^{-1} 2998 (2), 2269 (m), 1698 (s), 1561 (s), 1414 (m), 1299 (s), 1097 (m), 863 (m), 767 (m). **HRMS (TOF-EI+)** *m/z* [M$^+$H$^+$] calculated for $C_{13}H_{12}{}^{35}Cl_2NO_3$ 300.0194; found 300.0185.

Ethyl 2-(2-fluorobenzoyl)-3-(methylimino)acrylate (2i)

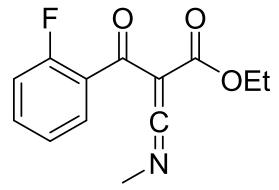

Chemical Formula: $C_{13}H_{12}FNO_3$
Exact Mass: 249.0801

Appearance: yellow oil. Yield = 98 mg, 0.40 mmol, 63%. **^{1}H NMR (400 MHz, $CDCl_3$) δ/ppm** 7.40 – 7.31 (m, 2H), 7.14 (td, *J* = 7.6, 1.3 Hz, 1H), 7.01 (dd, *J* = 10.0, 8.2 Hz, 1H), 4.09 (q, *J* = 7.1 Hz, 2H), 3.74 (s, 3H), 1.14 (t, *J* = 7.2 Hz, 3H). **^{13}C**

NMR (100 MHz, $CDCl_3$) δ/ppm 189.1 (C), 165.2 (C), 159.2 (d, *J* = 255 Hz, CF), 135.7 (C, weak), 131.6 (d, *J* = 8.4 Hz, CH), 129.1 (d, *J* = 3 Hz, CH), 123.9 (d, *J* = 4 Hz, CH), 115.2 (d, *J* = 22 Hz, CH), 71.1 (C), 60.0 (CH_2), 32.3 (CH_3), 14.3 (CH_3); one resonance could not be assigned. **^{19}F NMR (376 MHz, $CDCl_3$) δ/ppm** -114.9 (m). **IR (neat)** ν/cm^{-1} 2983 (w), 2260 (broad), 1710 (s), 1610 (m), 1451 (m), 1310 (s), 1226 (s), 1095 (s), 1019 (m), 757 (s), 649 (m). **HRMS (TOF-EI+)** *m/z* [M^+H^+] calculated for $C_{13}H_{13}FNO_3$ 250.0874; found 250.0877.

Ethyl 2-(3-chlorobenzoyl)-3-(methylimino)acrylate (2j)

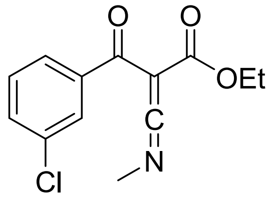

Chemical Formula: $C_{13}H_{12}ClNO_3$
Exact Mass: 265.0506

Appearance: yellow oil. Yield = 70 mg, 0.27 mmol, 71%. **^1H NMR (400 MHz, $CDCl_3$) δ/ppm** 7.53 (t, *J* = 1.9 Hz, 1H), 7.44 (dt, *J* = 7.7, 1.4 Hz, 1H), 7.40 (ddd, *J* = 6.7, 2.8, 1.2 Hz, 1H), 7.31 (d, *J* = 7.8 Hz, 1H), 4.15 (q, *J* = 7.1 Hz, 2H), 3.72 (s, 3H), 1.20 (t, *J* = 7.1 Hz, 3H). **^{13}C NMR (100 MHz, $CDCl_3$) δ/ppm** 190.8 (C), 165.5 (C), 141.9 (C), 139.3 (C, weak), 133.8 (C), 130.9 (CH), 129.1 (CH), 127.8 (CH), 125.9 (CH), 68.9 (C), 60.2 (CH_2), 32.7 (CH_3), 14.3 (CH_3). **IR (neat)** ν/cm^{-1} 2982 (w), 2263 (broad), 1719 (s), 1606 (m), 1567 (m), 1415 (m), 1349 (m), 1307 (s), 1219 (m), 1118 (m), 1026 (m), 771 (m). **HRMS (TOF-EI+)** *m/z* [M^+H^+] calculated for $C_{13}H_{13}{}^{35}ClNO_3$ 266.0578; found 266.0579.

Synthesis of *N*-Methyl-Pyrazoles and *N*H-Pyrazoles, General Procedure D

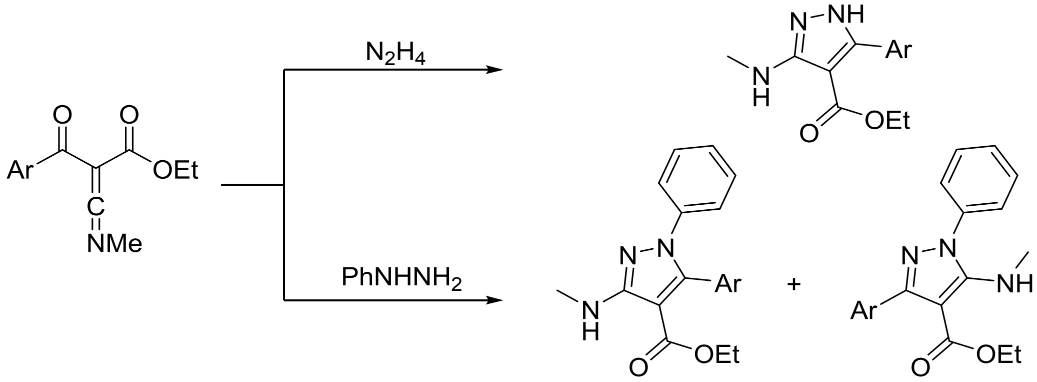

The previously prepared ketenimine (typically 0.1-0.2 mmol scale; 1.0 equiv.) was redissolved in MeCN (1 mL, ca. 0.5 M) and stirred in a vial. To this solution was added 2.0 equiv. of the appropriate reaction partner (hydrazine, *N*-phenyl hydrazine, $H_2NOH.HCl$ (+few drops of NEt_3)). The vial was capped and allowed to stir overnight during which time a precipitate would usually form. The suspension was then diluted in EtOAc and washed with aq. $NaHCO_3$ (50 mL) and brine (50 mL). The organic layer then dried over anhydrous Na_2SO_4 before being concentrated under vacuum by rotary evaporation, affording the crude product. The crude product(s) was then purified by column chromatography on silica gel (1-10% EtOAc/cHex, or 1% DCM/MeOH) giving the pure target compound(s). *N*-Phenyl pyrazoles normally displayed identical R_f values on TLC, and hence required isolation of the crude isomeric mixture. The yield for the combined isomeric product mixture is shown in these cases. To obtain pure samples of each *N*-phenyl pyrazole of a given isomeric pair, the isolated isomeric mixture was crystallized from ice-cold MeOH to the 1,5-diaryl *N*-phenyl pyrazole isomers, with further purification by column chromatography (1-10% EtAOc/cHex, or 1% DCM/MeOH) then applied to the remaining isomer if necessary.

Ethyl 3-(methylamino)-5-phenyl-1*H*-pyrazole-4-carboxylate (4a)

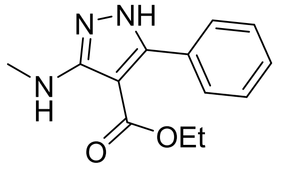

Chemical Formula: $C_{13}H_{15}N_3O_2$
Exact Mass: 245.1164

Appearance: off-white solid. Yield = 45 mg, 0.18 mmol, 93%. **^{1}H NMR (500 MHz, CDCl$_3$) δ/ppm** 7.55-7.53 (m, 2H), 7.40-7.35 (m, 3H), 5.81-5.87 (m, NH), 4.17 (q, *J* = 7.1 Hz, 2H), 2.92 (d, *J* = 4.8 Hz, 3H), 1.17 (t, *J* = 7.1 Hz, 3H). **^{13}C NMR (125 MHz, CDCl$_3$) δ/ppm** 165.0 (C), 158.0 (C), 148.6 (C), 130.7 (C), 129.1 (2CH), 129.0 (CH), 127.9 (2CH), 110.0 (C), 59.5 (CH$_2$), 29.6 (CH$_3$), 14.1 (CH$_3$). **IR (neat)** v/cm^{-1} 3356 (w), 2926 (w), 1657 (s), 1516 9m), 1382 (m), 1282 (s), 1125 (m), 972 (m),

766 (m), 699 (s). **HRMS (TOF-EI+)** *m/z* [M^+H^+] calculated for $C_{13}H_{15}N_3O_2$ 246.1243; found 246.1237. **Melting point** 208-210 °C. **R*f*** = 0.2 (2% MeOH/DCM).

Ethyl 5-(benzo[*d*][1,3]dioxol-5-yl)-3-(methylamino)-1*H*-pyrazole-4-carboxylate (4b)

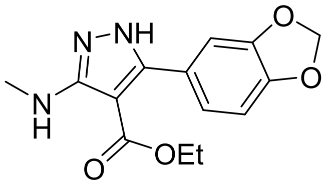

Chemical Formula: $C_{14}H_{15}N_3O_4$
Exact Mass: 289.1063

Appearance: dark yellow solid. Yield = 27 mg, 0.1 mmol, 99%. **^{1}H NMR (500 MHz, $CDCl_3$) δ/ppm** 7.02-6.98 (m, 2H), 6.77 (d, *J* = 8.1 Hz, 1H), 5.99 (s, 2H), 5.81-5.87 (m, NH), 4.19 (q, *J* = 7.1 Hz, 2H), 2.92 (d, *J* = 5.0 Hz, 3H), 1.22 (t, *J* = 7.1 Hz, 3H). **^{13}C NMR (125 MHz, $CDCl_3$) δ/ppm** 164.9 (C), 157.8 (C), 148.2 (C), 147.1 (C), 124.5 (C), 123.0 (CH), 110.0 (C), 109.9 (CH), 107.8 (CH), 101.2 (CH_2), 93.6 (C), 59.5 (CH_2), 29.6 (CH_3), 14.2 (CH_3). **IR (neat)** ν/cm^{-1} 3426 (w), 3196 (w), 1677 (s), 1570 (m), 1489 (s), 1404 (m), 1240 (s), 1144 (s), 1096 (s), 858 (m), 783 (m). **HRMS (TOF-EI+)** *m/z* [M^+H^+] calculated for $C_{14}H_{16}N_3O_4$ 290.1141; found 290.1133. **Melting point** 148-150 °C. **R*f*** = 0.25 (2% MeOH/DCM).

Ethyl 3-(methylamino)-5-(*p*-tolyl)-1*H*-pyrazole-4-carboxylate (4c)

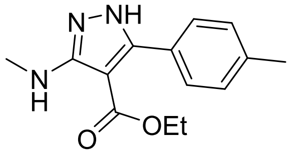

Chemical Formula: $C_{14}H_{17}N_3O_2$
Exact Mass: 259.1321

Appearance: yellow solid. Yield = 41 mg, 0.16 mmol, 94%. **^{1}H NMR (500 MHz, $CDCl_3$) δ/ppm** 7.43 (d, *J* = 8.1 Hz, 2H), 7.17 (d, *J* = 8.1 Hz, 2H), 5.79 (s, NH), 4.18 (q, *J* = 7.1 Hz, 2H), 2.92 (d, *J* = 2.9 Hz, 3H), 2.38 (s, 3H), 1.20 (t, *J* = 7.1 Hz, 3H). **^{13}C NMR (125 MHz, $CDCl_3$) δ/ppm** 165.1 (C), 158.1 (C), 148.4 (C), 138.9 (C), 129.0 (2xCH), 128.5 (2xCH), 127.7 (C), 93.9 (C), 59.5 (CH_2), 29.7 (CH_3), 21.4 (CH_3), 14.2 (CH_3). **IR (neat)** ν/cm^{-1} 3300 (m), 2998 (w), 1650 (s), 1510 (m), 1320 (m), 1292 (m), 1153 (s), 1017 (m), 845 (m), 782 (m). **HRMS (TOF-EI+)** *m/z* [M^+H^+] calculated for $C_{14}H_{18}N_3O_2$ 260.1399; found 260.1392. **Melting point** 151-153 °C. **R*f*** = 0.25 (2% MeOH/DCM).

Ethyl 5-(4-bromophenyl)-3-(methylamino)-1*H*-pyrazole-4-carboxylate (4d)

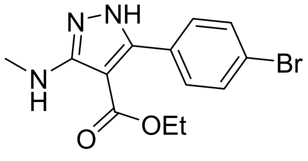

Chemical Formula: $C_{13}H_{14}BrN_3O_2$
Exact Mass: 323.0269

Appearance: dark yellow solid. Yield = 52 mg, 0.16 mmol, 94%. **^{1}H NMR (500 MHz, $CDCl_3$) δ/ppm** 7.46 (dt, *J* = 9.0, 2.4 Hz, 2H), 7.38 (dt, *J* = 9.0, 2.4 Hz, 2H), 5.9 (s, NH), 4.18 (q, *J* = 7.2 Hz, 2H), 2.89 (s, 3H), 1.19 (t, *J* = 7.2 Hz, 3H). **^{13}C NMR (125 MHz, $CDCl_3$) δ/ppm** 164.9 (C), 157.0 (C), 148.7 (C),

130.9 (4xCH), 130.4 (C), 123.1 (C), 93.1 (C), 59.7 (CH_2), 29.8 (CH_3), 14.3 (CH_3). **IR (neat)** ν/cm^{-1} 3293 (m), 2996 (w), 1647 (s), 1508 (m), 1407 (m), 1319 (m), 1152 (s), 963 (m), 835 (m), 781 (m). **HRMS (TOF-EI+)** *m/z* [M^+H^+] calculated for $C_{13}H_{15}{}^{79}BrN_3O_2$ 324.0348; found 324.0340. **Melting point** 145-147 °C. **R*f*** = 0.2 (2% MeOH/DCM).

Ethyl 3-(methylamino)-5-(4-(trifluoromethoxy)phenyl)-1*H*-pyrazole-4-carboxylate (4e)

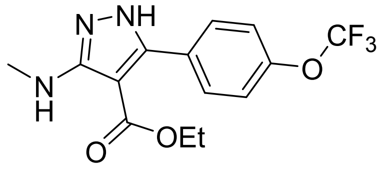

Chemical Formula: $C_{14}H_{14}F_3N_3O_3$
Exact Mass: 329.0987

Appearance: orange solid. Yield = 53 mg, 0.15 mmol, 97%. **^{1}H NMR (400 MHz, $CDCl_3$) δ/ppm** 7.55 (dt, *J* = 9.5, 2.8 Hz, 2H), 7.18 (dt, *J* = 9.5, 2.8 Hz, 2H), 5.98 (br s, NH), 4.17 (q, *J* = 7.2 Hz, 2H), 2.91 (d, *J* = 5.0 Hz, 3H), 1.16 (t, *J* = 7.2 Hz, 3H). **^{13}C NMR (101 MHz, $CDCl_3$) δ/ppm** 164.7 (C), 156.7 (C), 149.5 (C), 148.9 (C), 130.8 (2xCH), 130.4 (C), 120.5, (q, J_{C-F} = 259 Hz, OCF_3), 120.0 (2xCH), 93.0 (C), 59.6 (CH_2), 29.6 (CH_3), 14.0 (CH_3). **^{19}F-NMR (376 MHz, $CDCl_3$) δ/ppm** -57.9. **IR (neat)** ν/cm^{-1} 3294 (m), 2876 (w), 1641 (m), 1523 (m), 1431 (m), 1217 (s), 1153 (s), 1016 (m), 972 (m), 857 (m), 784 (m). **HRMS (TOF-EI+)** *m/z* [M^+H^+] calculated for $C_{14}H_{14}F_3N_3O_3$ 330.1066; found 330.1062. **Melting point** 171-173 °C. **R*f*** = 0.25 (2% MeOH/DCM).

Ethyl 5-(3-chloro-4-methylphenyl)-3-(methylamino)-1*H*-pyrazole-4-carboxylate (4f)

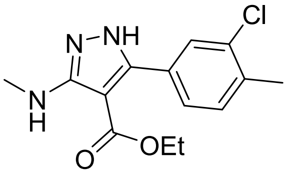

Chemical Formula: $C_{14}H_{16}ClN_3O_2$
Exact Mass: 293.0931

Appearance: orange solid. Yield = 63 mg, 0.22 mmol, 99%. **^{1}H NMR (500 MHz, $CDCl_3$)** 7.33-7.35 (m, 1H), 7.27-7.22 (m, 2H), 5.95 (s, NH), 4.18 (t, *J* = 7.2 Hz, 2H), 2.88 (d, *J* = 5.1 Hz, 3H), 2.32 (s, 3H), 1.19 (t, *J* = 7.2 Hz, 3H). **^{13}C NMR (125 MHz, $CDCl_3$) δ/ppm** 164.9 (C), 157.2 (C), 135.3 (C), 134.9 (C), 131.7 (CH), 131.1 (C), 129.9 (C), 128.3 (CH), 127.9 (CH), 93.0 (C), 59.5 (CH_2), 29.7 (CH_3), 19.9 (CH_3), 14.2 (CH_3). **IR (neat)** ν/cm^{-1} 3298 (m), 2982 (w), 1642 (s), 1517 (m), 1432 (m), 1298 (m), 1154 (s), 1046 (m), 840 (m), 785 (m). **HRMS (TOF-EI+)** *m/z* [M^+H^+] calculated for $C_{14}H_{17}{}^{35}ClN_3O_2$ 294.1009; found 294.1002. **Melting point** 53-55 °C. **R*f*** = 0.2 (2% MeOH/DCM).

Ethyl 3-(methylamino)-1,5-diphenyl-1*H*-pyrazole-4-carboxylate (5a)

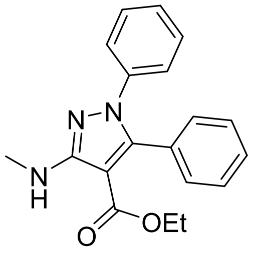

Chemical Formula: $C_{19}H_{19}N_3O_2$
Exact Mass: 321.1477

Appearance: white solid. Yield = 62 mg, 0.2 mmol, 47%. **^{1}H NMR (400 MHz, $CDCl_3$) δ/ppm** 7.13-7.34 (m, 10H), 5.60-5.66 (m, NH), 4.08 (q, *J* = 7.1 Hz, 2H), 3.06 (d, *J* = 5.0 Hz, 3H), 1.04 (t, *J* = 7.1 Hz, 3H). **^{13}C NMR (101 MHz, $CDCl_3$) δ/ppm** 164.9 (C), 158.6 (C), 145.3 (C), 139.5 (C), 130.4 (2xCH), 130.2 (C), 128.7 (CH), 128.6 (2xCH), 127.7 (2xCH), 127.0 (CH), 125.2 (2xCH), 98.9 (C), 59.5 (CH_2), 29.7 (CH_3), 13.9 (CH_3). **IR (neat)** ν/cm^{-1} 3329 (w), 2979 (w), 1665 (s), 1556 (s), 1417 (m), 1250 (m), 1037 (m) 841 (m), 760 (s). **HRMS (TOF-EI+)** *m/z* [M^+H^+] calculated for $C_{19}H_{20}N_3O_2$ 322.1556; found 322.1549. **Melting point** 94-96 °C. **R*f*** = 0.3 (15% EtOAc/cHex).

Ethyl 5-(methylamino)-1,3-diphenyl-1*H*-pyrazole-4-carboxylate (5a')

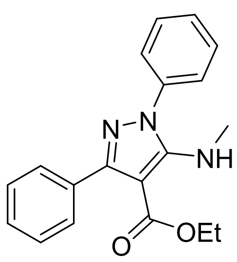

Chemical Formula: $C_{19}H_{19}N_3O_2$
Exact Mass: 321.1477

Appearance: yellow oil. Yield = 62 mg, 0.2 mmol, 47%. **^{1}H NMR (400 MHz, $CDCl_3$) δ/ppm** 7.66-7.60 (m, 4H), 7.49-7.45 (m, 2H), 7.40-7.35 (m, 4H), 6.56-6.62 (m, NH), 4.19 (q, *J* = 7.2 Hz, 2H), 2.59 (d, *J* = 5.8 Hz, 3H), 1.17 (t, *J* = 7.2 Hz, 3H). **^{13}C NMR (101 MHz, $CDCl_3$) δ/ppm** 165.5 (C), 154.5 (C), 152.7 (C), 139.6 (C), 133.4 (C), 129.5 (2CH), 129.0 (2CH), 128.13 (CH), 128.09 (CH), 127.4 (2CH), 125.4 (2CH), 94.9 (C), 59.6 (CH_2), 32.5 (CH_3), 14.0 (CH_3). **IR (neat)** ν/cm^{-1} 3421 (w), 2961 (w), 1666 (s), 1562 (m), 1419 (m), 1255 (m), 1088 (s), 768 (m), 714 (m). **HRMS (TOF-EI+)** *m/z* [M^+H^+] calculated for $C_{19}H_{20}N_3O_2$ 322.1556; found 322.1552. **R*f*** = 0.3 (15% EtOAc/cHex).

Ethyl 5-(benzo[*d*][1,3]dioxol-5-yl)-3-(methylamino)-1-phenyl-1*H*-pyrazole-4-carboxylate (5b)

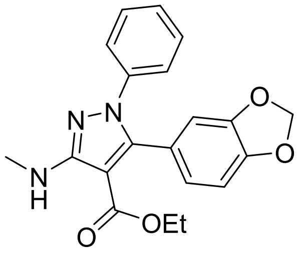

Chemical Formula: $C_{20}H_{19}N_3O_4$
Exact Mass: 365.1376

Appearance: white solid. Yield = 32 mg, 0.09 mmol, 49%. **^{1}H NMR (400 MHz, $CDCl_3$) δ/ppm** 7.26-7.16 (m, 5H), 6.73-6.70 (m, 2H), 6.67-6.64 (m, 1H), 5.96 (s, 2H), 5.55-5.60 (m, NH), 4.13 (q, *J* = 7.2 Hz, 2H), 3.03 (d, *J* = 5.0 Hz, 3H), 1.11 (t, *J* = 7.2 Hz, 3H). **^{13}C NMR (101 MHz, $CDCl_3$) δ/ppm** 164.8 (C), 158.5 (C), 148.0 (C), 147.0 (C), 144.9 (C), 139.5 (C), 128.7 (2xCH), 127.0 (CH), 125.1 (2xCH), 124.6 (CH), 123.5 (C), 110.9 (CH), 107.8 (CH), 101.2 (CH_2), 98.8 (C), 59.5 (CH_2), 29.6 (CH_3), 14.1 (CH_3). **IR (neat)** ν/cm^{-1} 3422 (w),

2998 (w), 2873 (w), 1677 (s), 1546 (m), 1466 (s), 1234 (s), 1032 (s), 877 (m), 784 (m). **HRMS (TOF-EI+)** *m/z* [M$^+$H$^+$] calculated for $C_{20}H_{20}N_3O_4$ 366.1454; found 366.1447. **Melting point** 137-140 °C. **R$_f$** = 0.25 (15% EtOAc/cHex).

Ethyl 3-(benzo[*d*][1,3]dioxol-5-yl)-5-(methylamino)-1-phenyl-1*H*-pyrazole-4-carboxylate (5b')

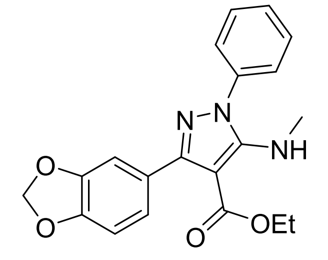

Chemical Formula: $C_{20}H_{19}N_3O_4$
Exact Mass: 365.1376

Appearance: yellow oil. Yield = 32 mg, 0.09 mmol, 49%. **^{1}H NMR (500 MHz, CDCl$_3$) δ/ppm** 7.60 (d, *J* = 7.9 Hz, 2H), 7.47 (t, *J* = 7.7 Hz, 2H), 7.38 (t, *J* = 7.5 Hz, 1H), 7.18-7.16 (m, 2H), 6.82 (d, *J* = 7.9 Hz, 1H), 6.56-6.61 (m, NH), 5.97 (s, 2H), 4.22 (q, *J* = 7.2 Hz, 2H), 2.57 (d, *J* = 5.5 Hz, 3H), 1.22 (t, *J* = 7.2 Hz, 3H). **^{13}C NMR (125 MHz, CDCl$_3$) δ/ppm** 165.4 (C), 154.5 (C), 152.2 (C), 147.6 (C), 146.8 (C), 139.6 (C), 129.1 (2xCH), 128.1 (CH), 127.3 (CH), 125.4 (2xCH), 123.4 (CH), 110.3 (CH), 107.5 (CH), 101.0 (CH$_2$), 94.9 (C), 59.6 (CH$_2$), 27.1 (CH$_3$), 14.2 (CH$_3$). **IR (neat)** ν/cm^{-1} 3349 (w), 2901 (w), 1660 (s), 1572 (s), 1458 (s), 1249 (s), 1129 (m), 1038 (m), 935 (m), 789 (m). **HRMS (TOF-EI+)** *m/z* [M$^+$H$^+$] calculated for $C_{20}H_{20}N_3O_4$ 366.1454; found 366.1446. **R$_f$** = 0.25 (15% EtOAc/cHex).

Ethyl 3-(methylamino)-1-phenyl-5-(*p*-tolyl)-1*H*-pyrazole-4-carboxylate (5c)

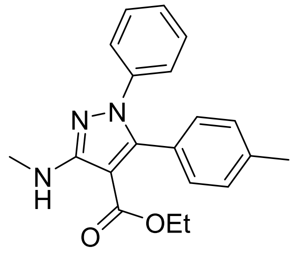

Chemical Formula: $C_{20}H_{21}N_3O_2$
Exact Mass: 335.1634

Appearance: white solid. Yield = 53 mg, 0.16 mmol, 48%. **^{1}H NMR (500 MHz, CDCl$_3$) δ/ppm** 7.24-7.15 (m, 5H), 7.13-7.08 (m, 4H), 5.58-5.63 (m, NH), 4.11 (q, *J* = 7.2 Hz, 2H), 3.05 (d, *J* = 5.2 Hz, 3H), 2.35 (s, 3H), 1.08 (t, *J* = 7.2 Hz, 3H). **^{13}C NMR (125 MHz, CDCl$_3$) δ/ppm** 164.9 (C), 158.6 (C), 145.5 (C), 139.6 (C), 138.7 (C), 130.3 (2xCH), 128.6 (2xCH), 128.4 (2xCH), 127.1 (C), 126.9 (CH), 125.2 (2xCH), 98.8 (C), 59.5 (CH$_2$), 29.7 (CH$_3$), 21.4 (CH$_3$), 14.0 (CH$_3$). **IR (neat)** ν/cm^{-1} 3345 (w), 2924 (m), 1662 (s), 1544 (m), 1411 (m), 1255 (m), 1135 (s), 824 (m), 788 (m). **HRMS (TOF-EI+)** *m/z* [M$^+$H$^+$] calculated for $C_{20}H_{22}N_3O_2$ 336.1712; found 336.1704. **Melting point** 143-145 °C. **R$_f$** = 0.35 (10% EtOAc/cHex).

Ethyl 5-(methylamino)-1-phenyl-3-(*p*-tolyl)-1*H*-pyrazole-4-carboxylate (5c')

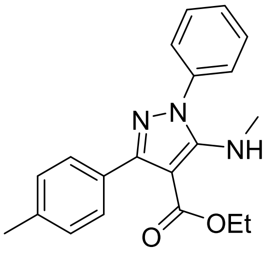

Chemical Formula: $C_{20}H_{21}N_3O_2$
Exact Mass: 335.1634

Appearance: yellow solid. Yield = 53 mg, 0.16 mmol, 48%. **^{1}H NMR (400 MHz, $CDCl_3$) δ/ppm** 7.61-7.58 (m, 2H), 7.54 (tt, *J* = 10.1, 2.4 Hz, 2H), 7.47-7.43 (m, 2H), 7.38-7.34 (m, 1H), 7.16 (d, *J* = 10.1 Hz, 2H), 6.55-6.61 (m, NH), 4.20 (t, *J* = 8.9 Hz, 2H), 2.57 (d, *J* = 7.1 Hz, 3H), 2.36 (s, 3H), 1.19 (t, *J* = 8.9 Hz, 3H). **^{13}C NMR (101 MHz, $CDCl_3$) δ/ppm** 165.6 (C), 154.7 (C), 152.8 (C), 139.8 (C), 138.0 (C), 130.6 (CH), 129.4 (2xCH), 129.1 (2xCH), 128.2 (2xCH), 128.1 (CH), 125.5 (2xCH), 95.0 (C), 59.7 (CH_2), 32.6 (CH_3), 21.4 (CH_3), 14.2 (CH_3). **IR (neat)** v/cm^{-1} 3338 (m), 2922 (w), 1662 (s), 1518 (s), 1408 (m), 1132 (m), 1085 (m), 827 (m), 776 (m). **HRMS (TOF-EI+)** *m/z* [M^+H^+] calculated for $C_{20}H_{22}N_3O_2$ 336.1712; found 336.1707. **Melting point** 117-119 °C. **R_f** = 0.35 (10% EtOAc/cHex).

Ethyl 5-(4-bromophenyl)-3-(methylamino)-1-phenyl-1*H*-pyrazole-4-carboxylate (5d)

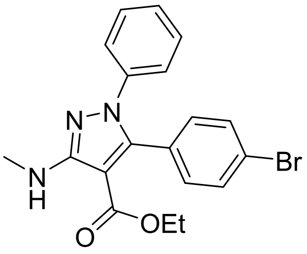

Chemical Formula: $C_{19}H_{18}BrN_3O_2$
Exact Mass: 399.0582

Appearance: yellow solid. Yield = 70 mg, 0.18 mmol, 47%. **^{1}H NMR (500 MHz, $CDCl_3$) δ/ppm** 7.44 (dt, *J* = 9.0, 2.5 Hz, 2H), 7.28-7.17 (m, 5H), 7.12 (dt, *J* = 9.0, 2.5 Hz, 2H), 5.58-5.64 (m, NH), 4.13 (q, *J* = 7.2 Hz, 2H), 3.06 (d, *J* = 5.2 Hz, 3H), 1.10 (t, *J* = 7.2 Hz, 3H). **^{13}C NMR (125 MHz, $CDCl_3$) δ/ppm** 164.6 (C), 158.6 (C), 144.0 (C), 139.2 (C), 132.1 (2xCH), 131.0 (2xCH), 129.1 (C), 128.9 (2xCH), 127.3 (2xCH), 125.25 (C), 123.2 (CH), 98.9 (C), 59.7 (CH_2), 29.68 (d, *J* = 5.0 Hz, CH_3), 14.1 (CH_3). **IR (neat)** v/cm^{-1} 3350 (m), 2864 (w), 1666 (s), 1579 (m), 1401 (m), 1241 (m), 1067 (m), 831 (s), 764 (m). **HRMS (TOF-EI+)** *m/z* [M^+H^+] calculated for $C_{19}H_{19}{}^{79}BrN_3O_2$ 400.0661; found 400.0653. **Melting point** 124-126 °C. **R_f** = 0.3 (15% EtOAc/cHex).

Ethyl 3-(4-bromophenyl)-5-(methylamino)-1-phenyl-1*H*-pyrazole-4-carboxylate (5d')

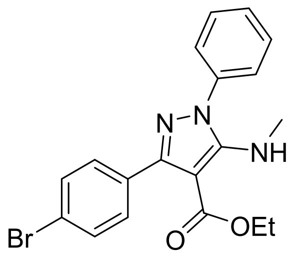

Chemical Formula: $C_{19}H_{18}BrN_3O_2$
Exact Mass: 399.0582

Appearance: yellow solid. Yield = 70 mg, 0.18 mmol, 47%. **^{1}H NMR (500 MHz, $CDCl_3$) δ/ppm** 7.61-7.46 (m, 8H), 7.40 (tt, *J* = 6.8, 1.3 Hz, 1H), 6.58-6.64 (m, NH), 4.22 (q, *J* = 7.1 Hz, 2H), 2.58 (d, *J* = 5.0 Hz, 3H), 1.21 (t, *J* = 7.1 Hz, 3H). **^{13}C NMR (125 MHz, $CDCl_3$) δ/ppm** 165.2 (C), 154.6 (C), 151.5 (C), 139.5 (C), 132.4 (C), 131.1 (2xCH), 130.6 (2xCH), 129.2

(2xCH), 128.3 (2xCH), 125.5 (C), 122.5 (CH), 94.8 (C), 59.7 (CH_2), 32.46 (d, J = 13.8 Hz, CH_3), 14.1 (CH_3). **IR (neat)** ν/cm^{-1} 3391 (w), 2922 (m), 1676 (s), 1543 9s), 1422 (m), 1253 (m), 1133 (s), 1012 (m), 835 (m), 789 (m). **HRMS (TOF-EI+)** *m/z* [M^+H^+] calculated for $C_{19}H_{19}{}^{79}BrN_3O_2$ 400.0661; found 400.0658. **Melting point** 148-150 °C. $\mathbf{R_f}$ = 0.3 (15% EtOAc/cHex).

Ethyl 3-(methylamino)-1-phenyl-5-(4-(trifluoromethyl)phenyl)-1*H*-pyrazole-4-carboxylate (5e)

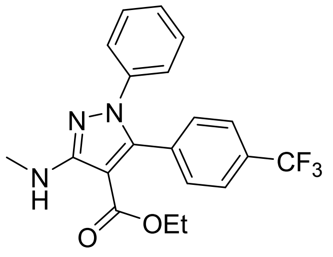

Chemical Formula: $C_{20}H_{18}F_3N_3O_2$
Exact Mass: 389.1351

Appearance: yellow solid. Yield = 74 mg, 0.19 mmol, 46%. **^{1}H NMR (400 MHz, $CDCl_3$) δ/ppm** 7.55 (d, J = 8.1 Hz, 2H), 7.36 (d, J = 8.1 Hz, 2H), 7.26-7.14 (m, 5H), 5.58-5.63 (m, NH), 4.09 (q, J = 7.1 Hz, 2H), 3.05 (d, J = 5.0 Hz, 3H), 1.04 (t, J = 7.1 Hz, 3H). **^{13}C NMR (101 MHz, $CDCl_3$) δ/ppm** 164.5 (C), 158.6 (C), 143.5 (C), 139.0 (C), 133.9 (C), 130.9 (2xCH), 130.8 (q, $J_{C\text{-}F}$ = 30 Hz, C), 128.9 (2xCH), 127.4 (CH), 126.7 (q, $J_{C\text{-}F}$ = 244 Hz, CF_3) 125.2 (2xCH), 124.7 (q, $J_{C\text{-}F}$ = 4 Hz, 2xCH), 99.2 (C), 59.7 (CH_2), 29.7 (CH_3), 13.9 (CH_3). **^{19}F-NMR (376 MHz, $CDCl_3$) δ/ppm** -62.8. **IR (neat)** ν/cm^{-1} 3376 (w), 2901 (w), 1666 (m), 1575 (m), 1320 (s), 1246 (m), 1104 (s), 1062 (s), 848 (m), 771 (m). **HRMS (TOF-EI+)** *m/z* [M^+H^+] calculated for $C_{20}H_{19}F_3N_3O_2$ 390.1429; found 390.1423. **Melting point** 108-110 °C. $\mathbf{R_f}$ = 0.3 (15% EtOAc/cHex).

Ethyl 5-(methylamino)-1-phenyl-3-(4-(trifluoromethyl)phenyl)-1*H*-pyrazole-4-carboxylate (5e')

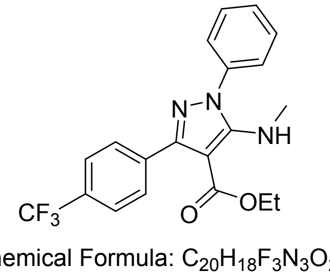

Chemical Formula: $C_{20}H_{18}F_3N_3O_2$
Exact Mass: 389.1351

Appearance: yellow solid. Yield = 74 mg, 0.19 mmol, 46%. **^{1}H NMR (400 MHz, $CDCl_3$) δ/ppm** 7.78 (d, J = 8.0 Hz, 2H), 7.61 (d, J = 7.6 Hz, 4H), 7.52-7.46 (m, 2H), 7.42-7.38 (m, 1H), 6.58-5.63 (m, NH), 4.21 (q, J = 7.2 Hz, 2H), 2.59 (d, J = 5.6 Hz, 3H), 1.18 (t, J = 7.2 Hz, 3H). **^{13}C NMR (101 MHz, $CDCl_3$) δ/ppm** 165.4 (C), 154.9 (C), 151.6 (C), 139.8 (C), 137.5 (C), 130.1 (q, $J_{C\text{-}F}$ = 33 Hz, C), 130.2 (2xCH), 129.5 (2xCH), 128.7 (CH), 128.5 (q, $J_{C\text{-}F}$ = 259 Hz, CF_3), 125.8 (2xCH), 124.7 (q, $J_{C\text{-}F}$ = 4 Hz, 2xCH), 95.2 (C), 60.1 (CH_2), 32.8 (CH_3), 14.4 (CH_3). **^{19}F-NMR (376 MHz, $CDCl_3$) δ/ppm** -62.6. **IR (neat)** ν/cm^{-1} 3407 (w), 2923 (w), 1673 (m), 1553 (m), 1324 (s), 1110 (s), 1064 (s), 849 (m), 780 (m). **HRMS (TOF-EI+)** *m/z* [M^+H^+] calculated for $C_{20}H_{19}F_3N_3O_2$ 390.1429; found 390.1424. **Melting point** 148-150 °C. $\mathbf{R_f}$ = 0.3 (15% EtOAc/cHex).

4. Hydrogenation Studies on crude Ketenimine 2a

To aid in confirming the structure of the ketenimine product a solution of crude product (ca. 10 mM, MeCN) from a fresh photolysis experiment was directly passed through a H-Cube Mini hydrogenation reactor equipped with a 10% Pd/C cartridge (rt, 10 bar, 1 mL/min) using the 'full hydrogen' mode of this instrument. Both ^{1}H-NMR spectra of the crude material, as well as a sample that was passed through a short plug of silica (cyclohexane as eluent, see below) indicated a new reaction product (see ^{1}H NMR below) that is consistent with the proposed enamine as a ca. 2:1 mixture of alkene isomers. This confirmed the generation of the proposed ketenimine species.

Schematic reaction set-up:

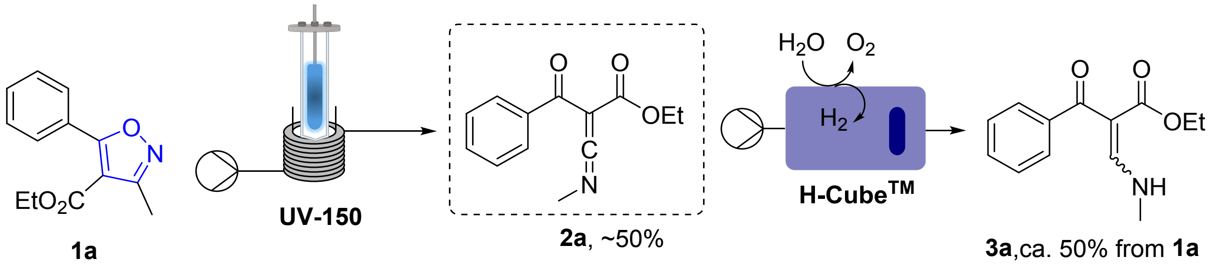

^{1}H NMR spectrum of enamine products (after SiO_2 plug):

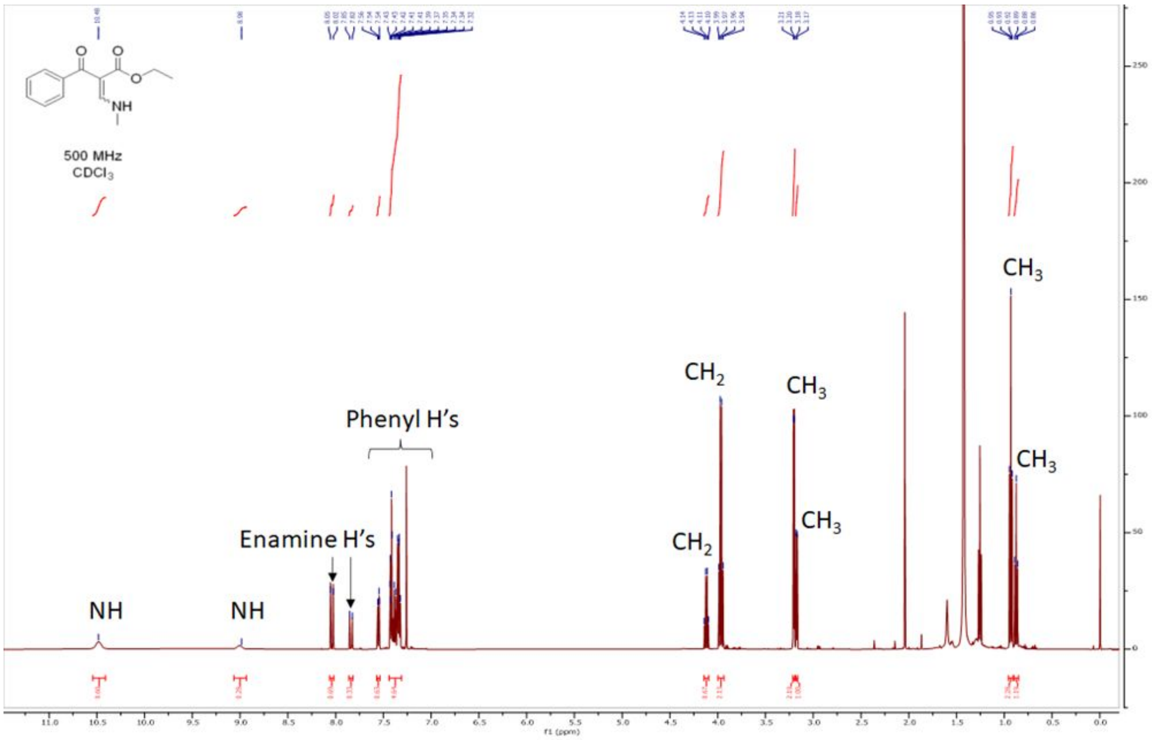

5. Exemplary IR Spectrum of Ketenimine 2j

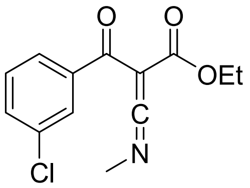

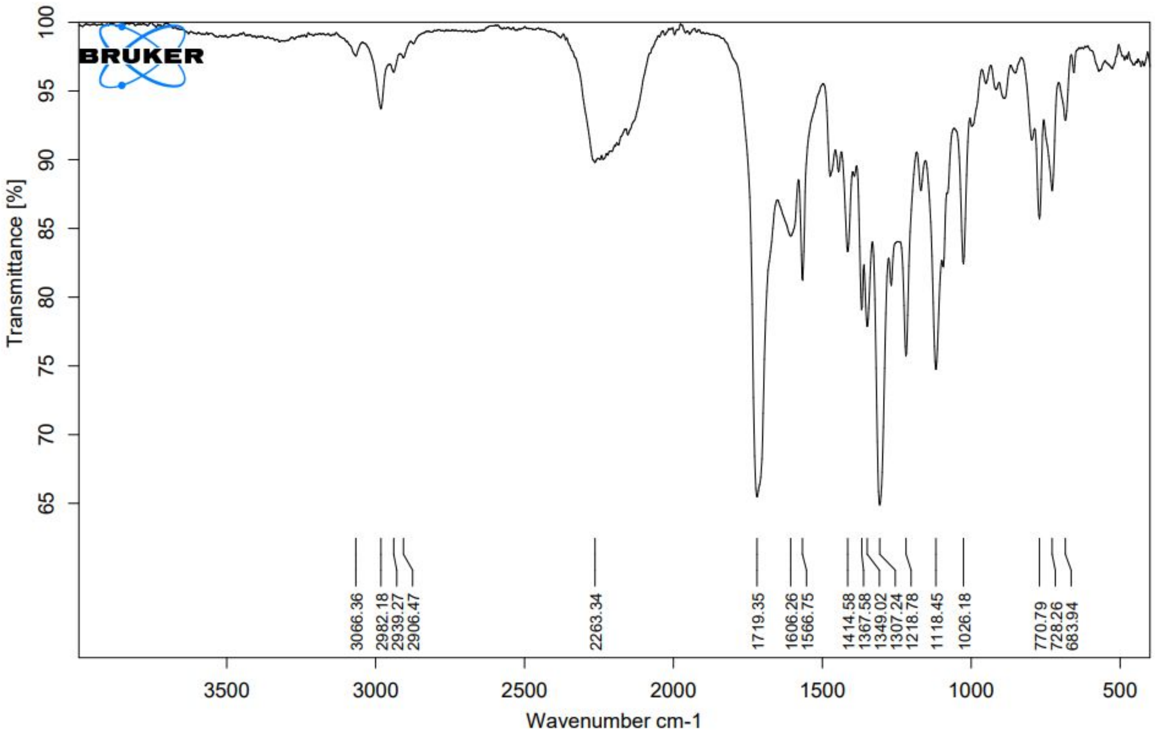

6.a Copies of NMR Spectra of Isoxazoles 1a-1k

Ethyl 3-methyl-5-phenylisoxazole-4-carboxylate (1a)

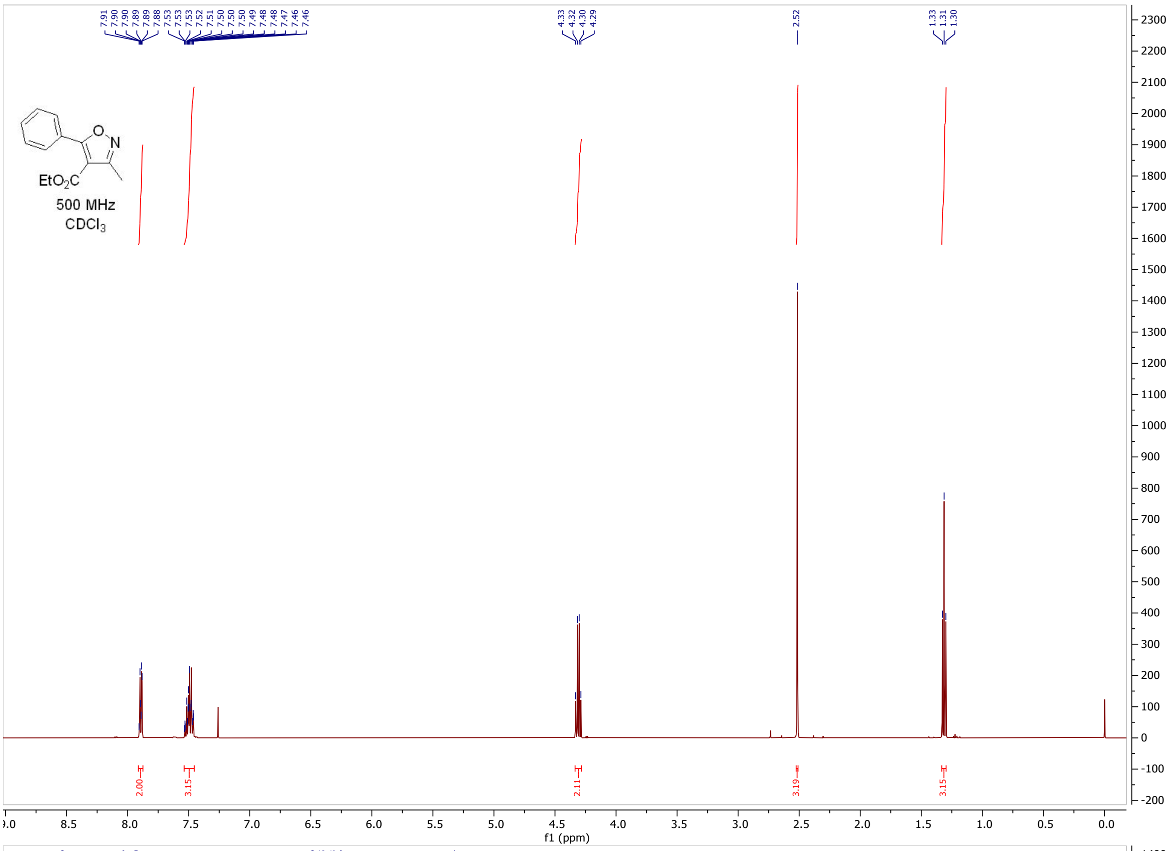

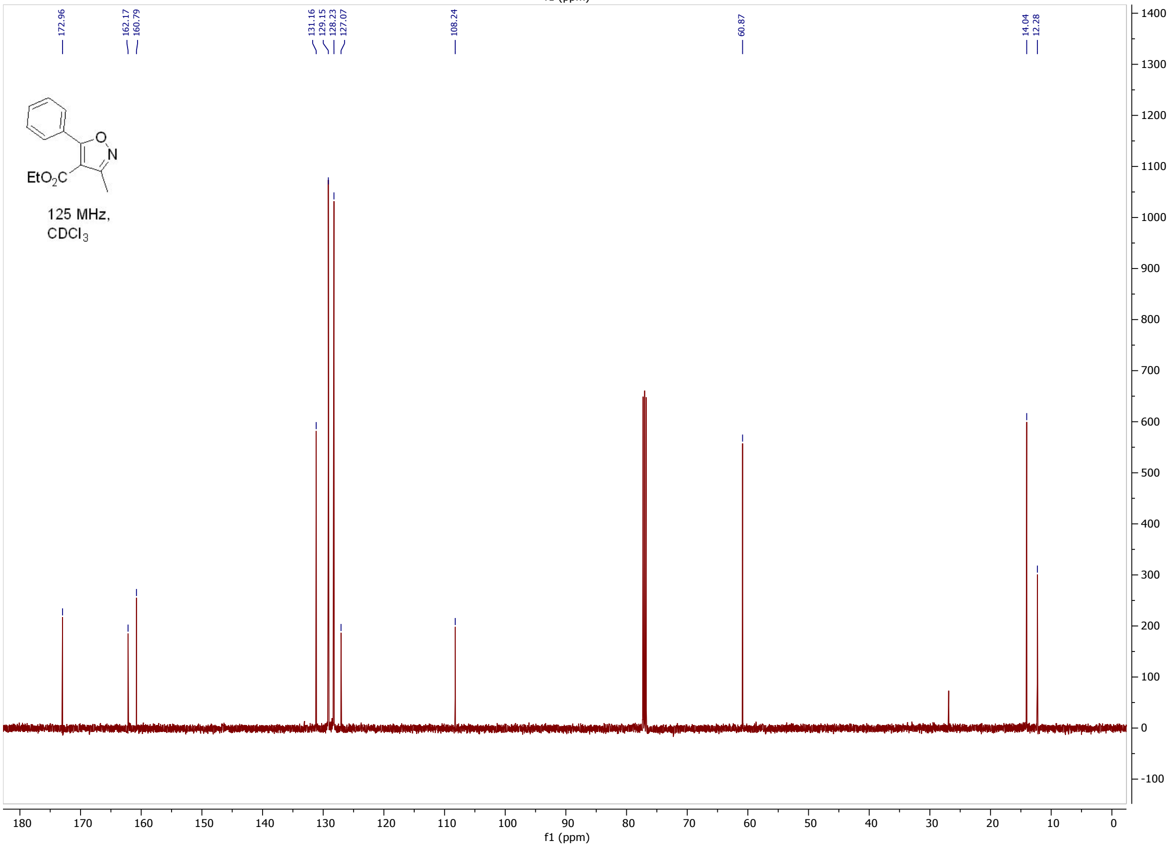

Ethyl 5-(benzo[*d*][1,3]dioxol-5-yl)-3-methylisoxazole-4-carboxylate (1b)

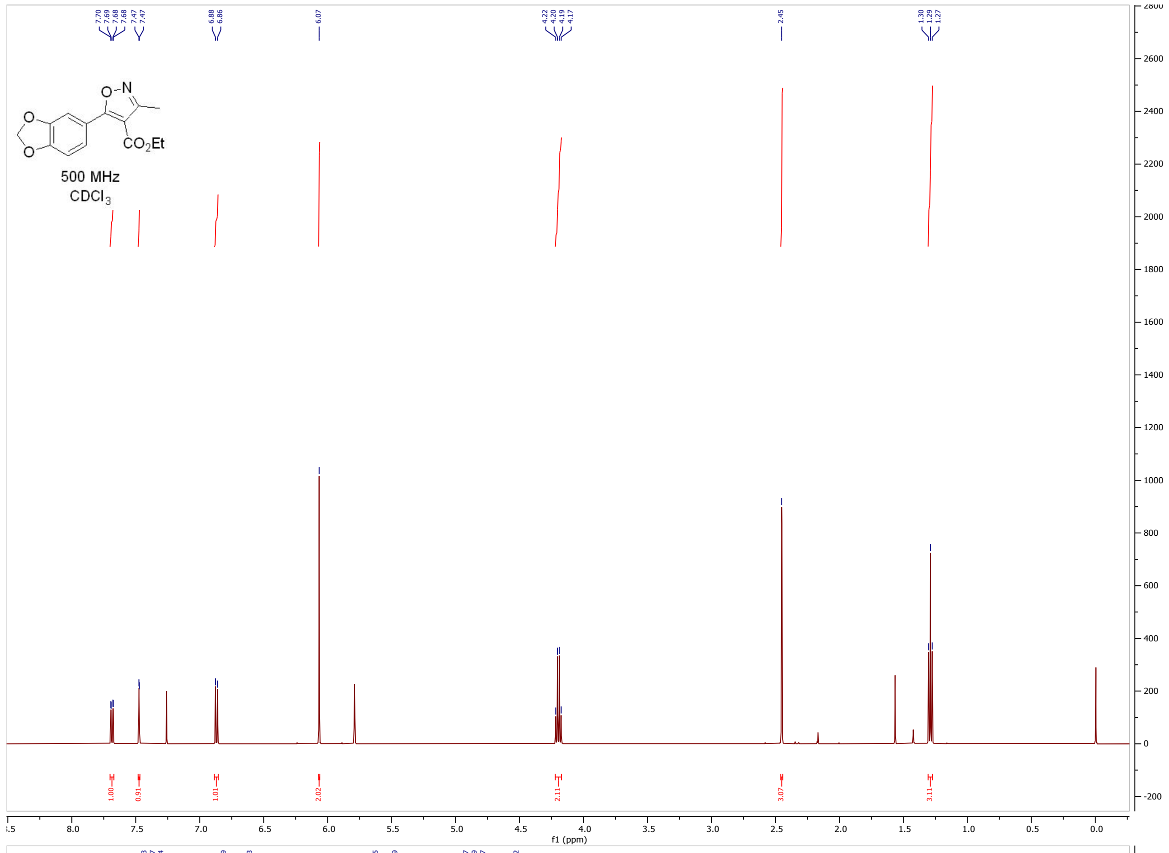

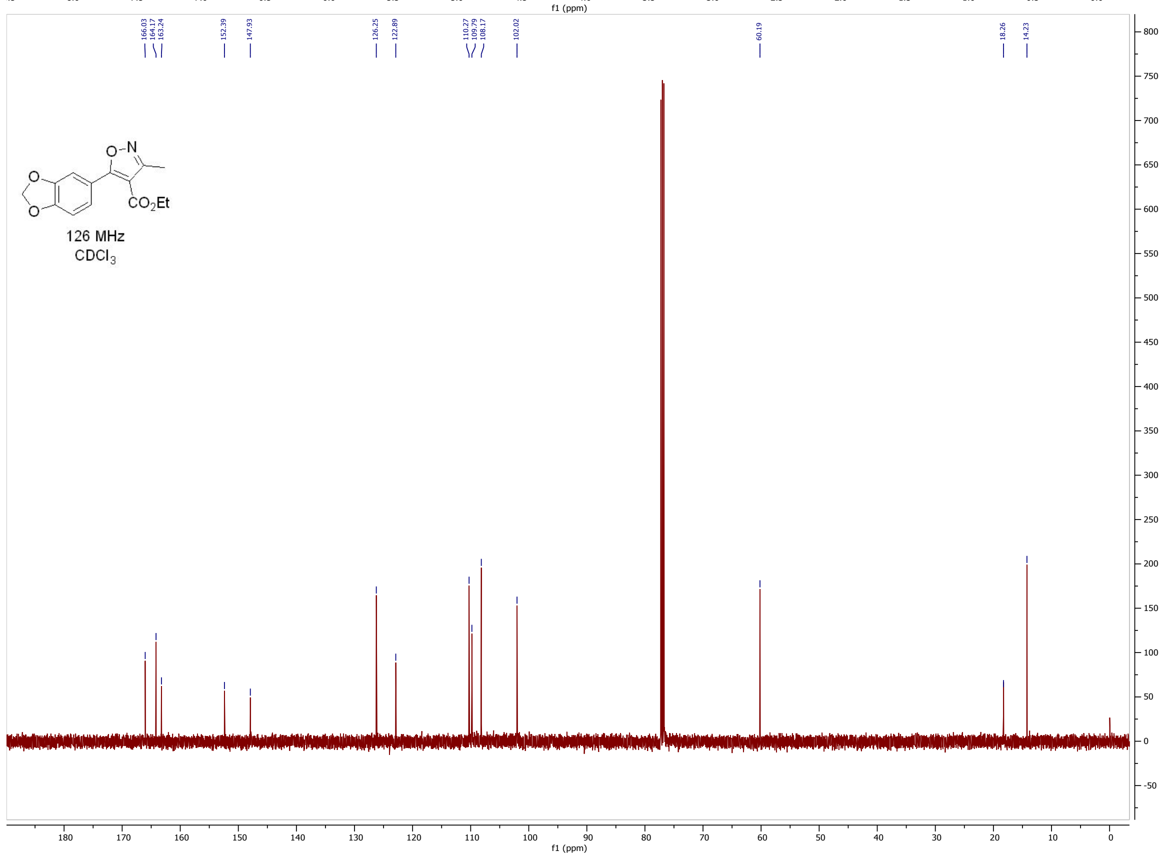

Ethyl 3-methyl-5-(p-tolyl)isoxazole-4-carboxylate (1c)

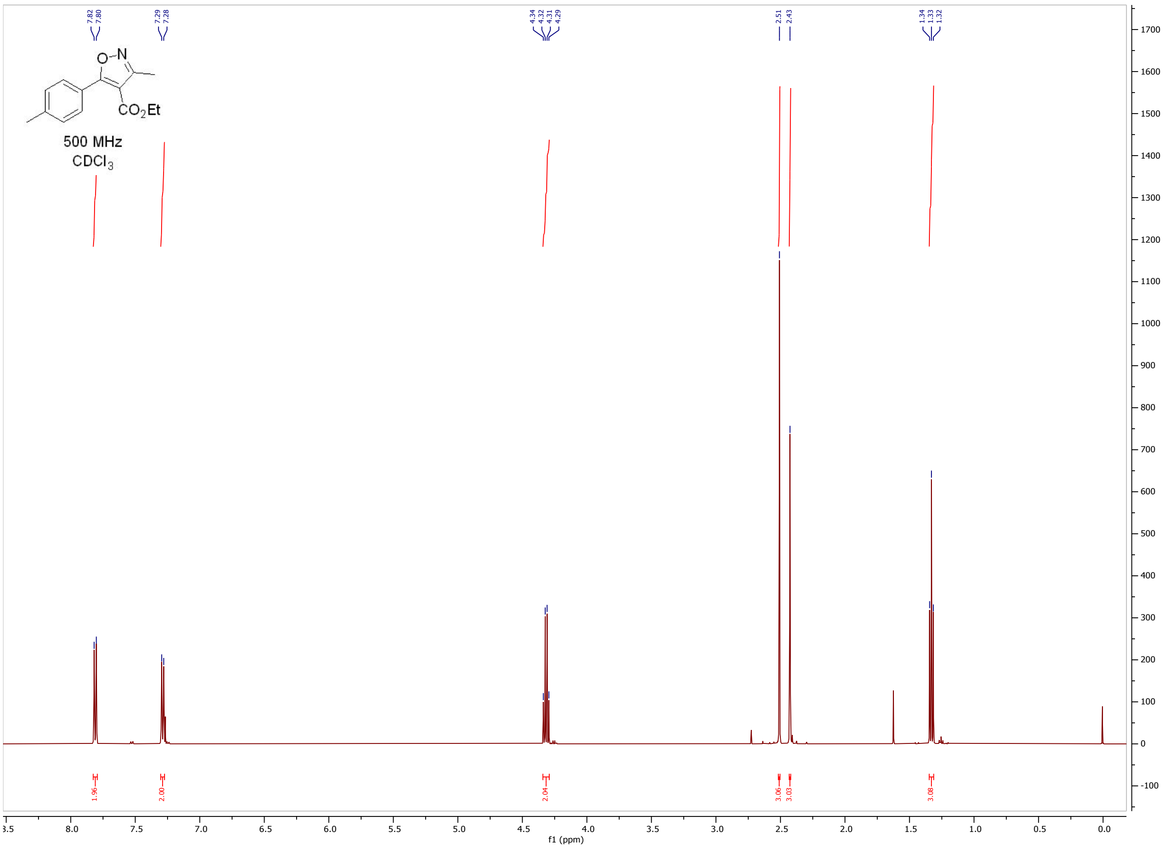

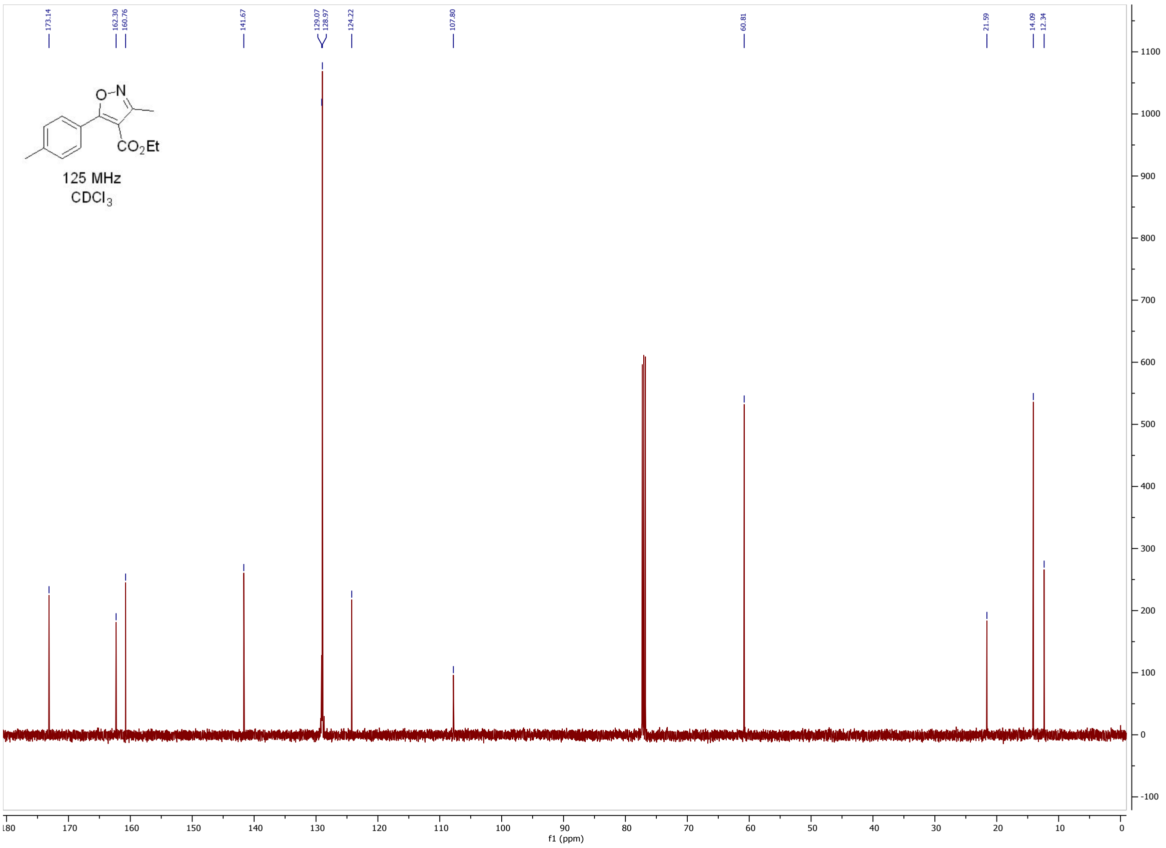

Ethyl 5-(4-bromophenyl)-3-methylisoxazole-4-carboxylate (1d)

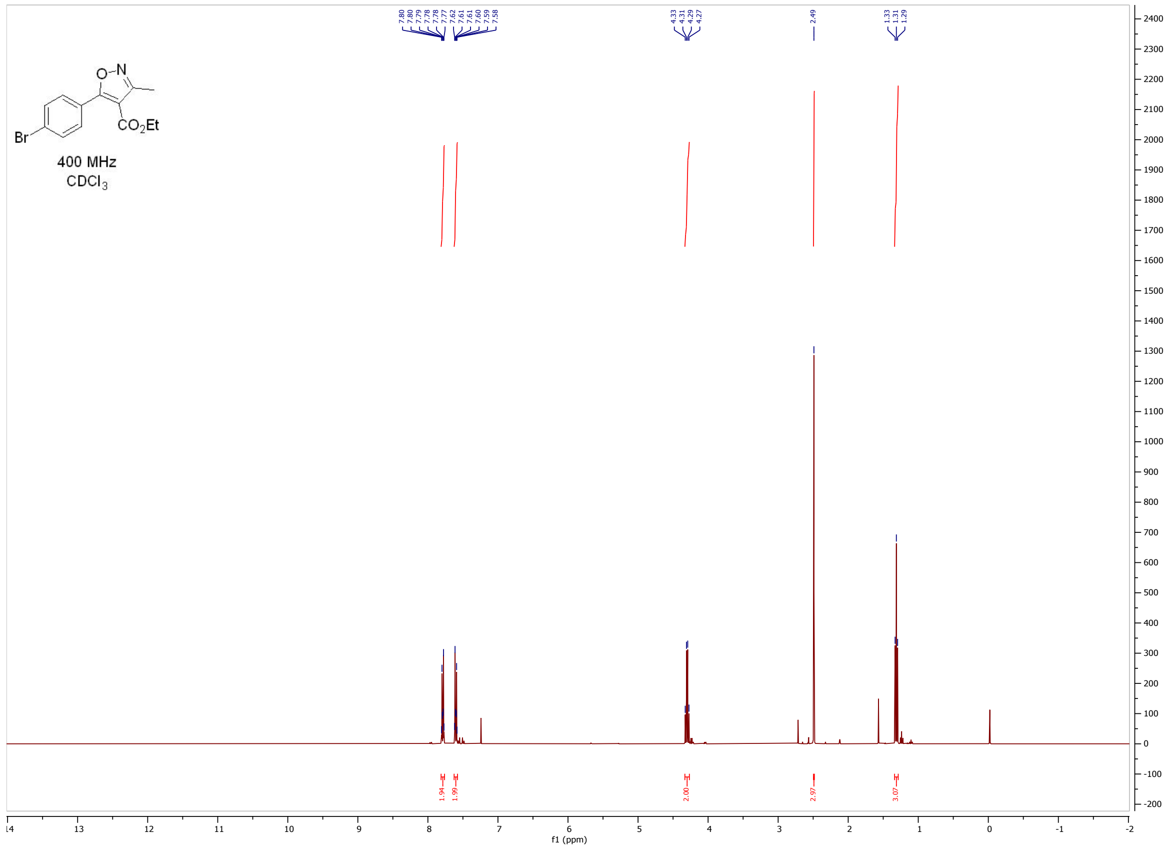

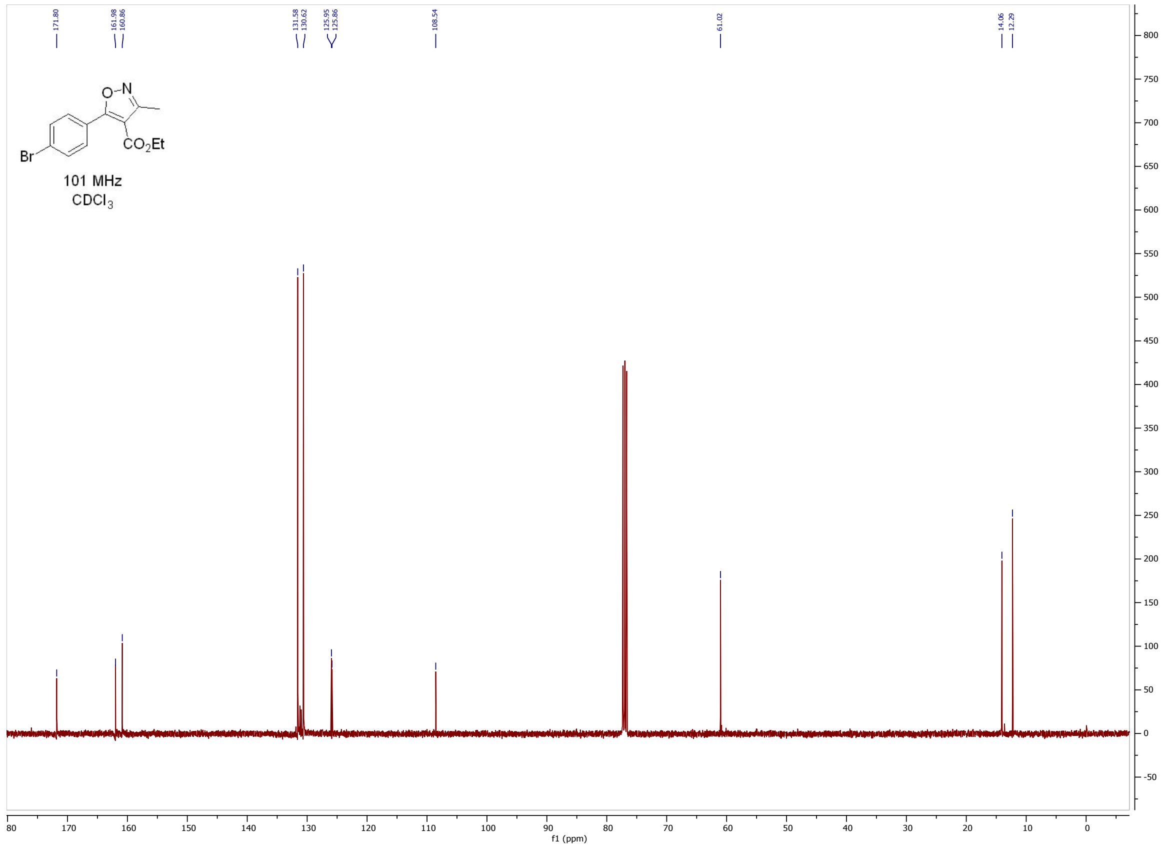

Ethyl 3-methyl-5-(4-(trifluoromethyl)phenyl)isoxazole-4-carboxylate (1e)

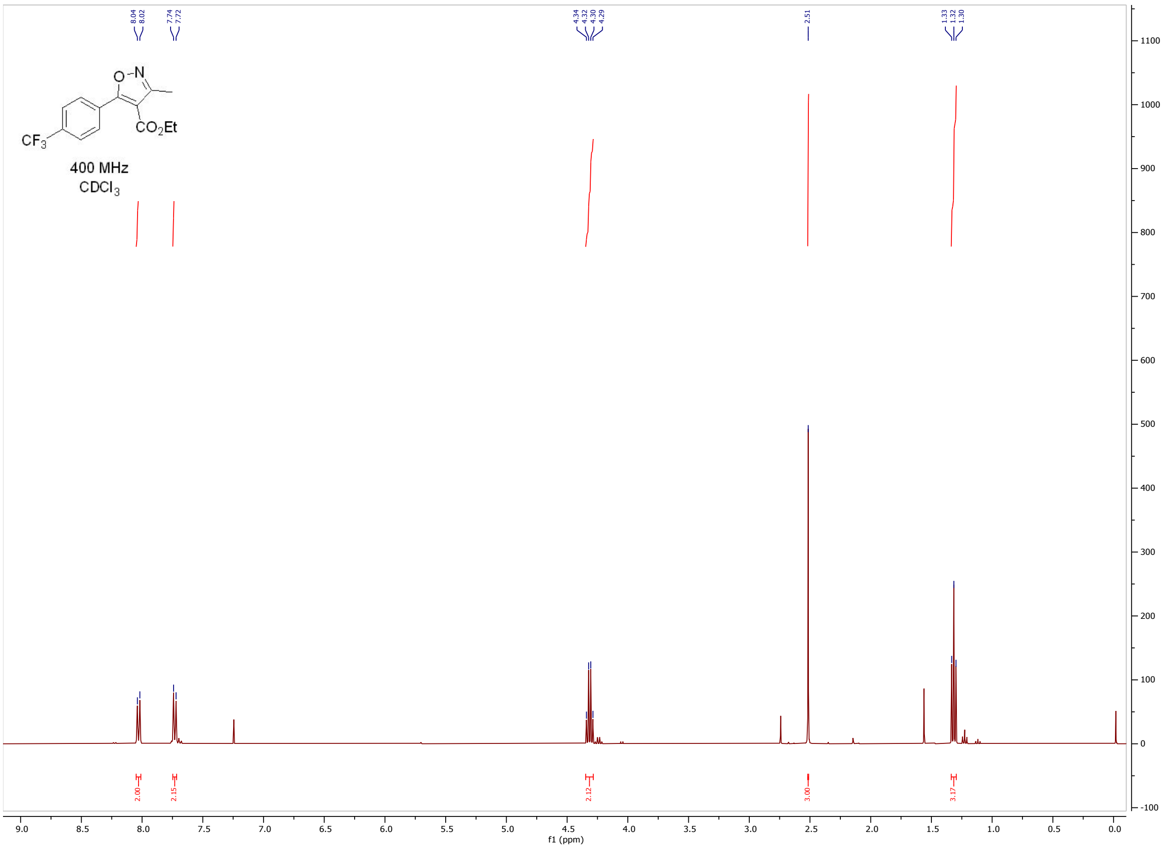

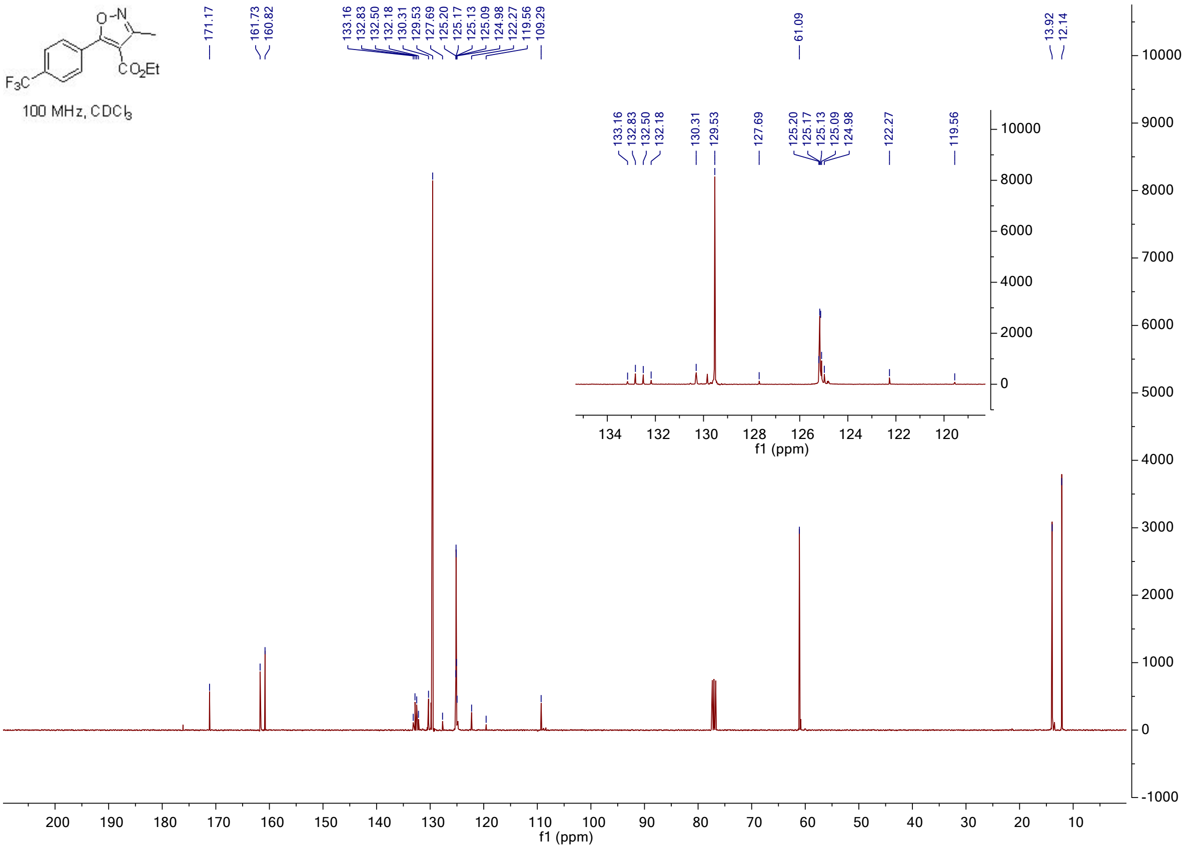

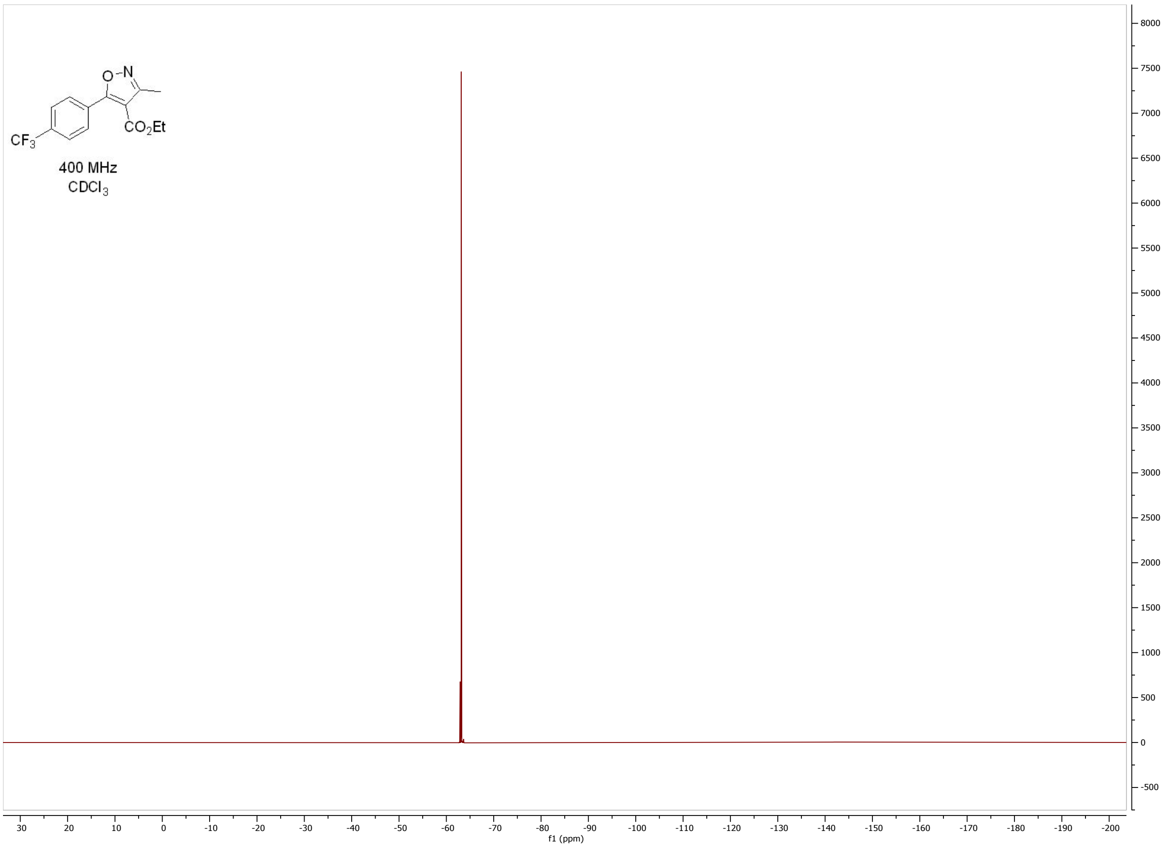
CF3
CO2Et
400 MHz
CDCl3
f1 (ppm)

Ethyl 5-(4-chloro-3-methylphenyl)-3-methylisoxazole-4-carboxylate (1f)

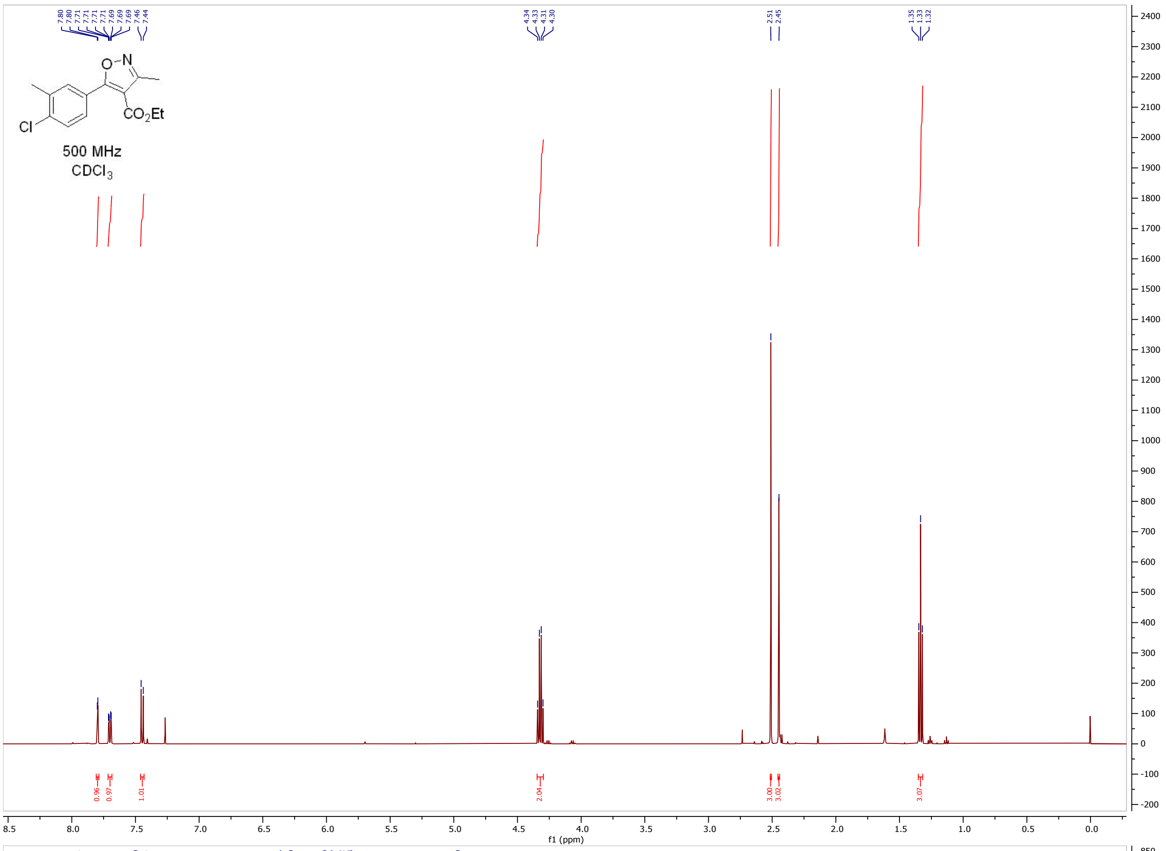

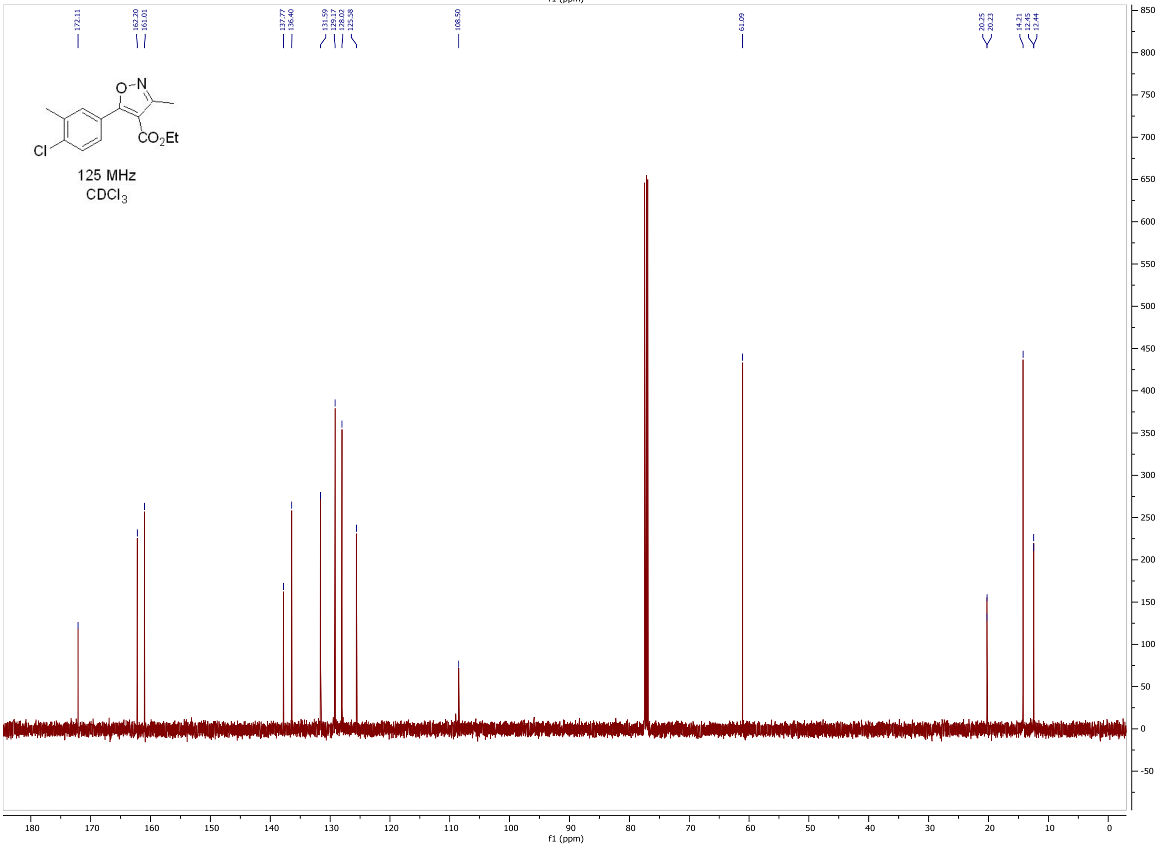

Ethyl 3-methyl-5-(4-(trifluoromethoxy)phenyl)isoxazole-4-carboxylate (1g)

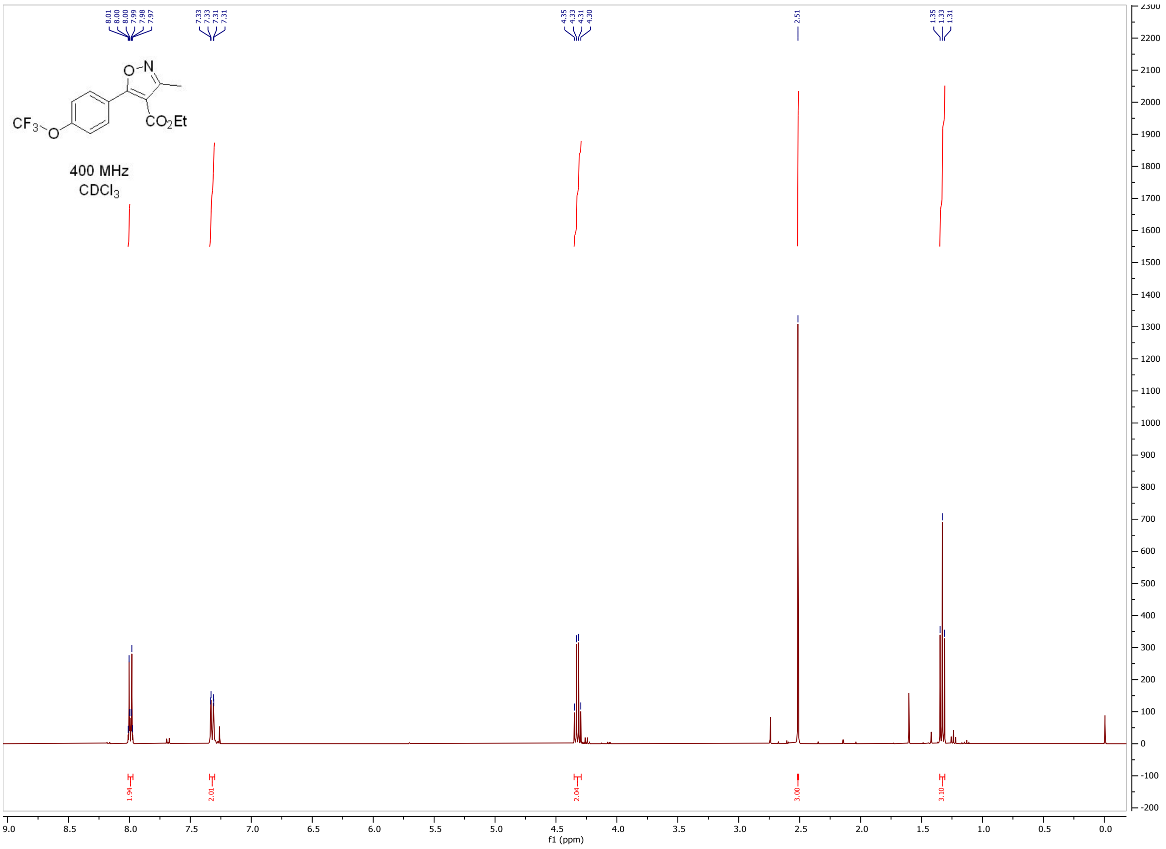

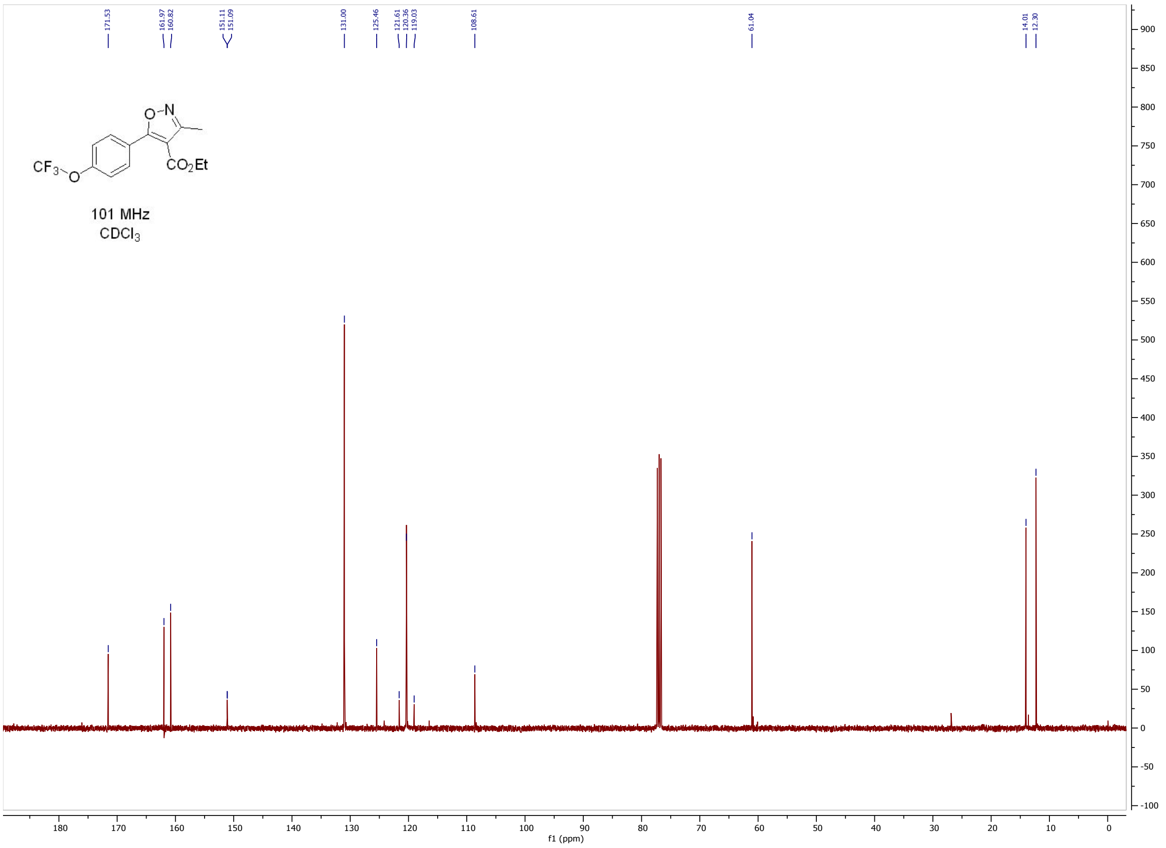

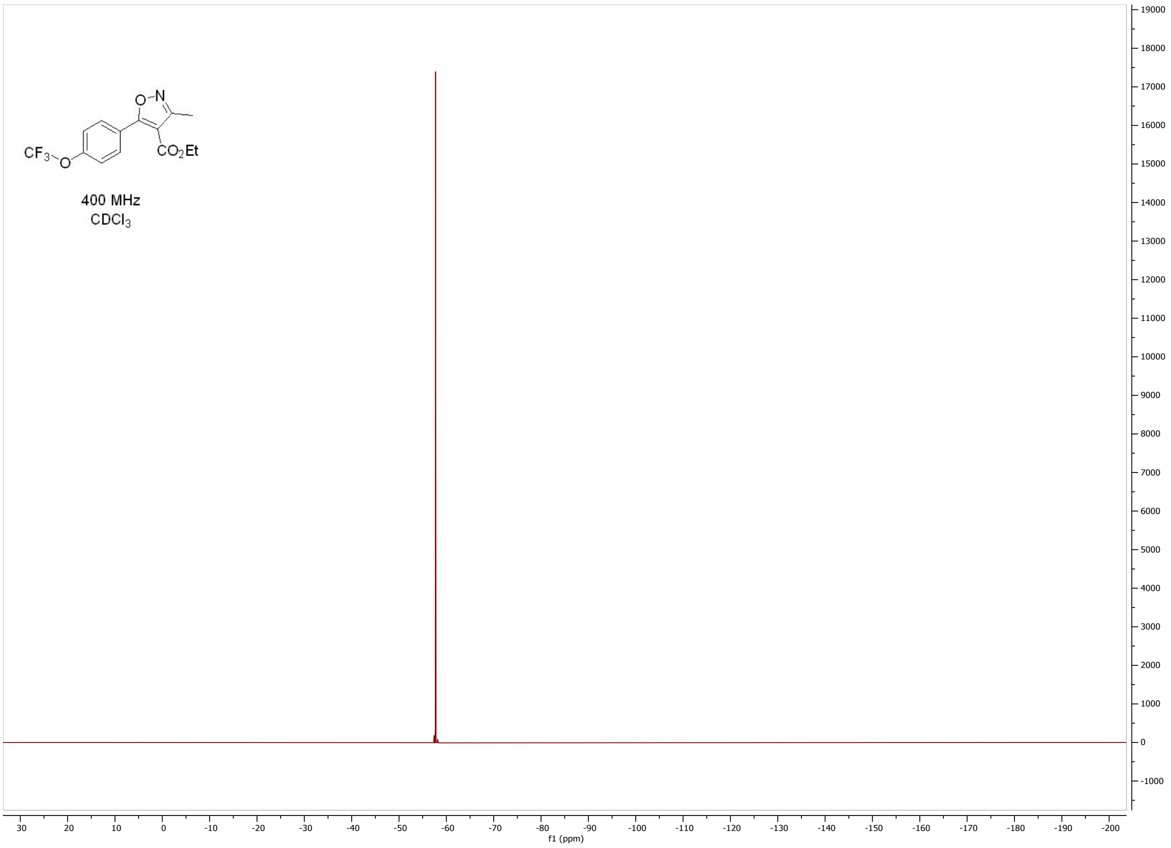

CF3 O
O N
CO2Et
400 MHz
CDCl3
19000
18000
17000
16000
15000
14000
13000
12000
11000
10000
9000
8000
7000
6000
5000
4000
3000
2000
1000
0
-1000
30 20 10 0 -10 -20 -30 -40 -50 -60 -70 -80 -90 -100 -110 -120 -130 -140 -150 -160 -170 -180 -190 -200
f1 (ppm)

Ethyl 5-(3,5-dichlorophenyl)-3-methylisoxazole-4-carboxylate (1h)

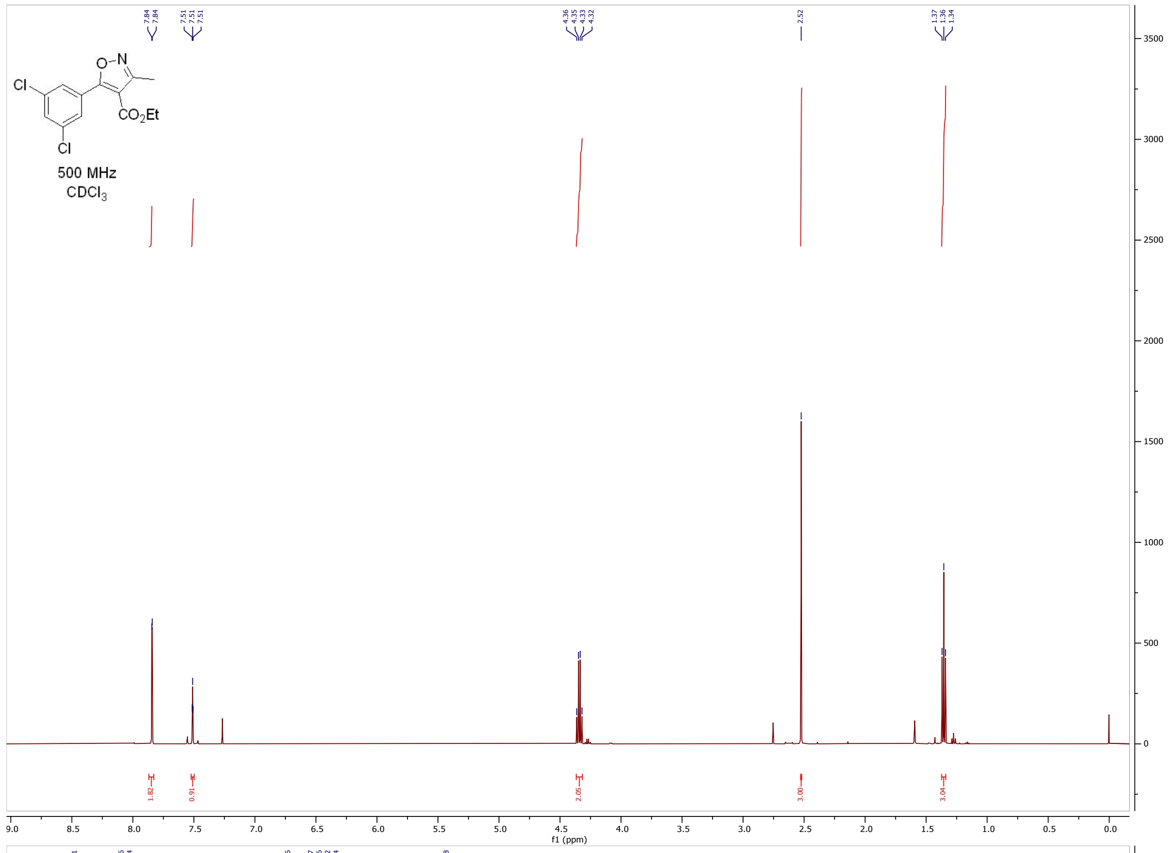

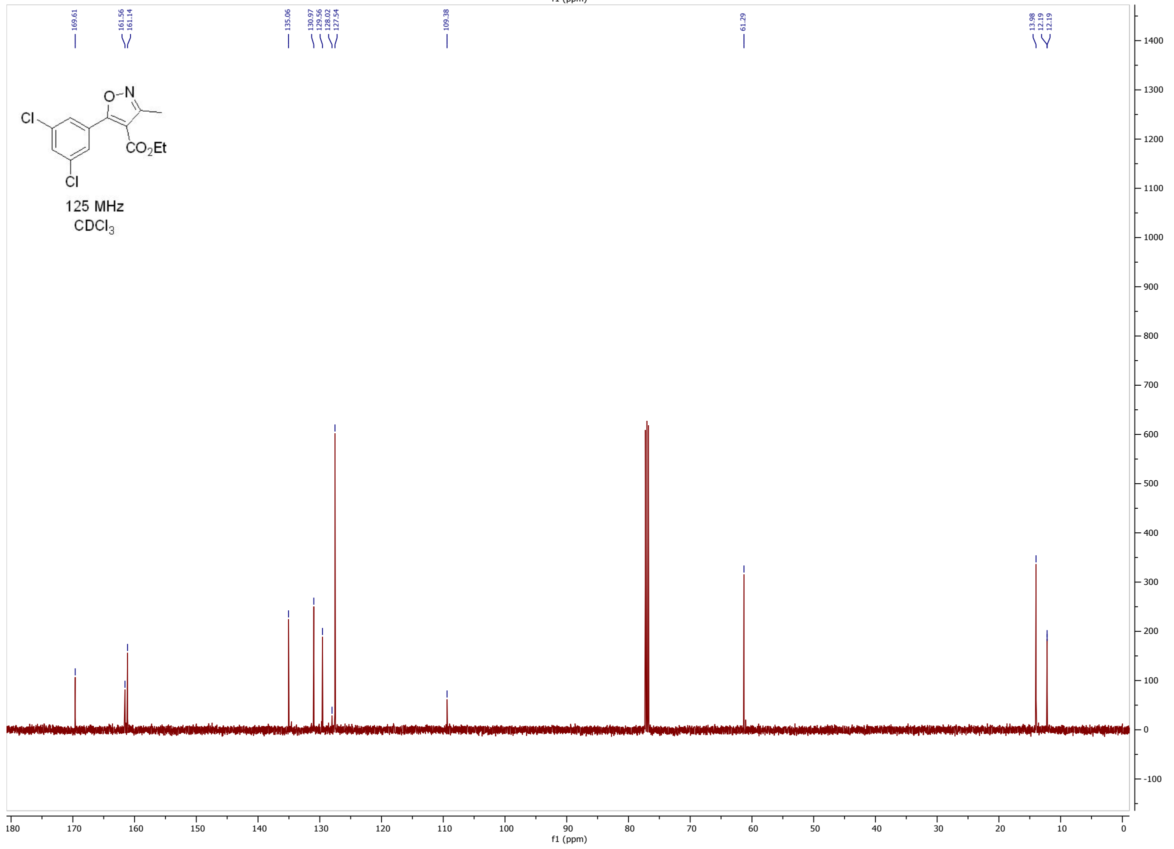

Ethyl 5-(2-fluorophenyl)-3-methylisoxazole-4-carboxylate (1i)

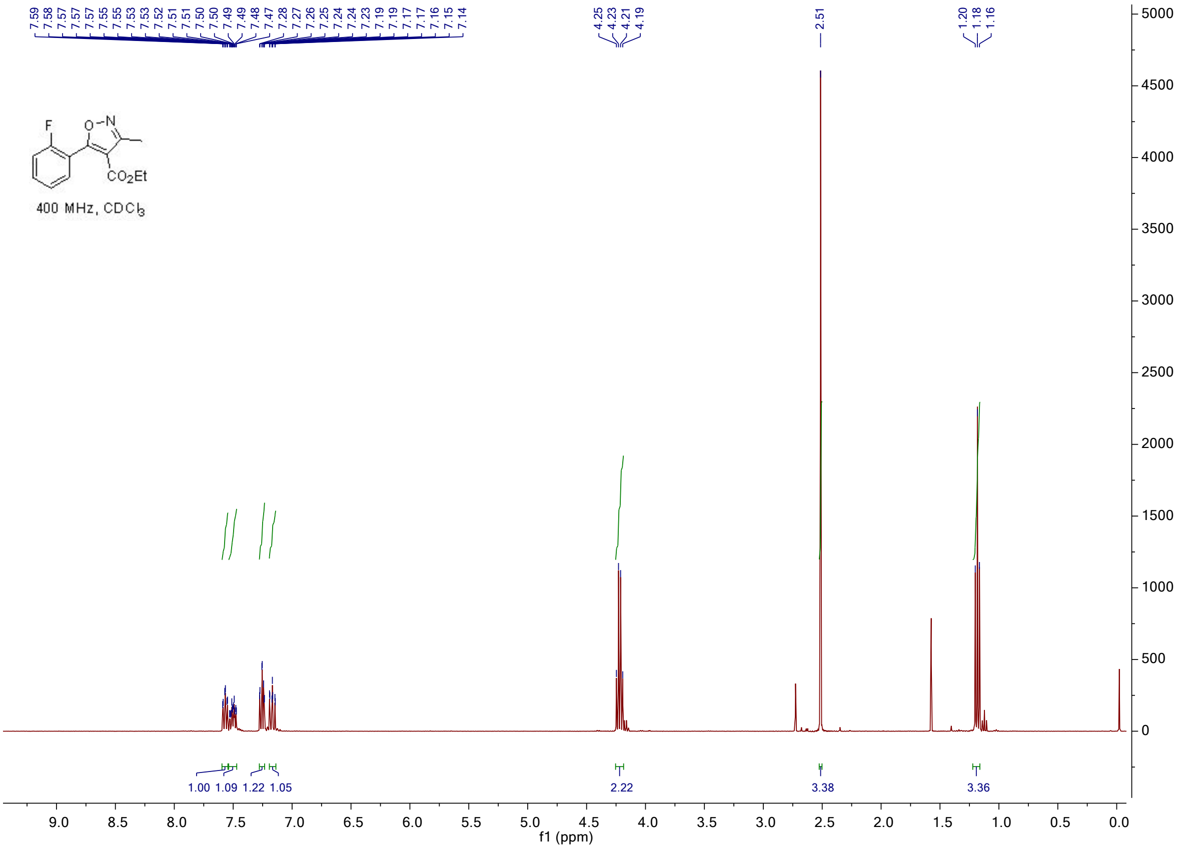

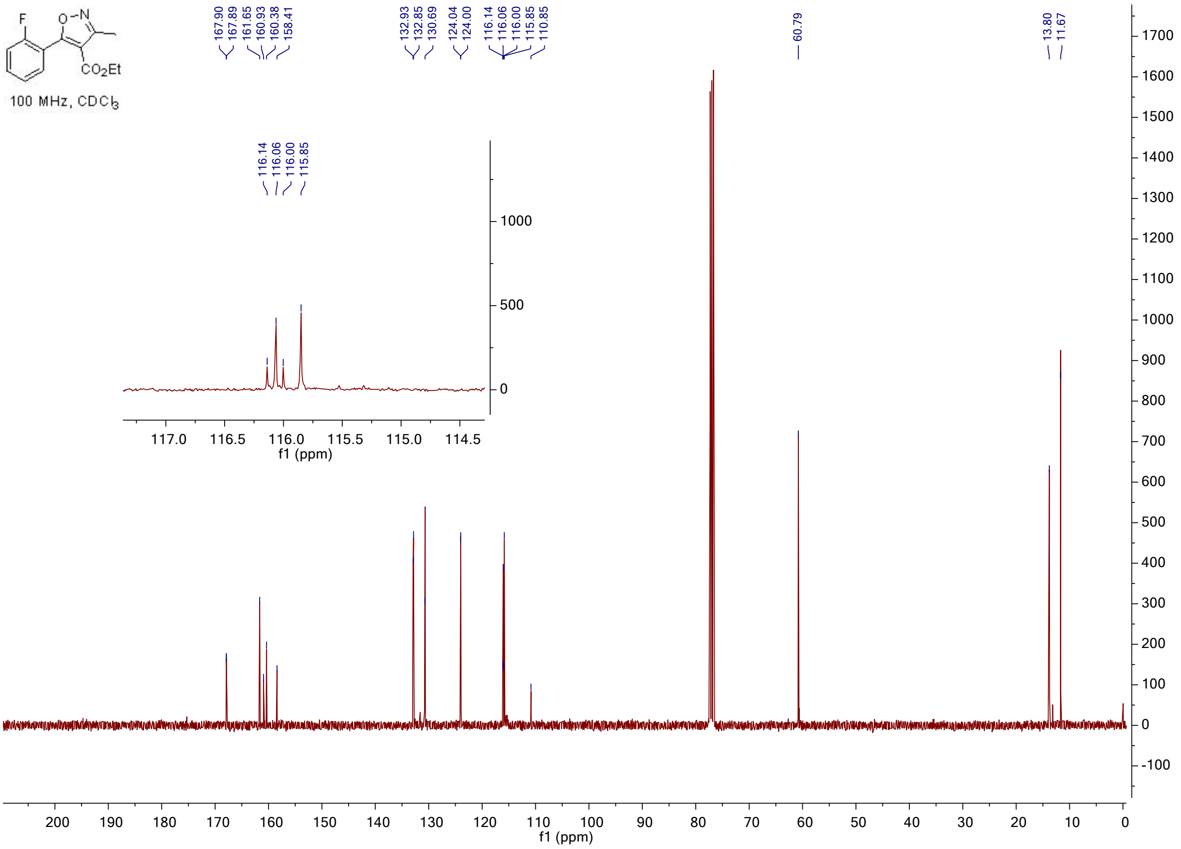

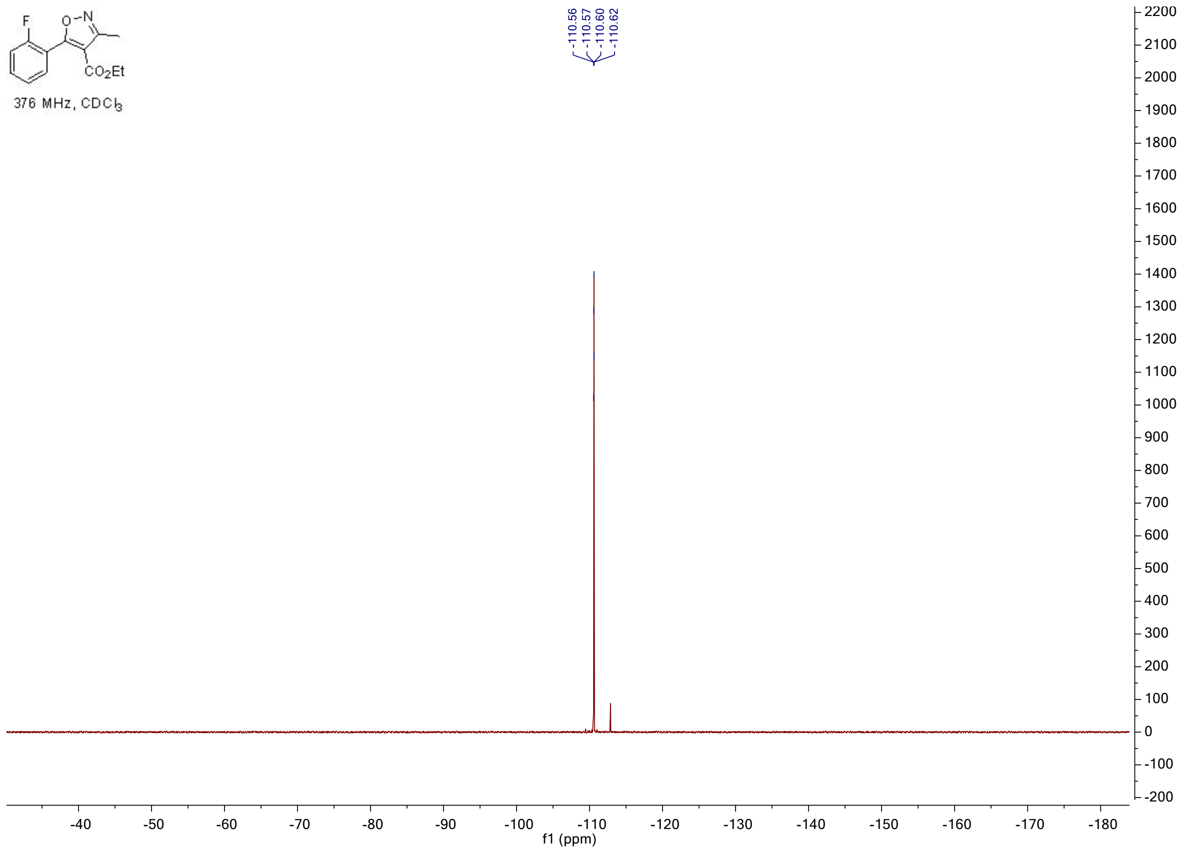

F
O
N
CO2Et
376 MHz, CDCl3
-110.56
-110.57
-110.60
-110.62
2200
2100
2000
1900
1800
1700
1600
1500
1400
1300
1200
1100
1000
900
800
700
600
500
400
300
200
100
0
-100
-200
-40
-50
-60
-70
-80
-90
-100
-110
-120
-130
-140
-150
-160
-170
-180
f1 (ppm)

Ethyl 5-(3-chlorophenyl)-3-methylisoxazole-4-carboxylate (1j)

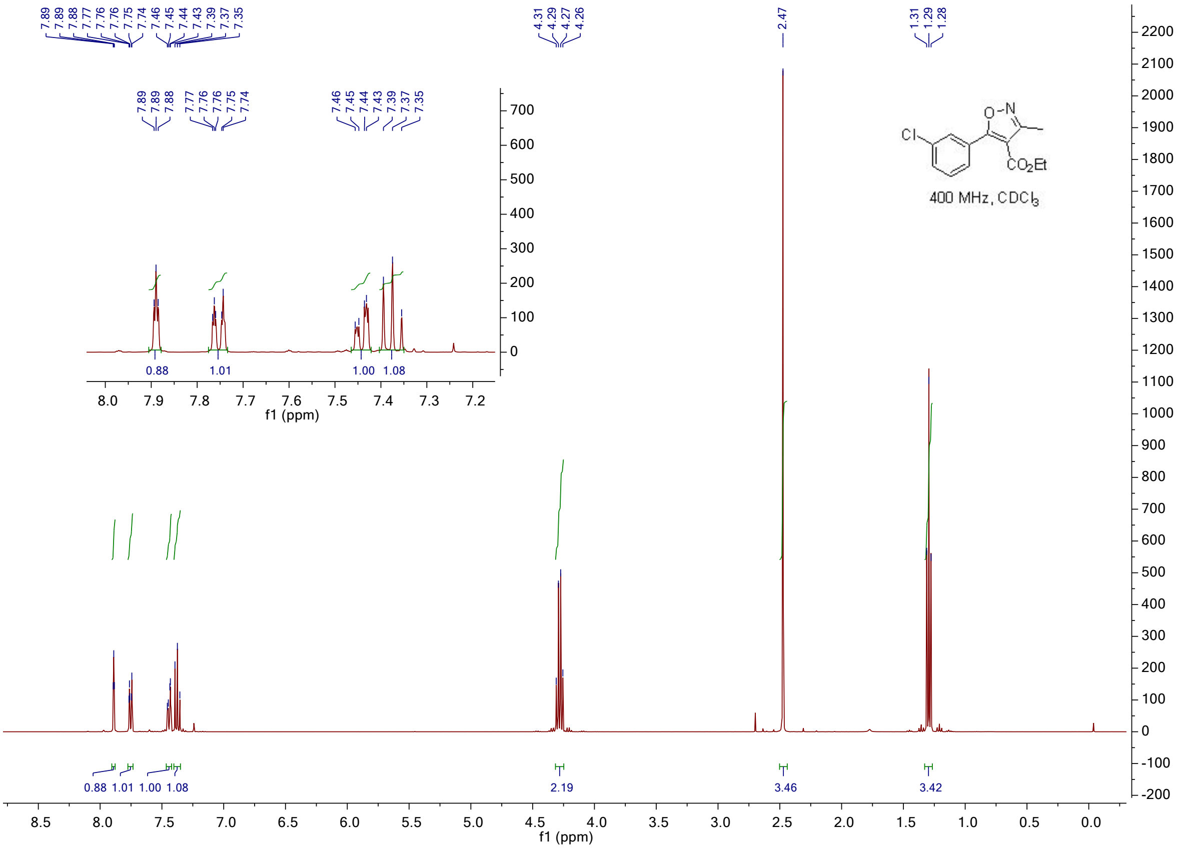

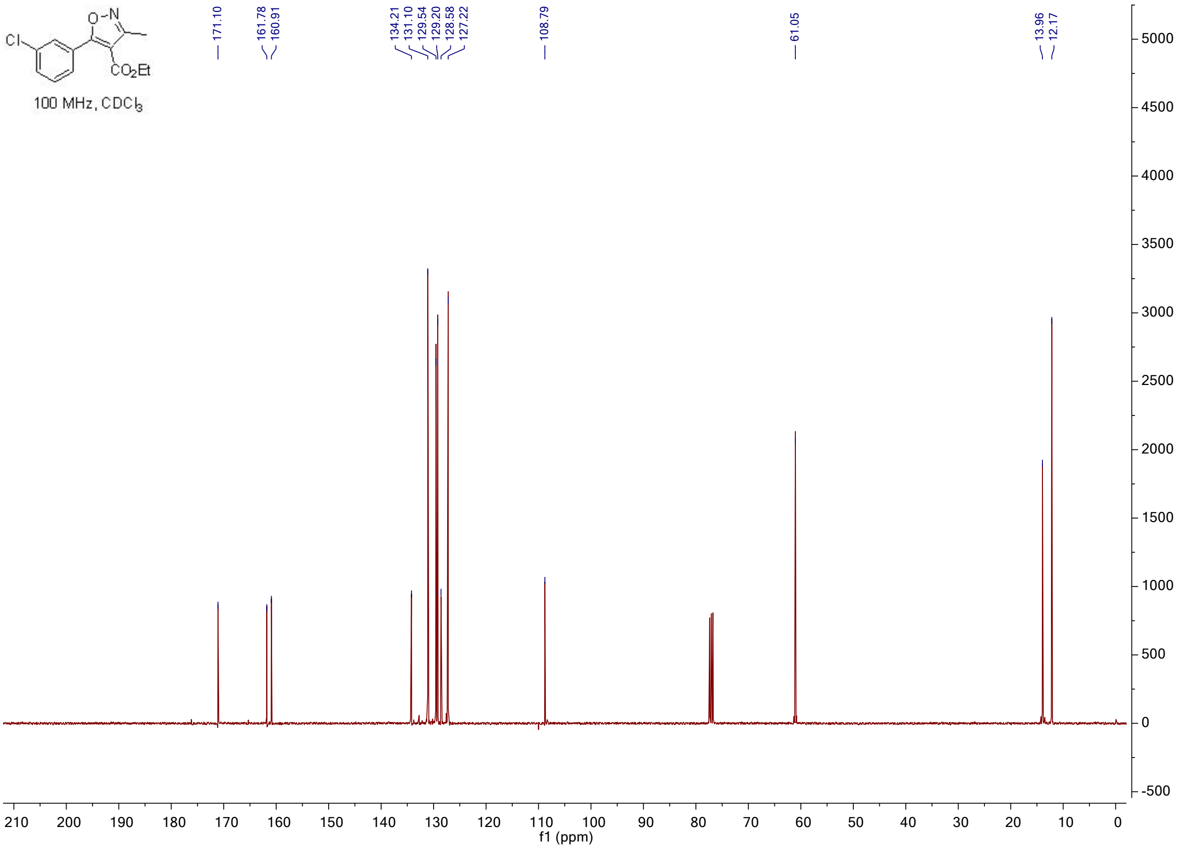

Ethyl 5-cyclohexyl-3-methylisoxazole-4-carboxylate (1k) – conformers present

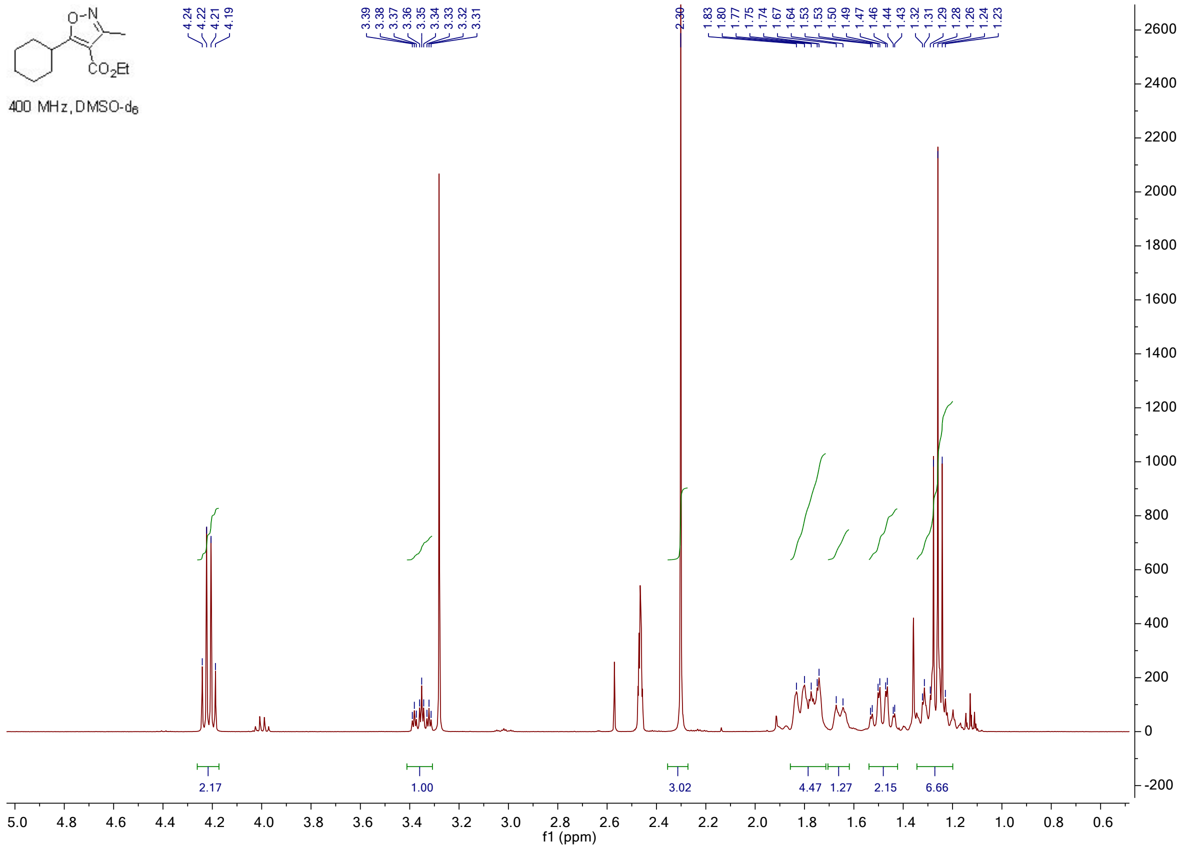

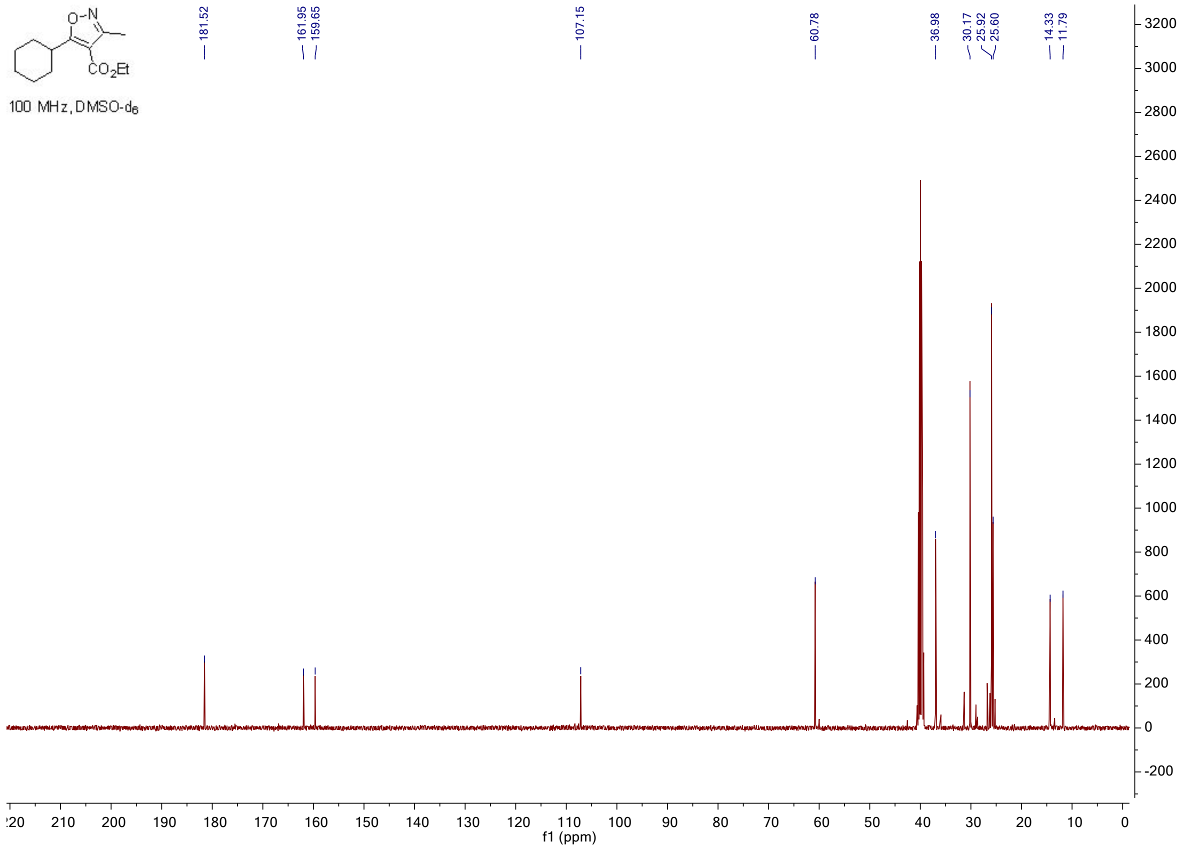

Ethyl (*E*)-3-methyl-5-styrylisoxazole-4-carboxylate (1l)

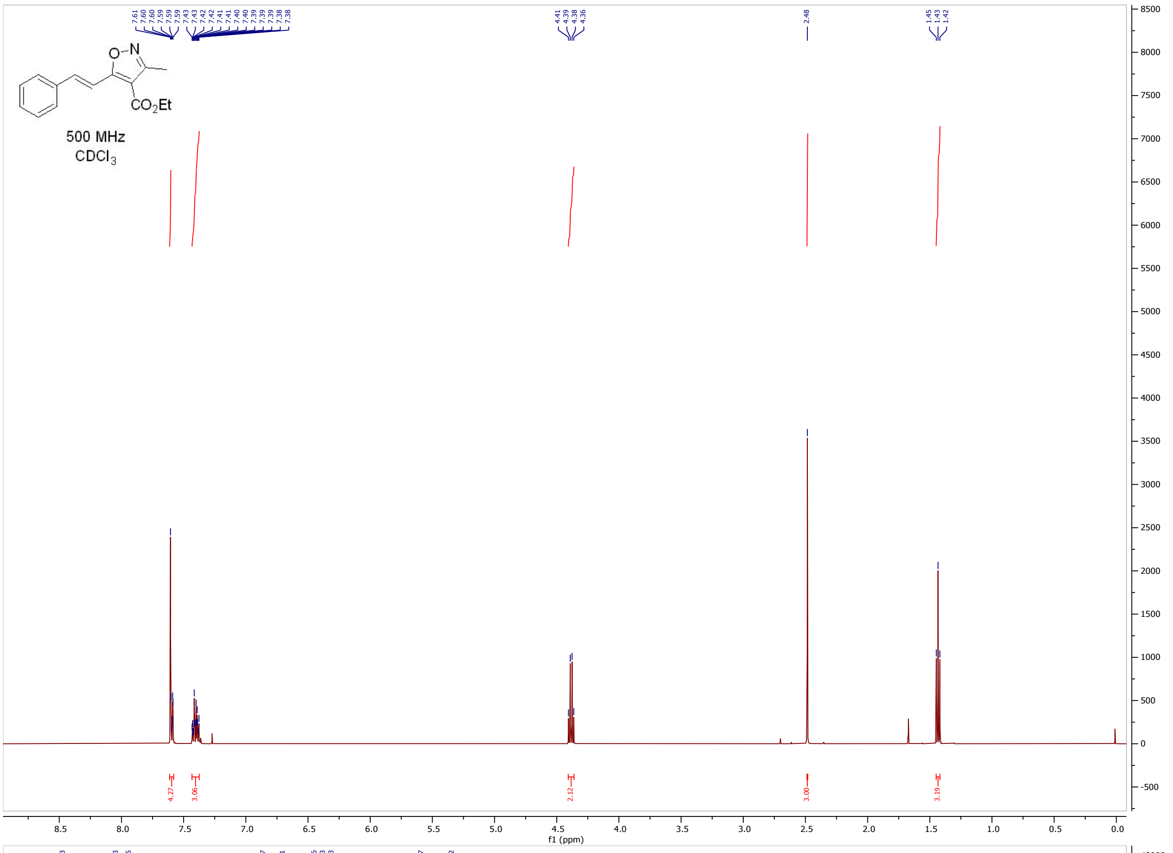

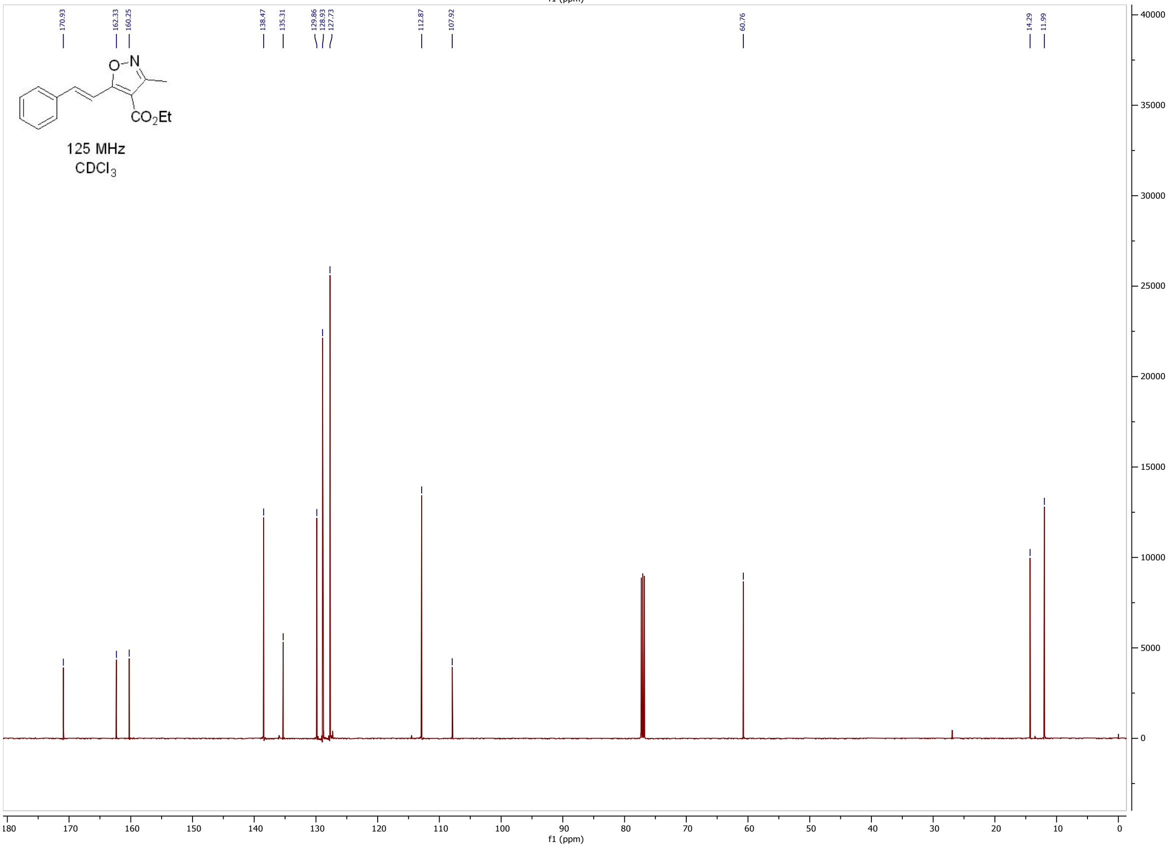

Ethyl 3-methyl-5-phenethylisoxazole-4-carboxylate (1m)

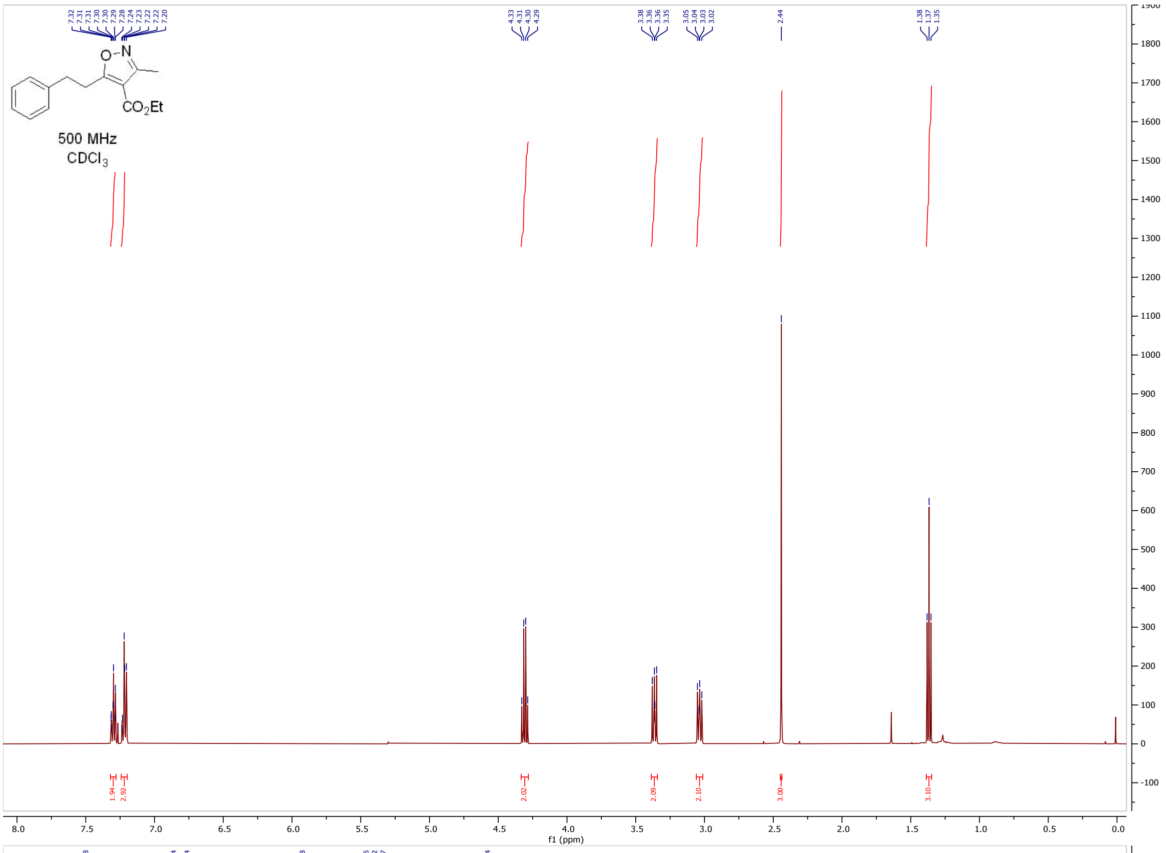

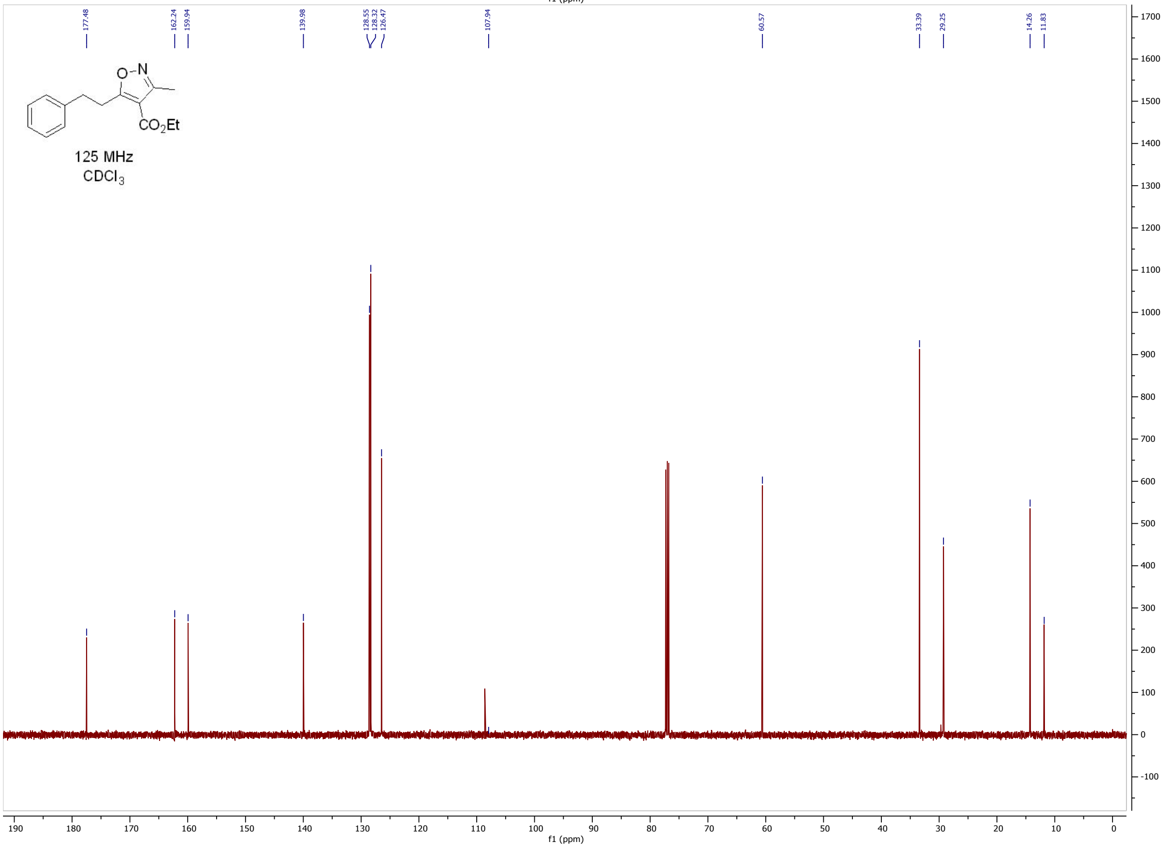

6.b Copies of NMR Spectra of Ketenimines 2a-i {Crude Products}

Ethyl 2-benzoyl-3-(methylimino)acrylate (2a)

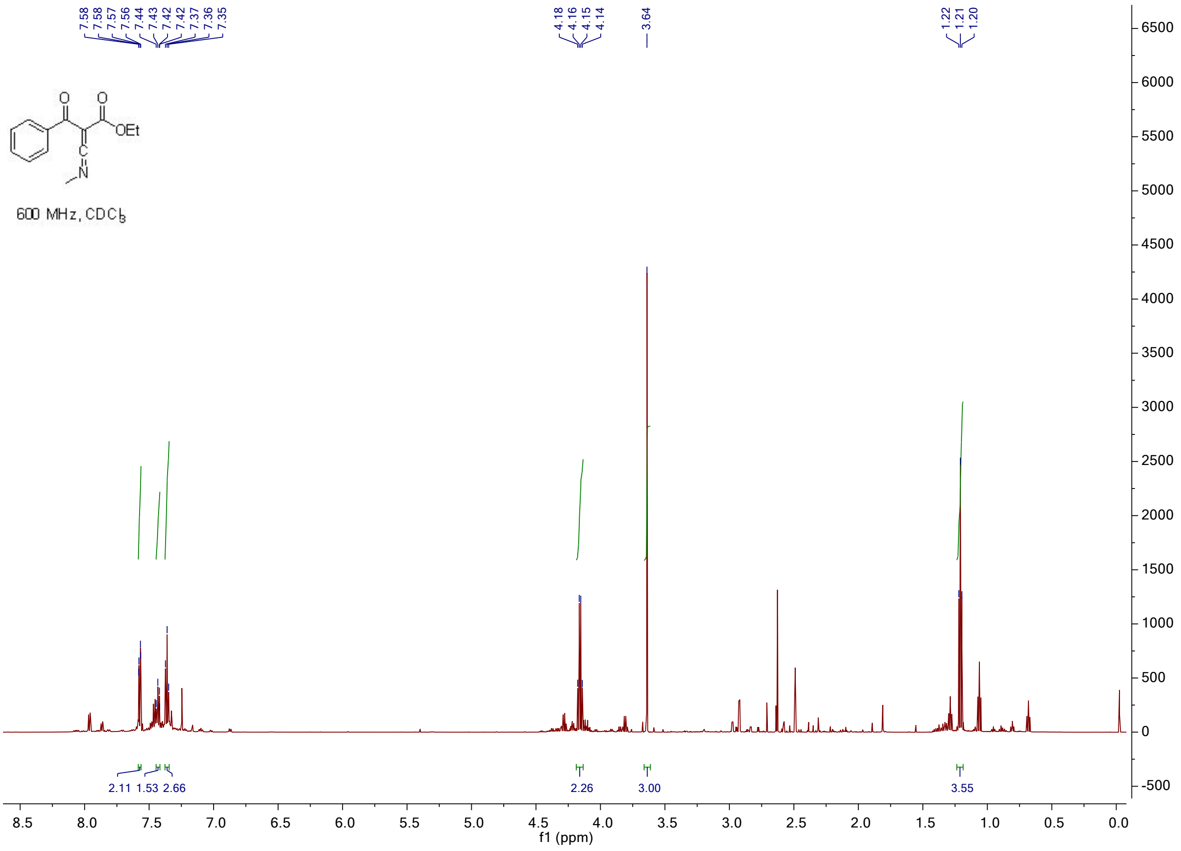

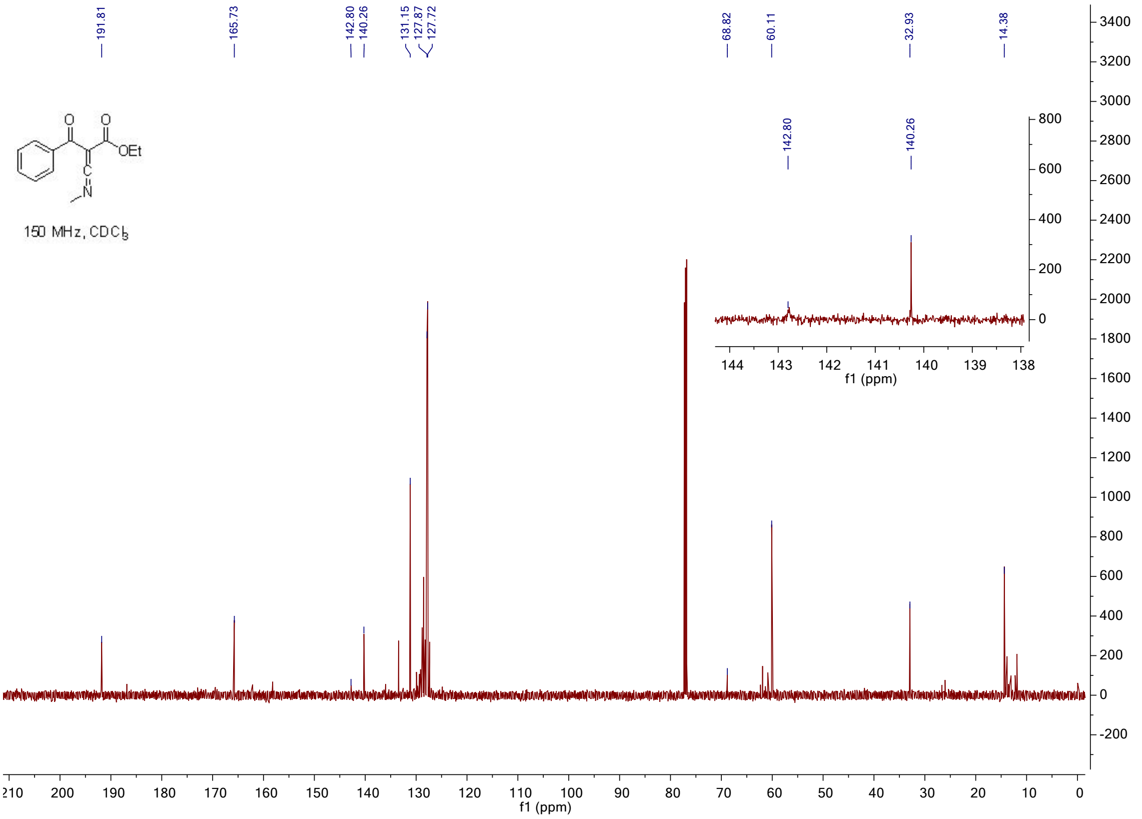

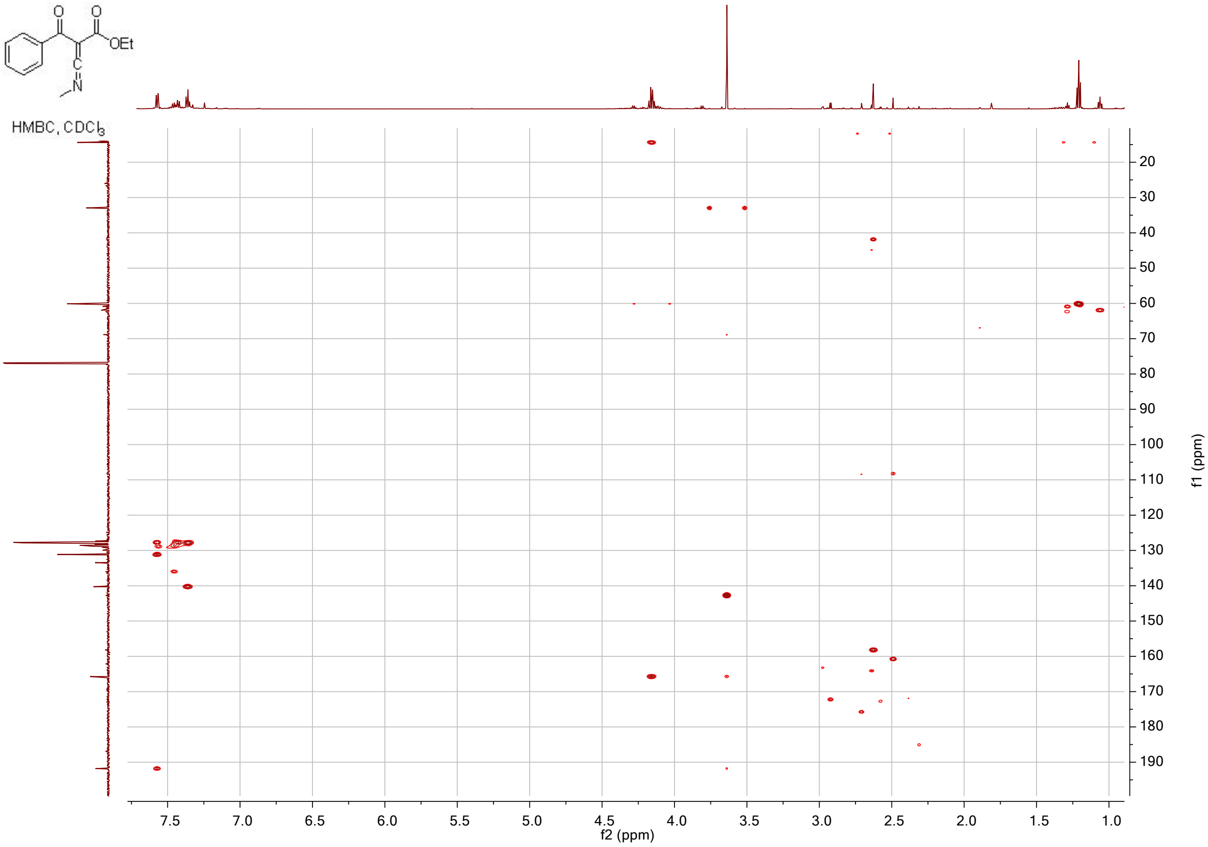

Ethyl 2-(benzo[*d*][1,3]dioxole-5-carbonyl)-3-(methylimino)acrylate (2b)

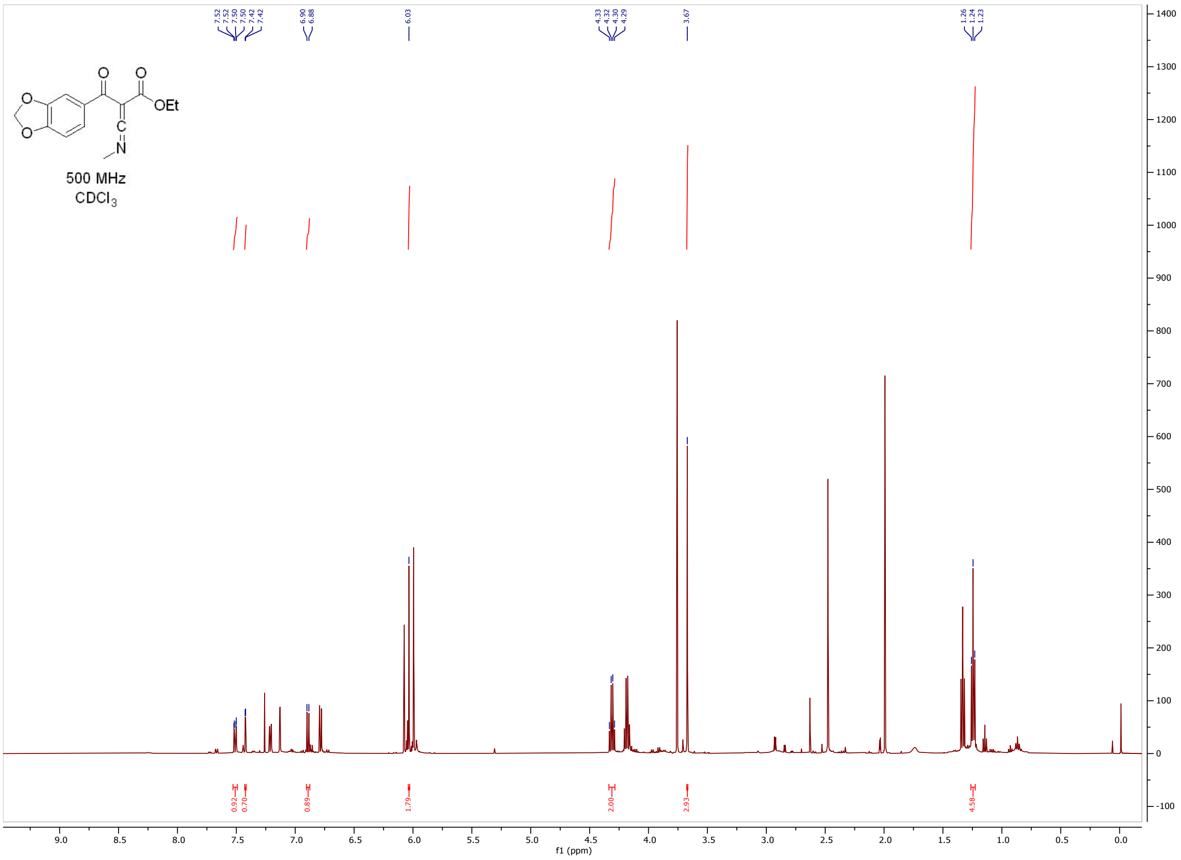

Ethyl 2-(4-methylbenzoyl)-3-(methylimino)acrylate (2c)

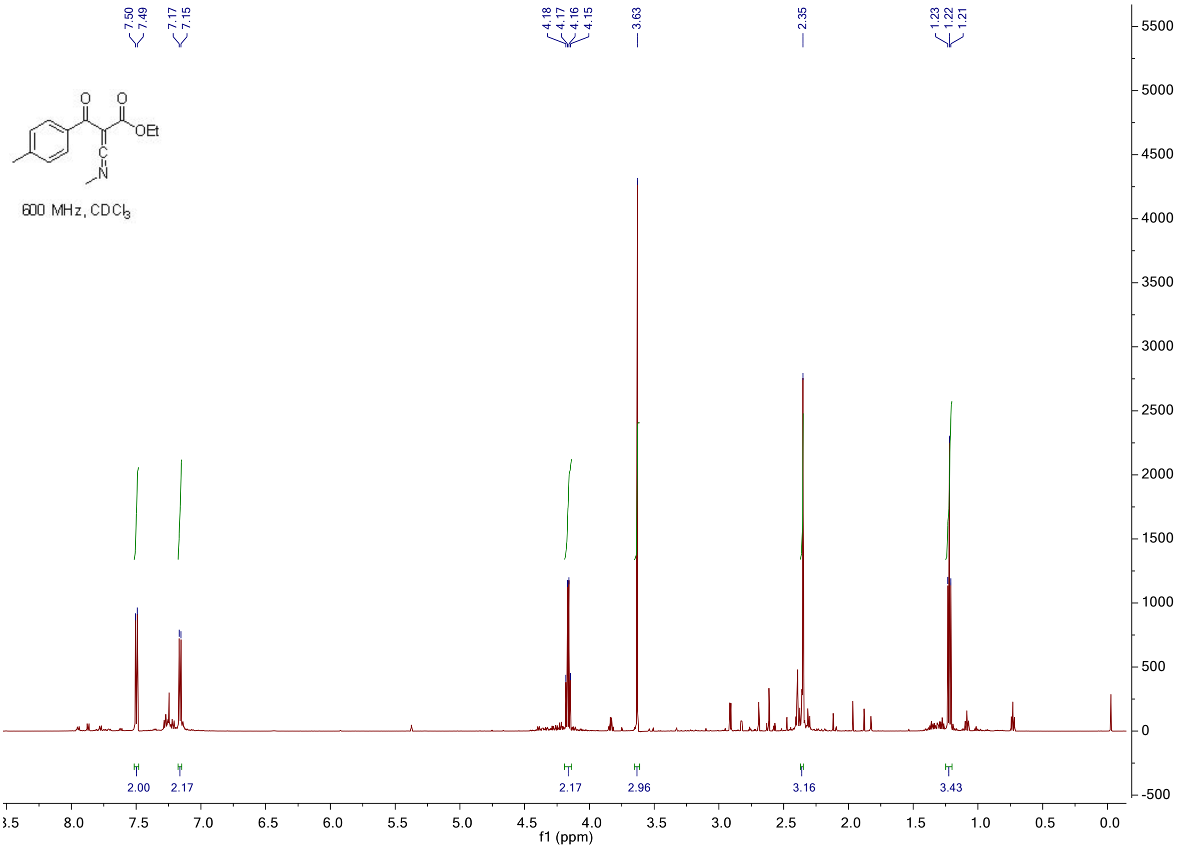

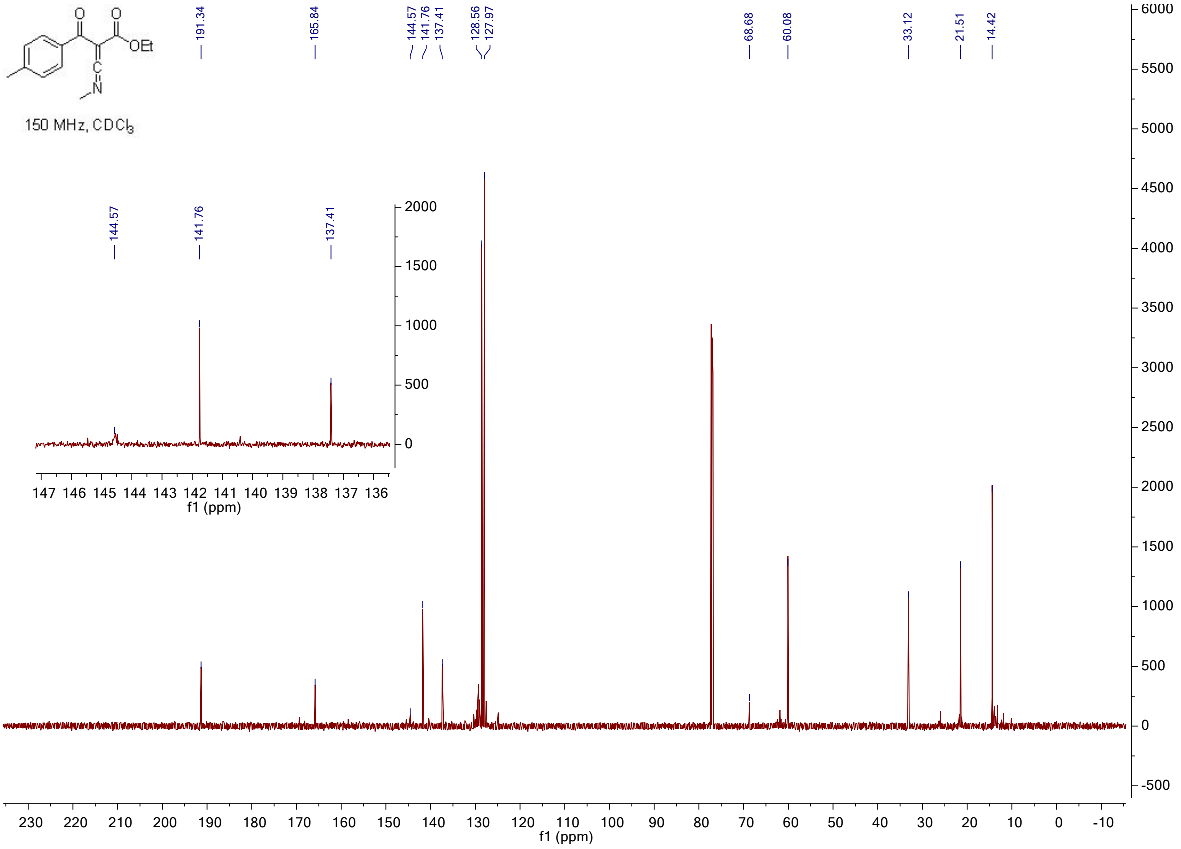

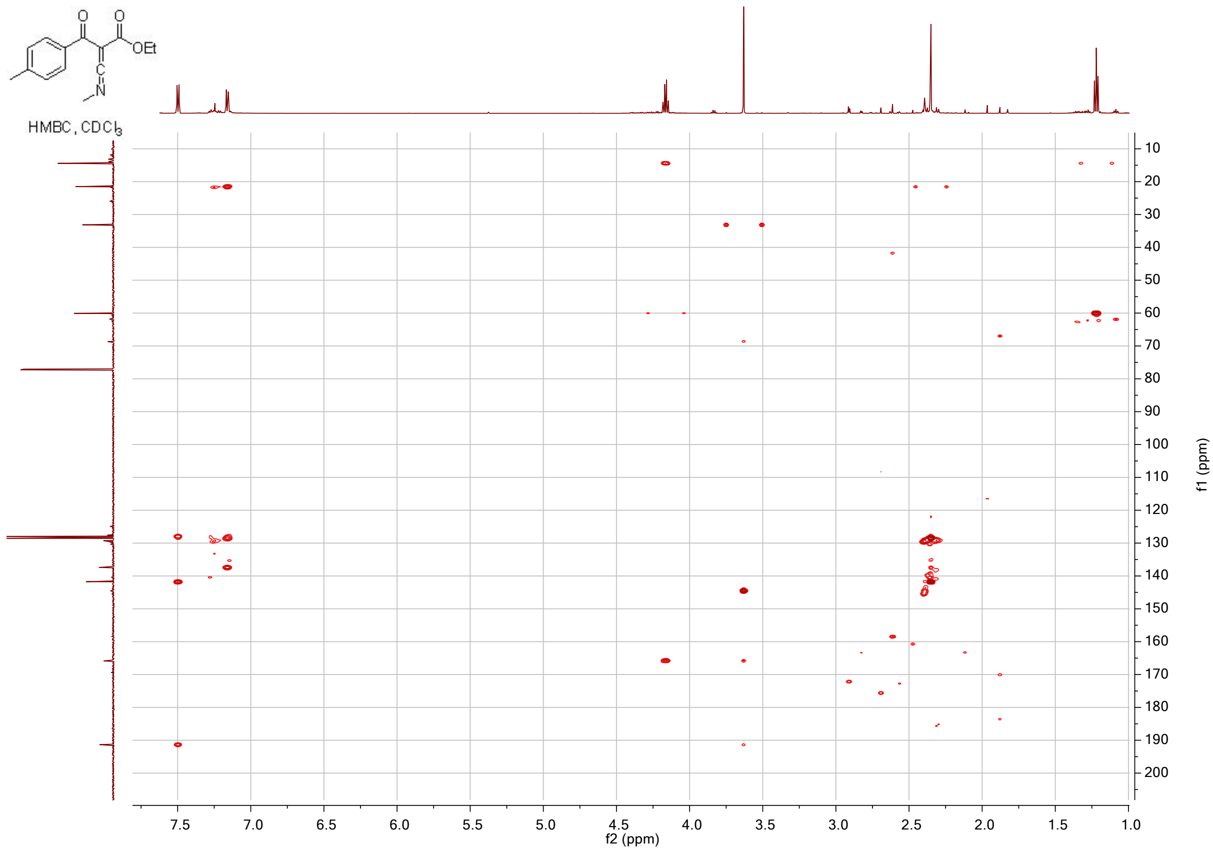

Ethyl 2-(4-bromobenzoyl)-3-(methylimino)acrylate (2d)

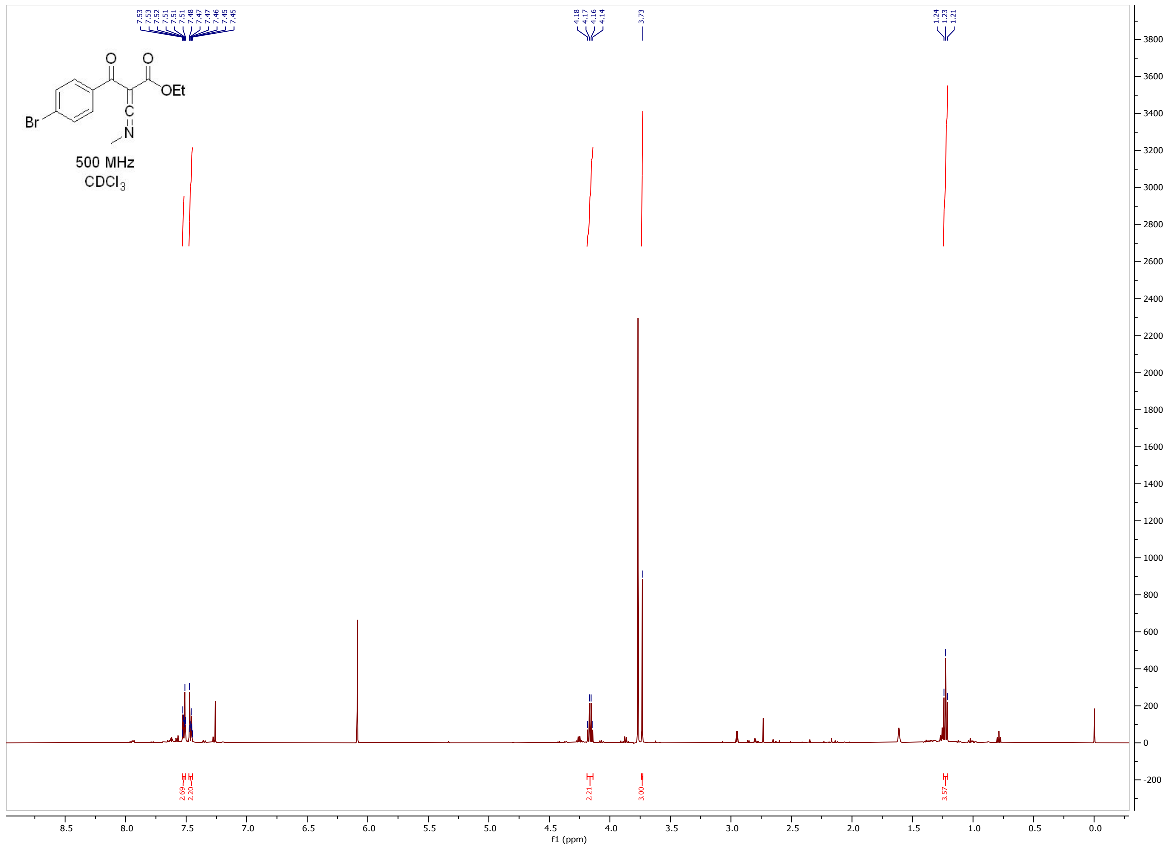

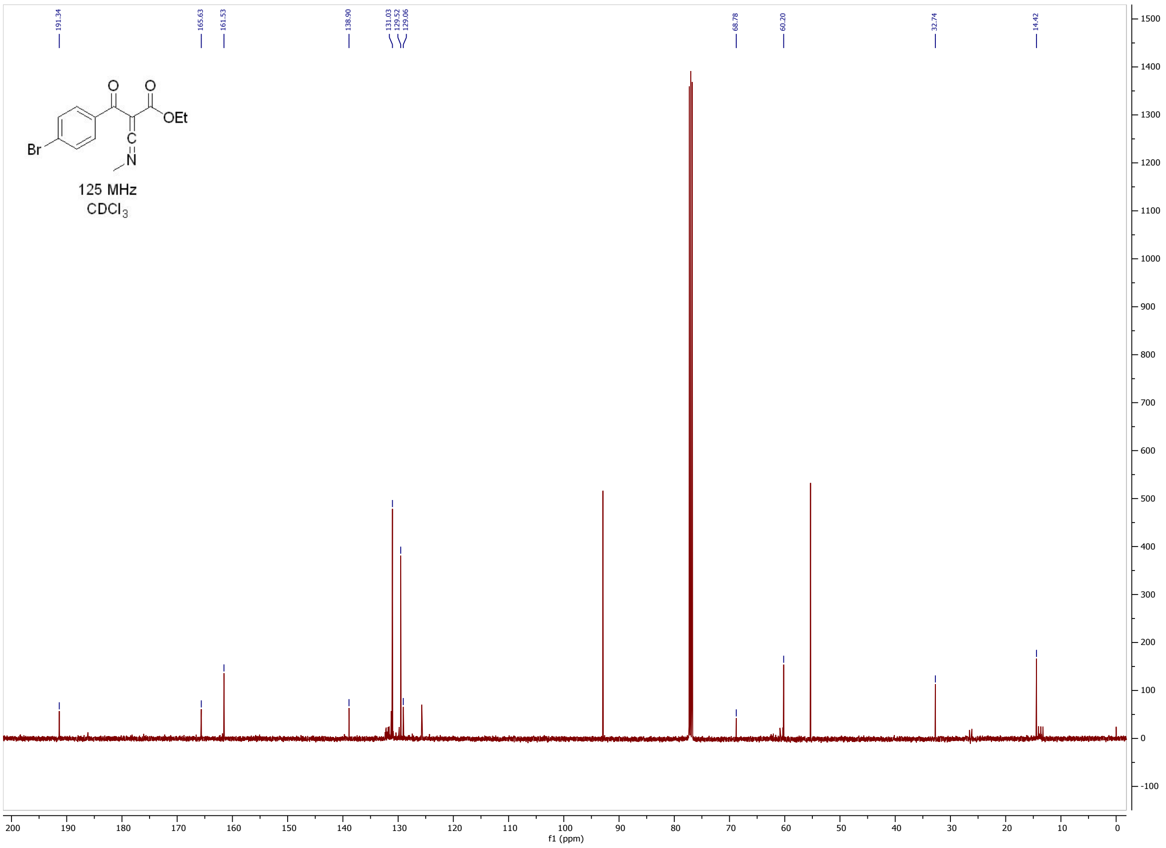

Ethyl 3-(methylimino)-2-(4-(trifluoromethyl)benzoyl)acrylate (2e)

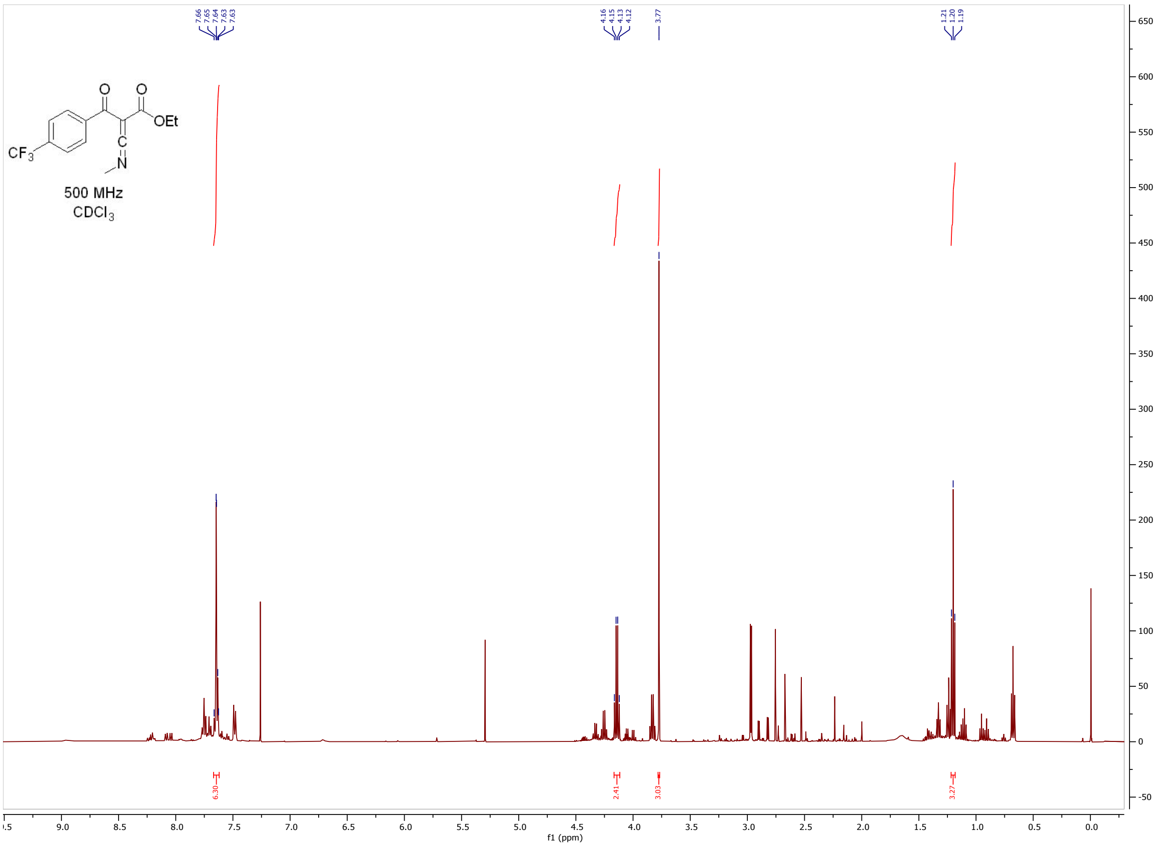

Ethyl 2-(4-chloro-3-methylbenzoyl)-3-(methylimino)acrylate (2f)

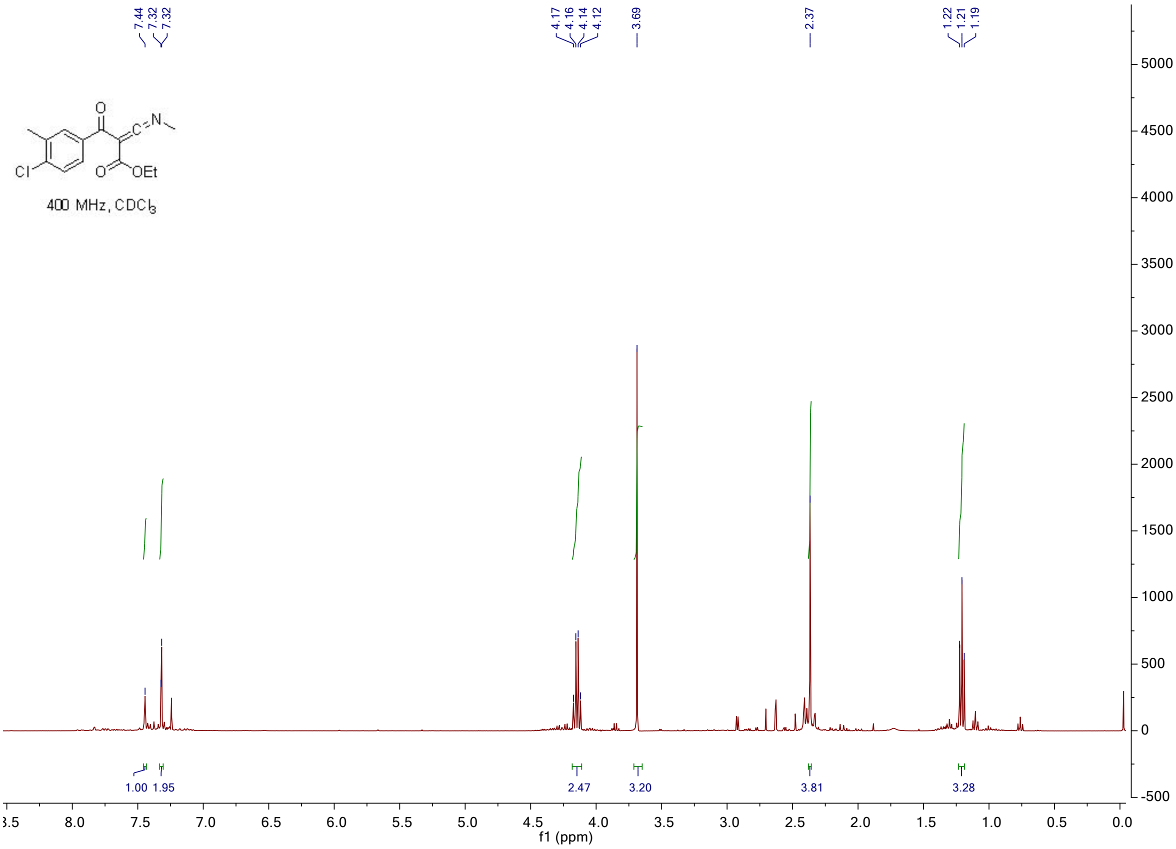

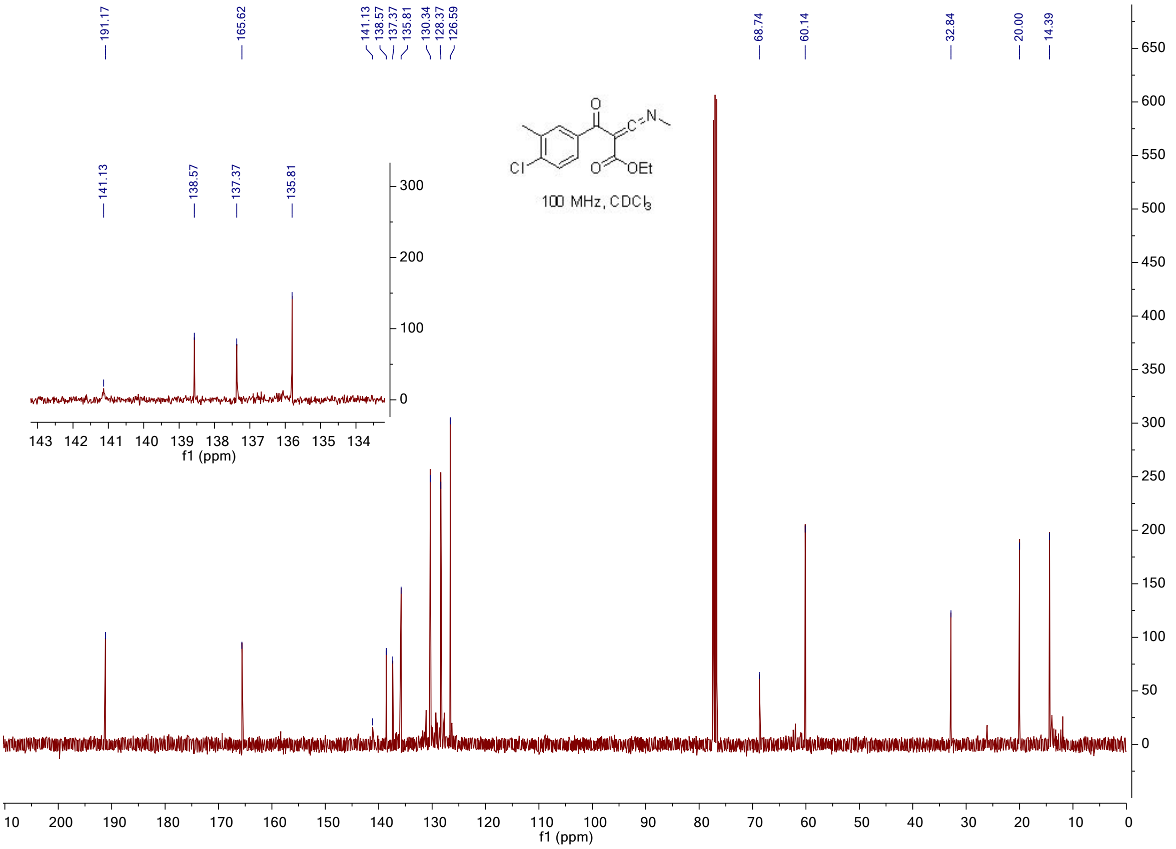

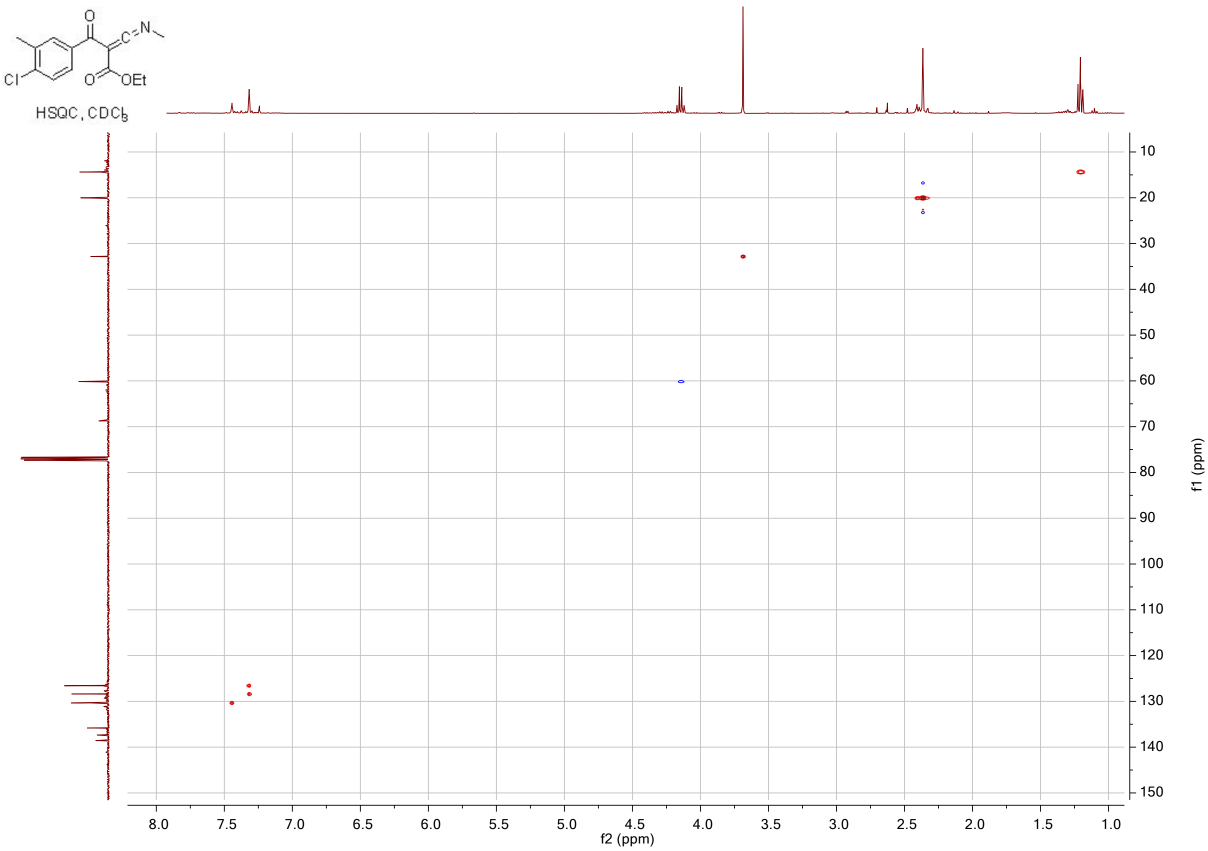
O
N
C
Cl
O
OEt
HSQC, CDCl3
f2 (ppm)
f1 (ppm)

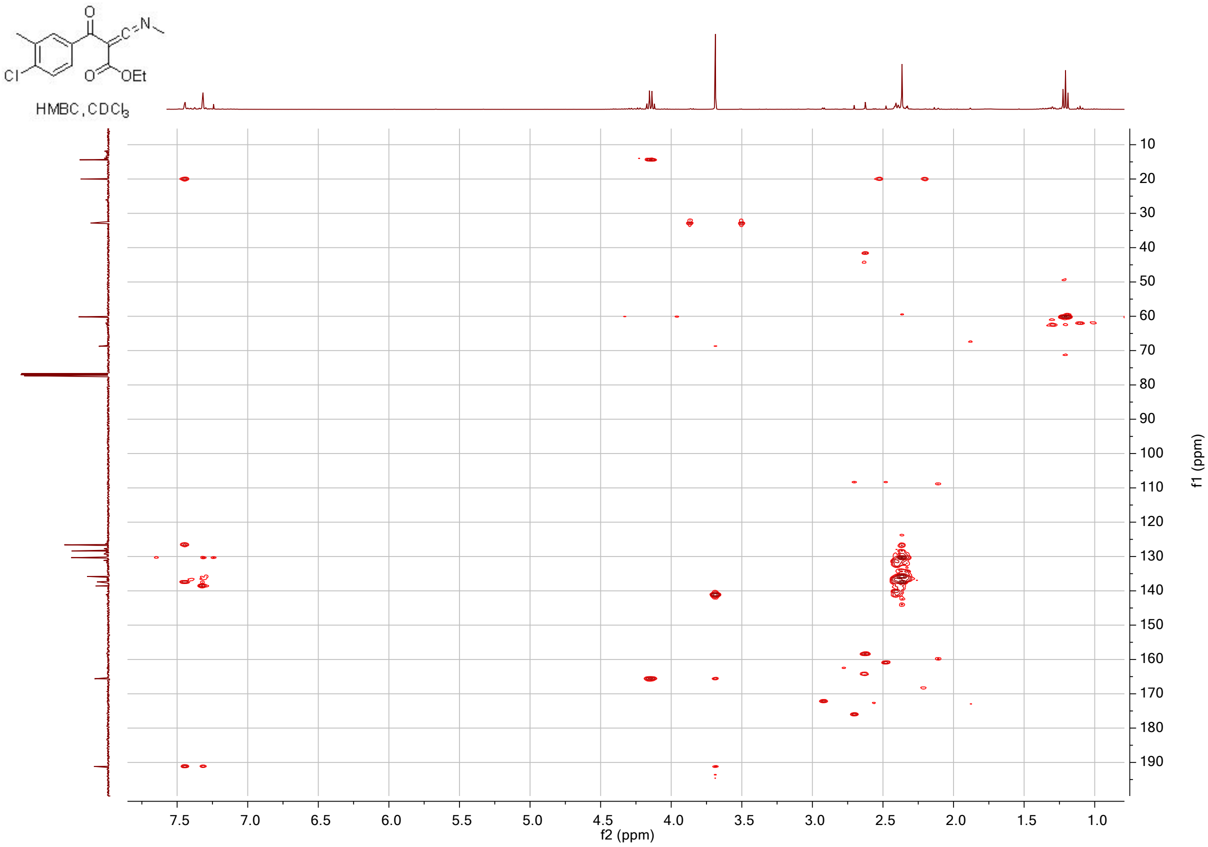
O
N
C
Cl
O
OEt
HMBC, CDCl3
f2 (ppm)
f1 (ppm)

Ethyl 3-(methylimino)-2-(4-(trifluoromethoxy)benzoyl)acrylate (2g)

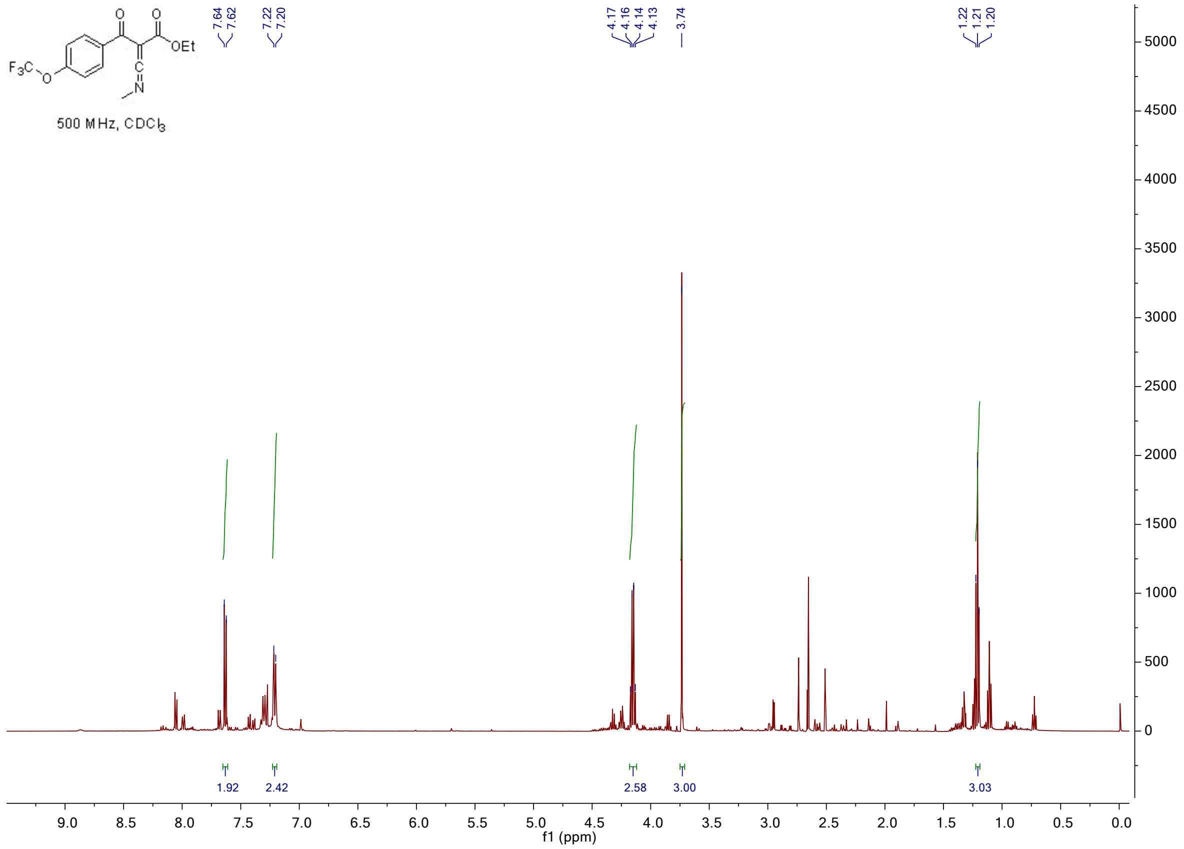

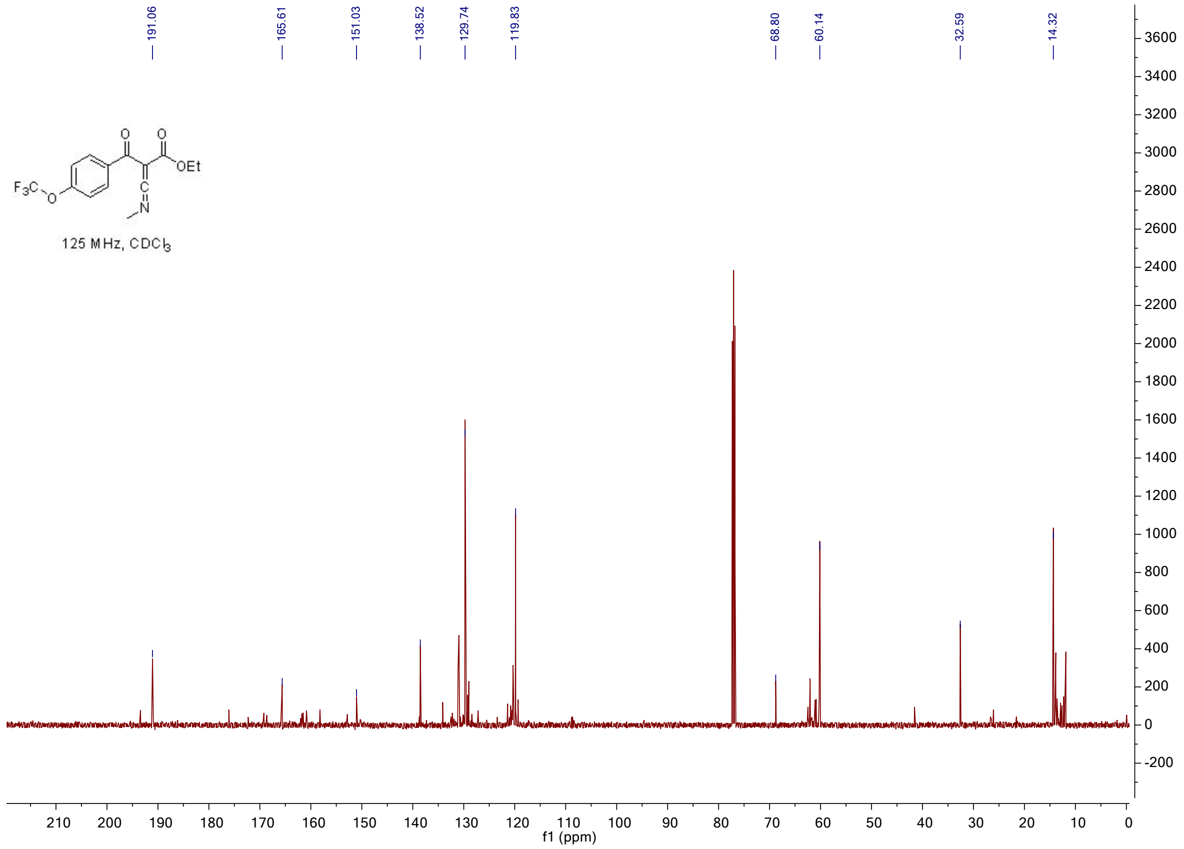

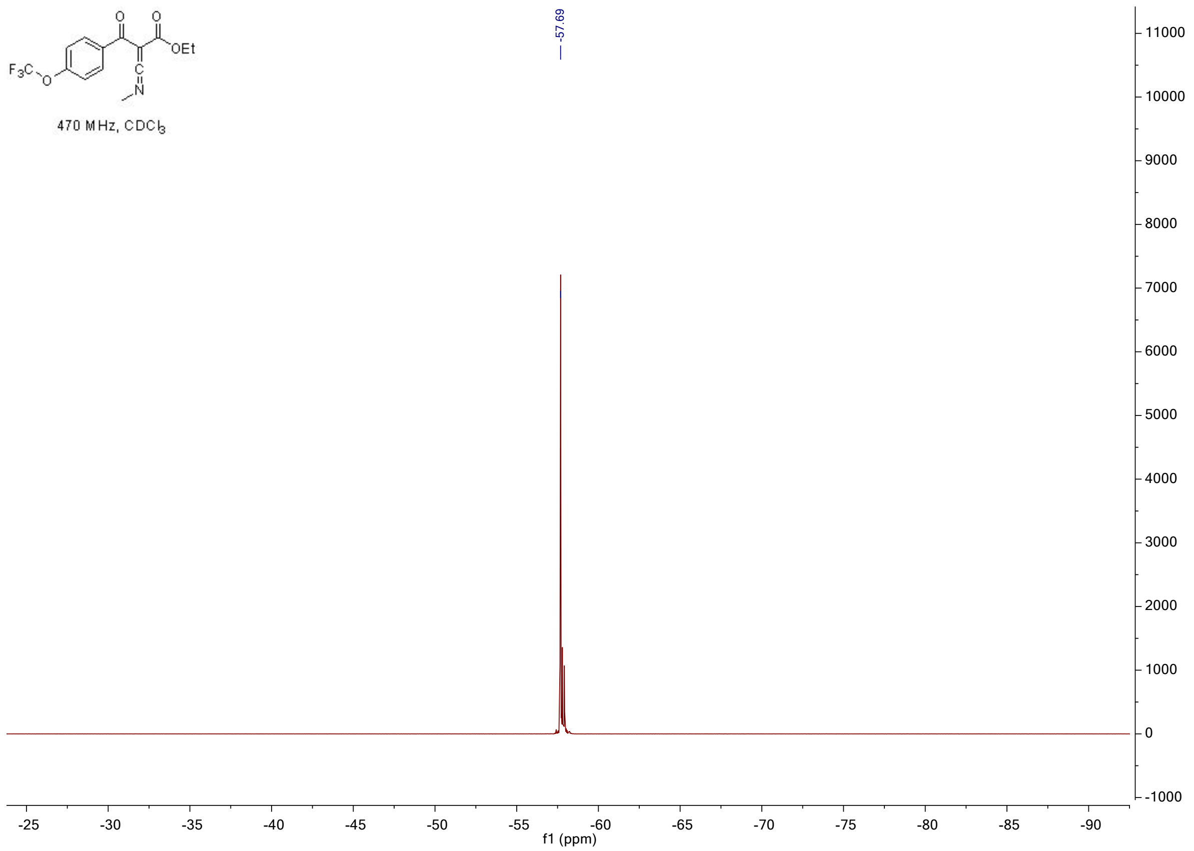

OEt
F3C
O
C
N
470 MHz, CDCl3
-57.69
f1 (ppm)

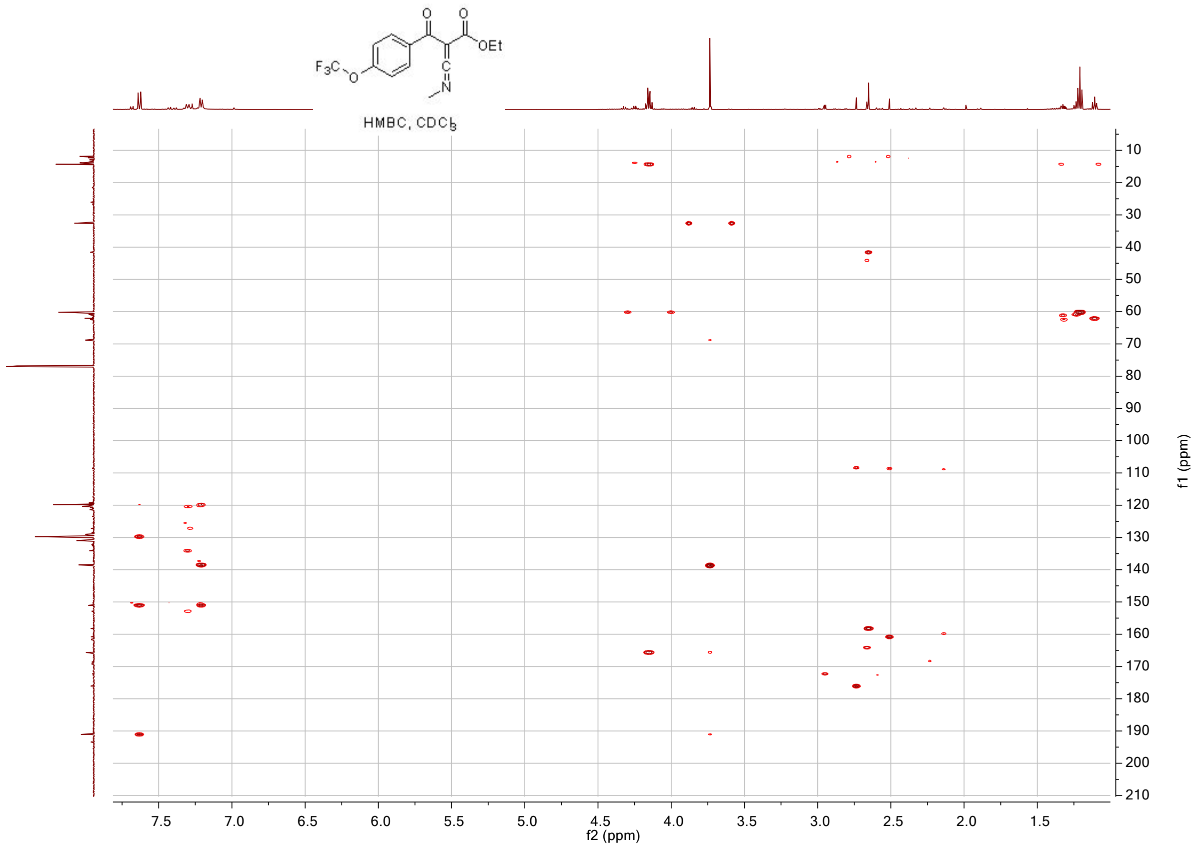

OEt
F3C
O
C
N
HMBC, CDCl3
f2 (ppm)
f1 (ppm)

Ethyl 2-(3,5-dichlorobenzoyl)-3-(methylimino)acrylate (2h)

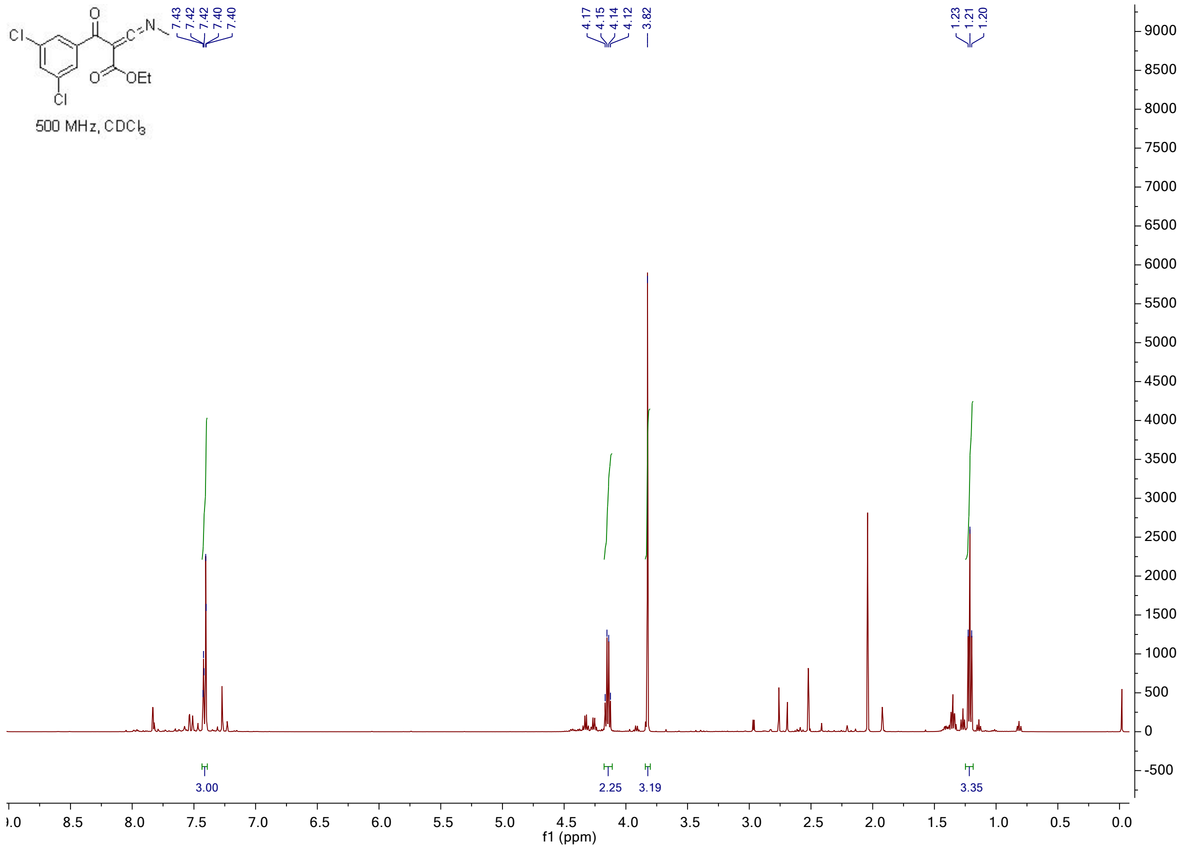

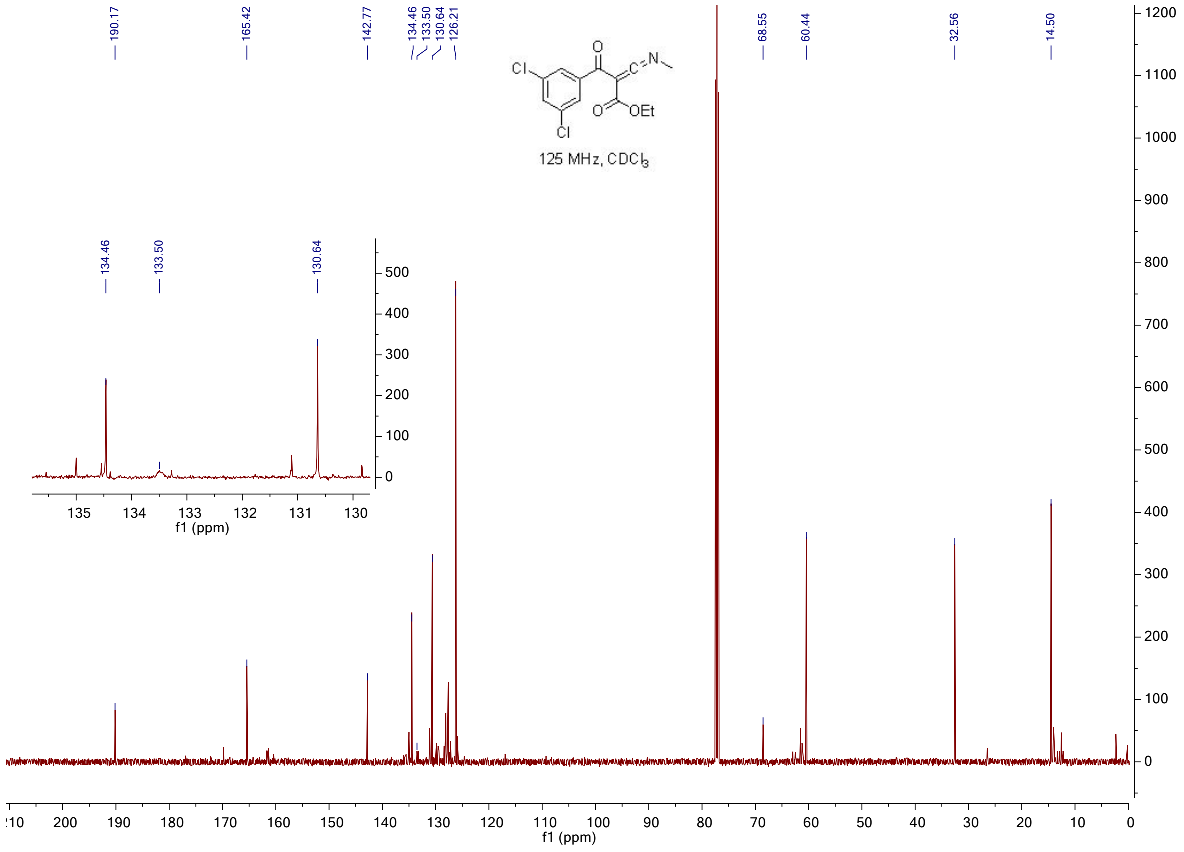

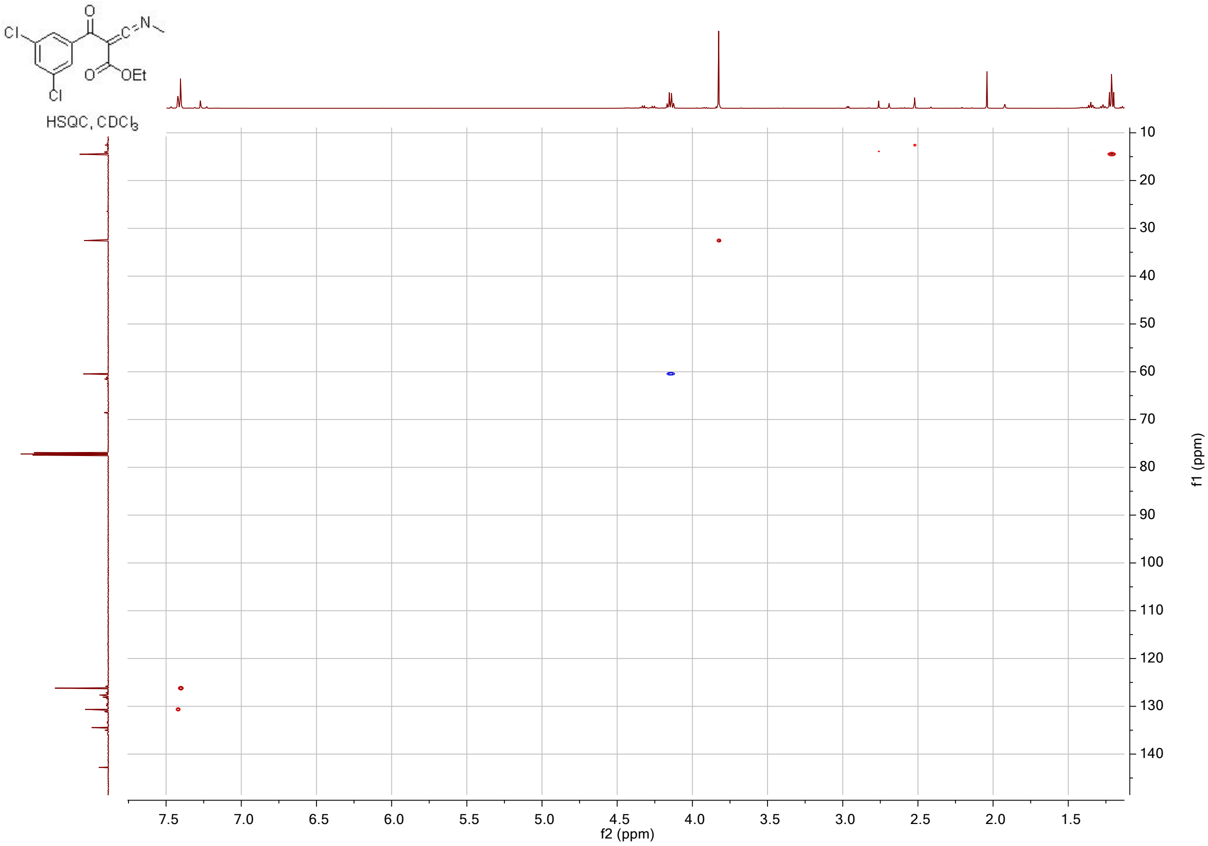
HSQC, CDCl3
f2 (ppm)
f1 (ppm)

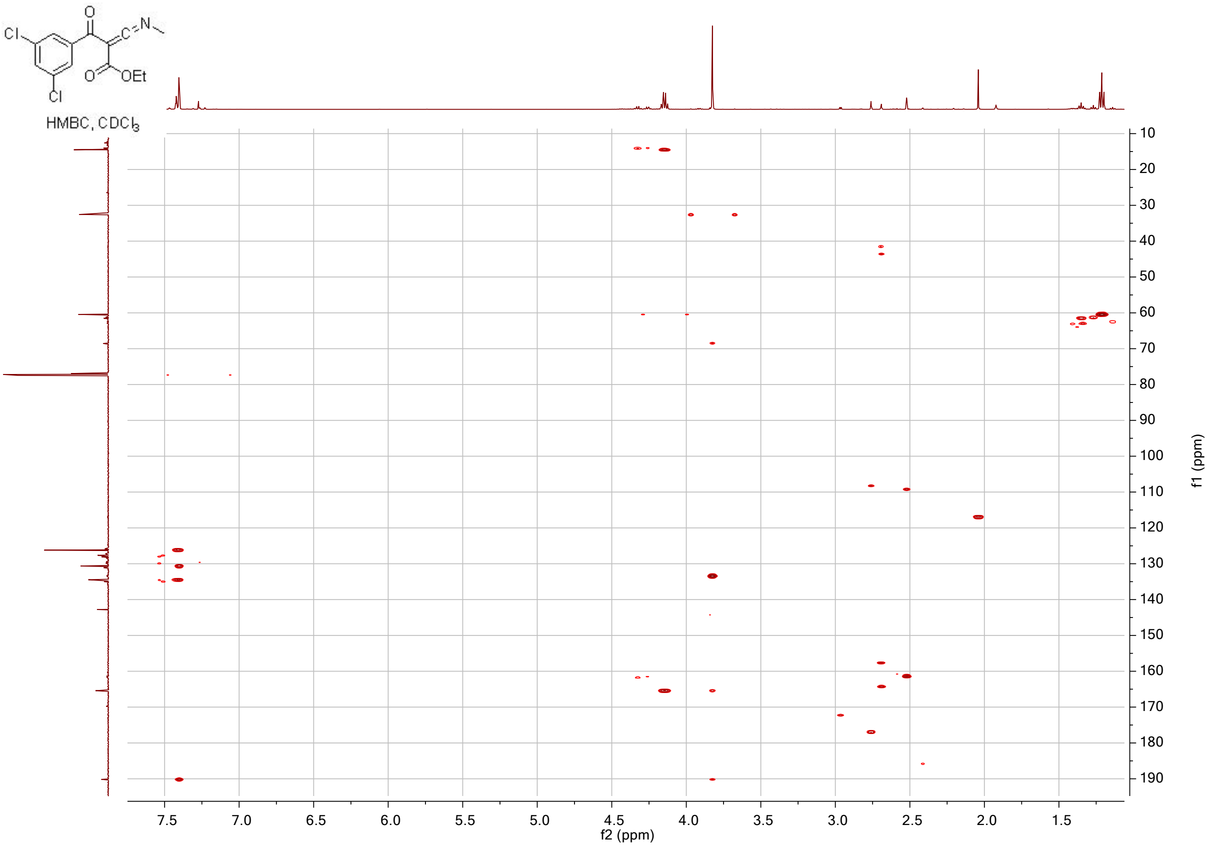
HMBC, CDCl3
f2 (ppm)
f1 (ppm)

Ethyl 2-(2-fluorobenzoyl)-3-(methylimino)acrylate (2i)

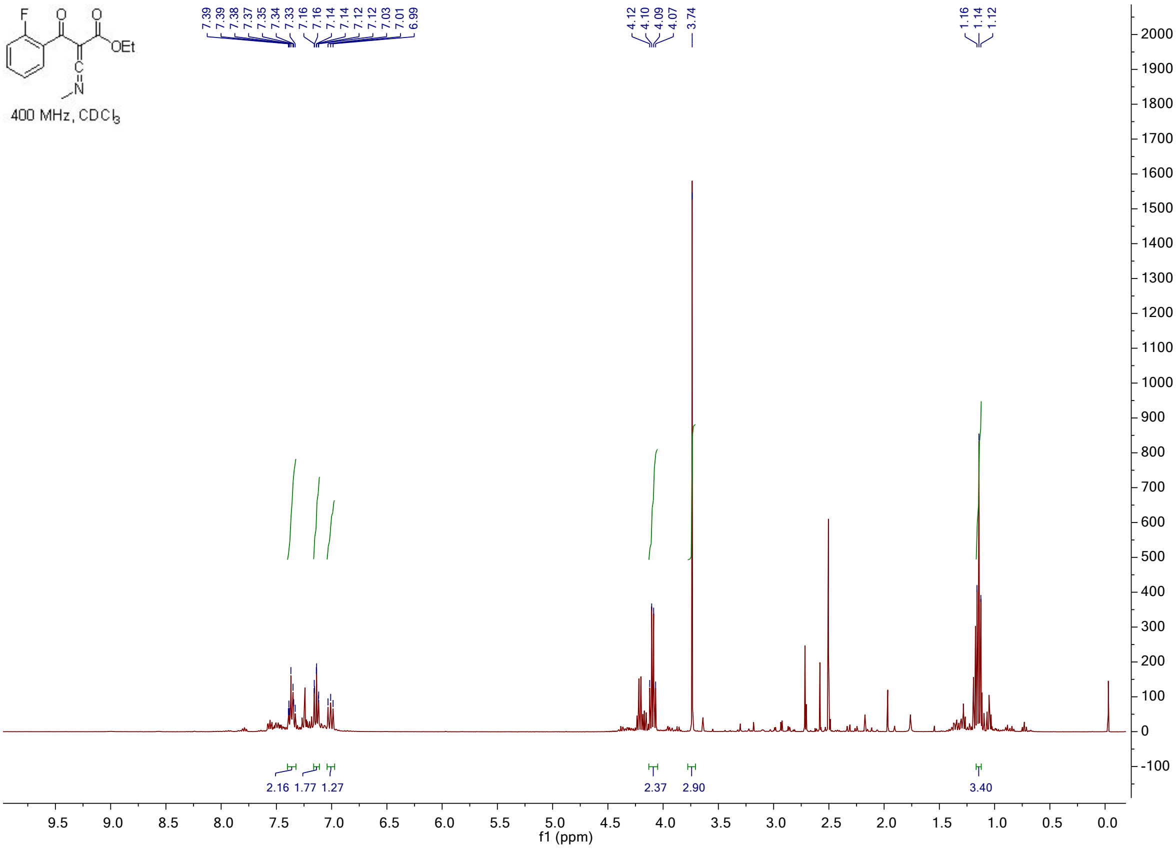

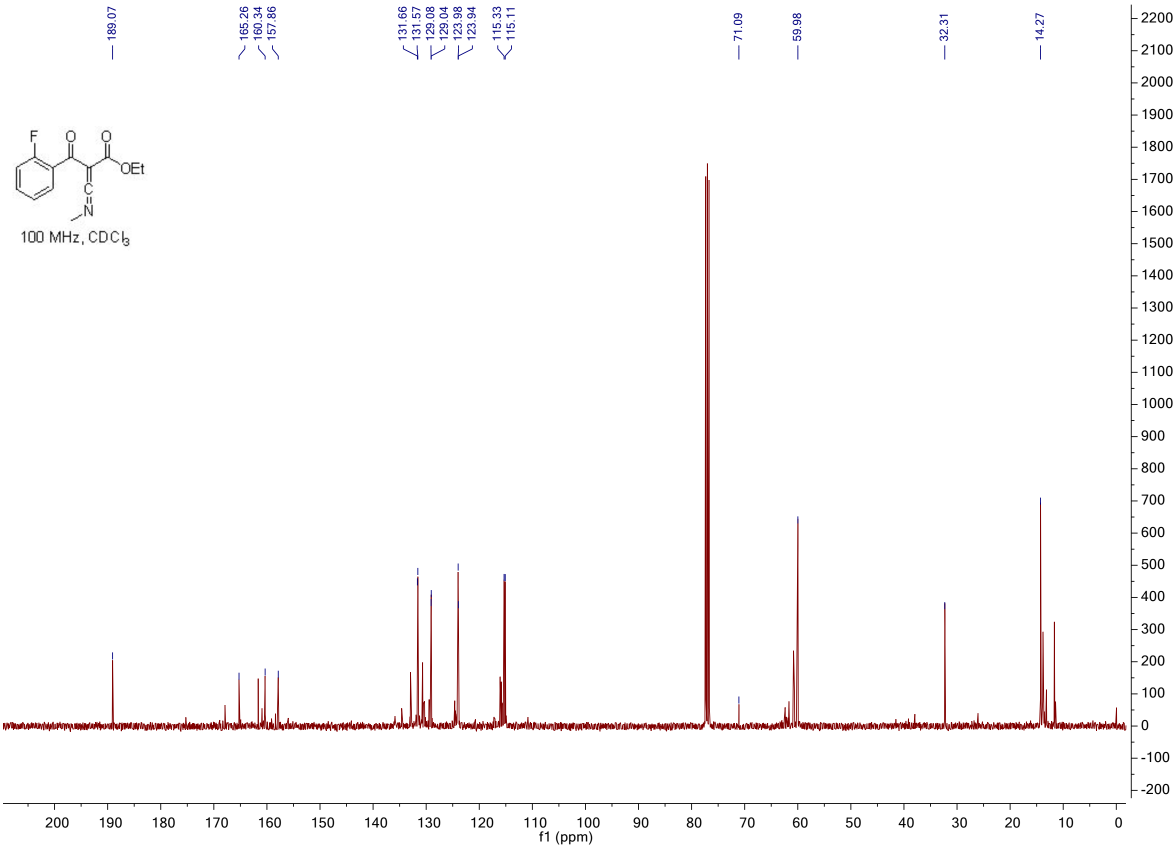

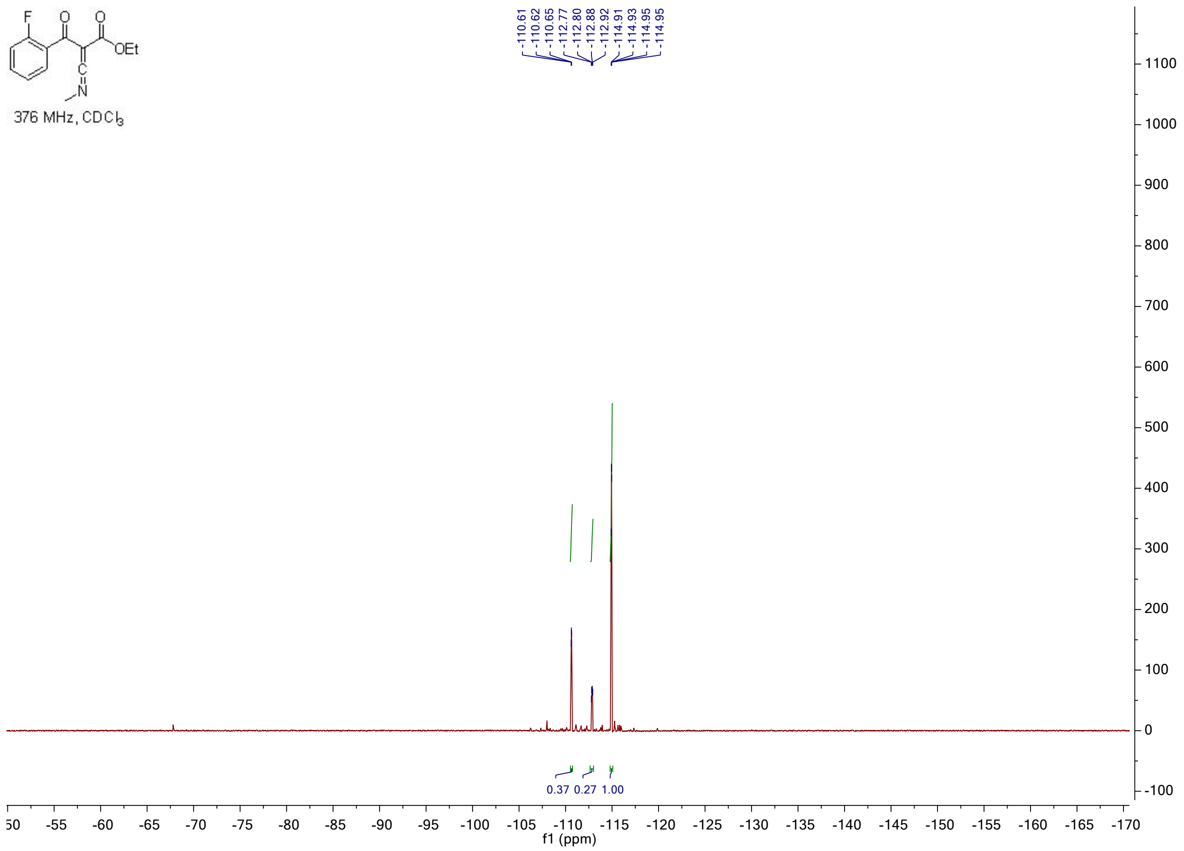
376 MHz, CDCl3
-110.61
-110.62
-110.65
-112.77
-112.80
-112.88
-112.92
-114.91
-114.93
-114.95
-114.95
0.37 0.27 1.00
f1 (ppm)

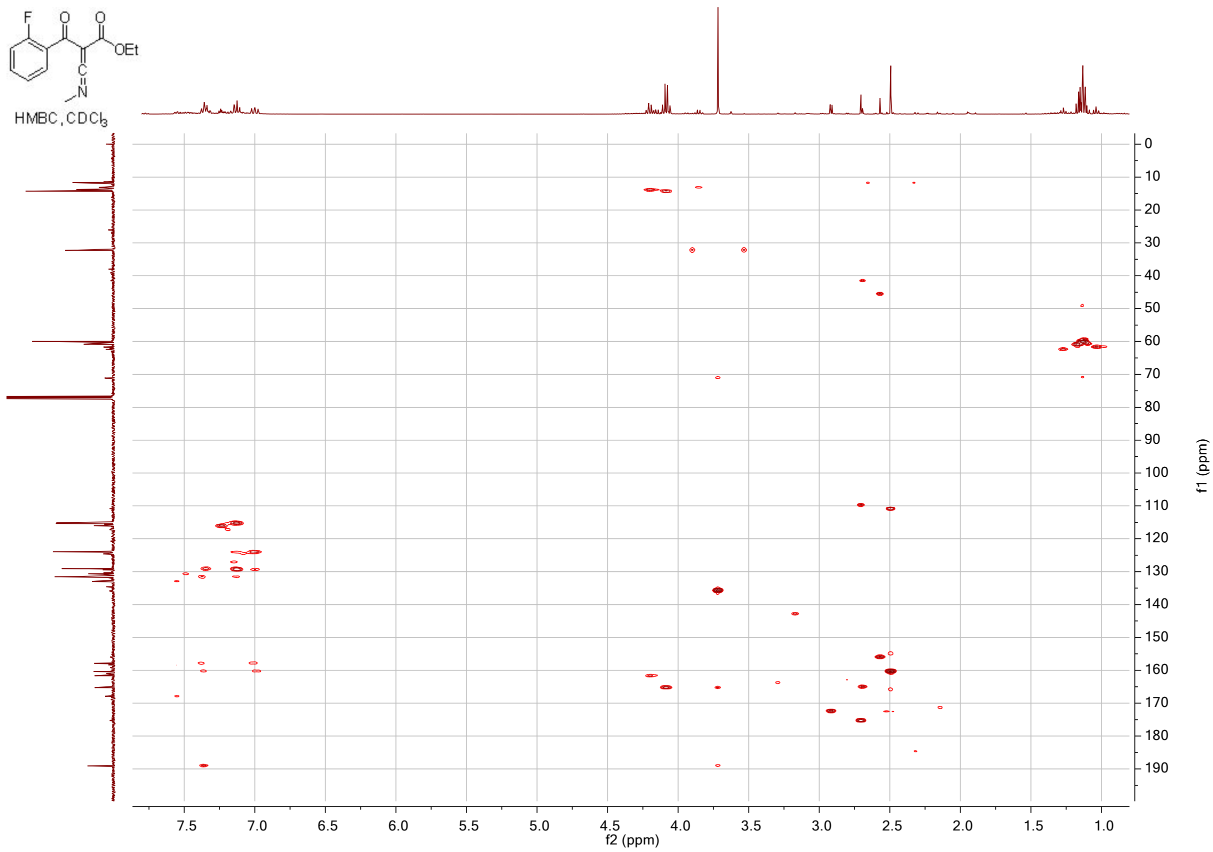
HMBC, CDCl3
f2 (ppm)
f1 (ppm)

Ethyl 2-(3-chlorobenzoyl)-3-(methylimino)acrylate (2j)

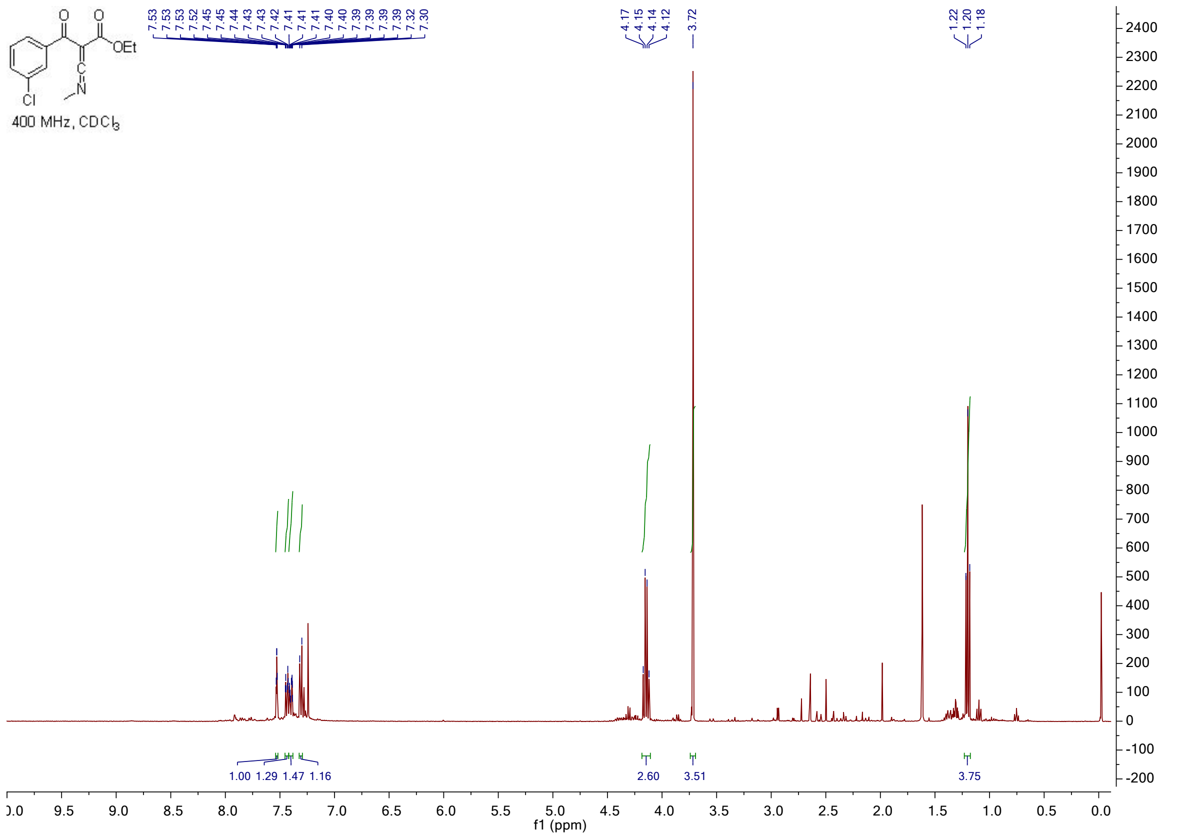

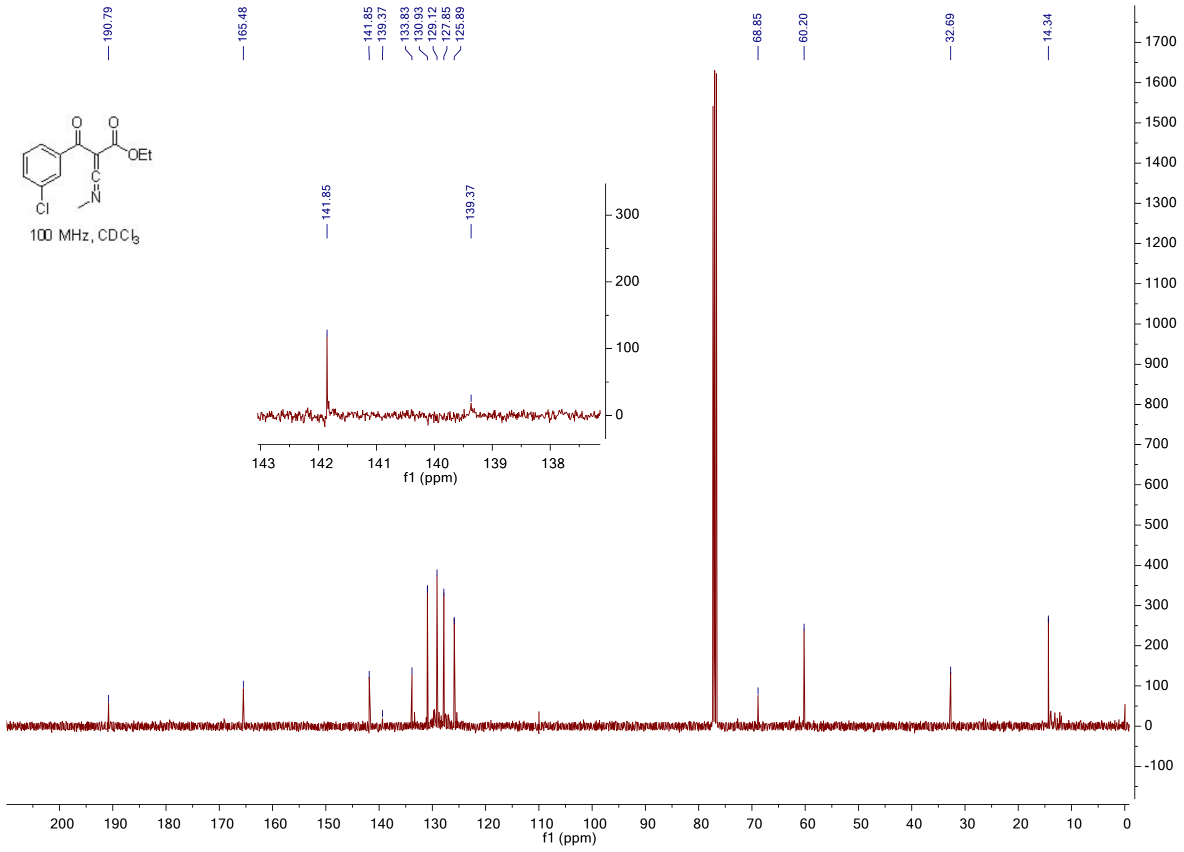

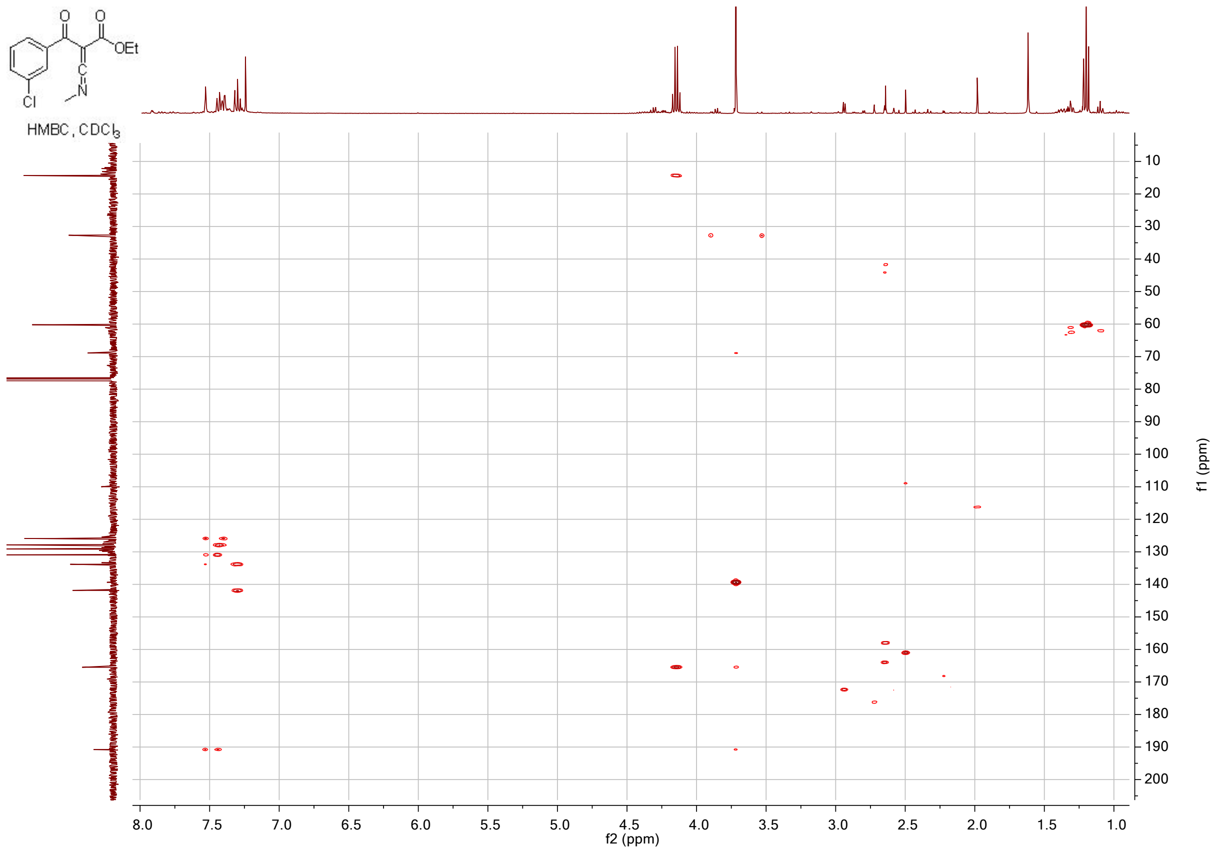

O
O
OEt
C
N
Cl
HMBC, CDCl3
f1 (ppm)
f2 (ppm)

6.c Copies of NMR Spectra of Pyrazoles 4a-f and 5a/a'-e/e'

Ethyl 3-(methylamino)-5-phenyl-1*H*-pyrazole-4-carboxylate (4a)

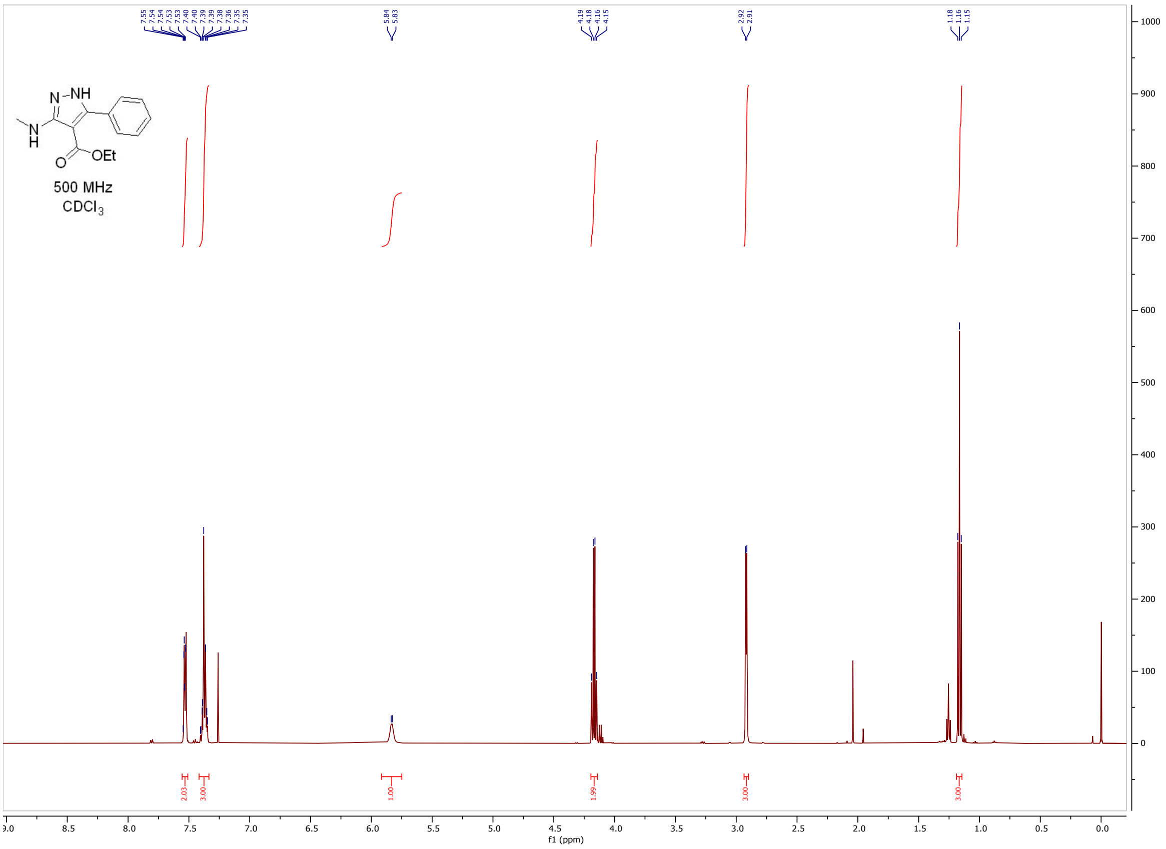

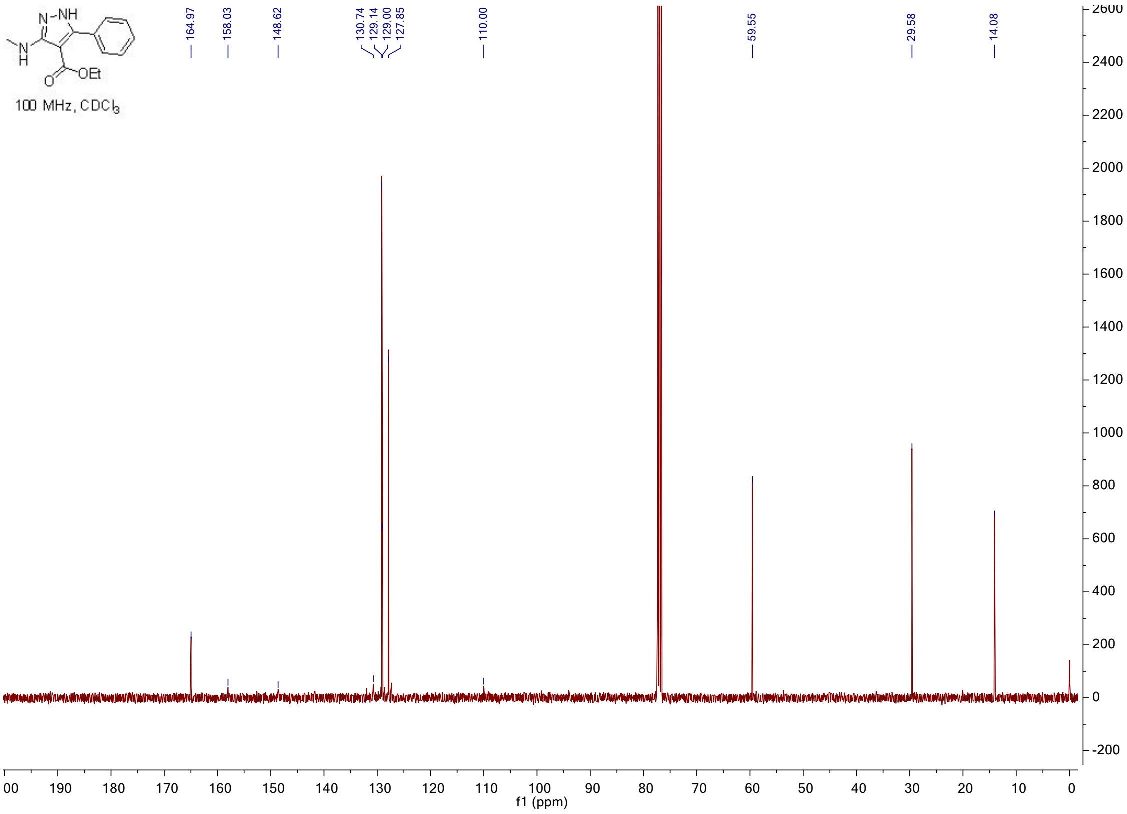

Ethyl 5-(benzo[*d*][1,3]dioxol-5-yl)-3-(methylamino)-1*H*-pyrazole-4-carboxylate (4b)

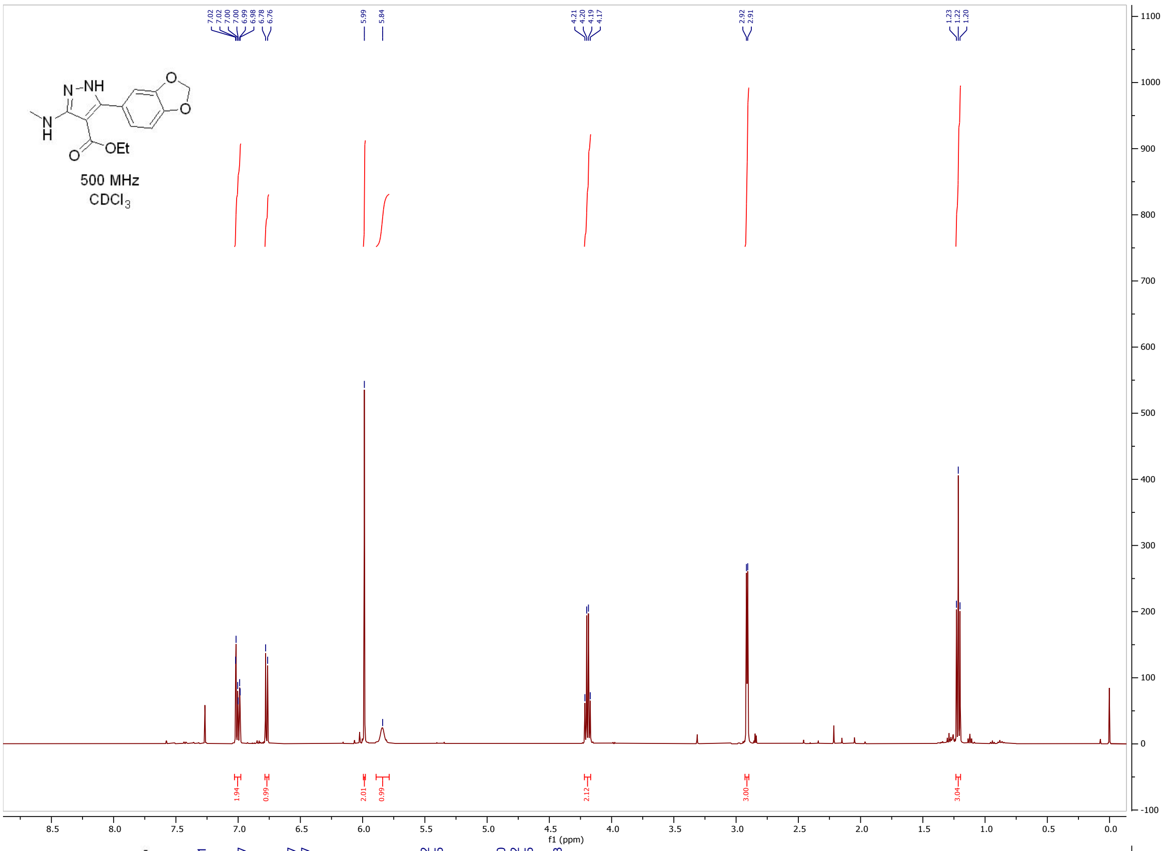

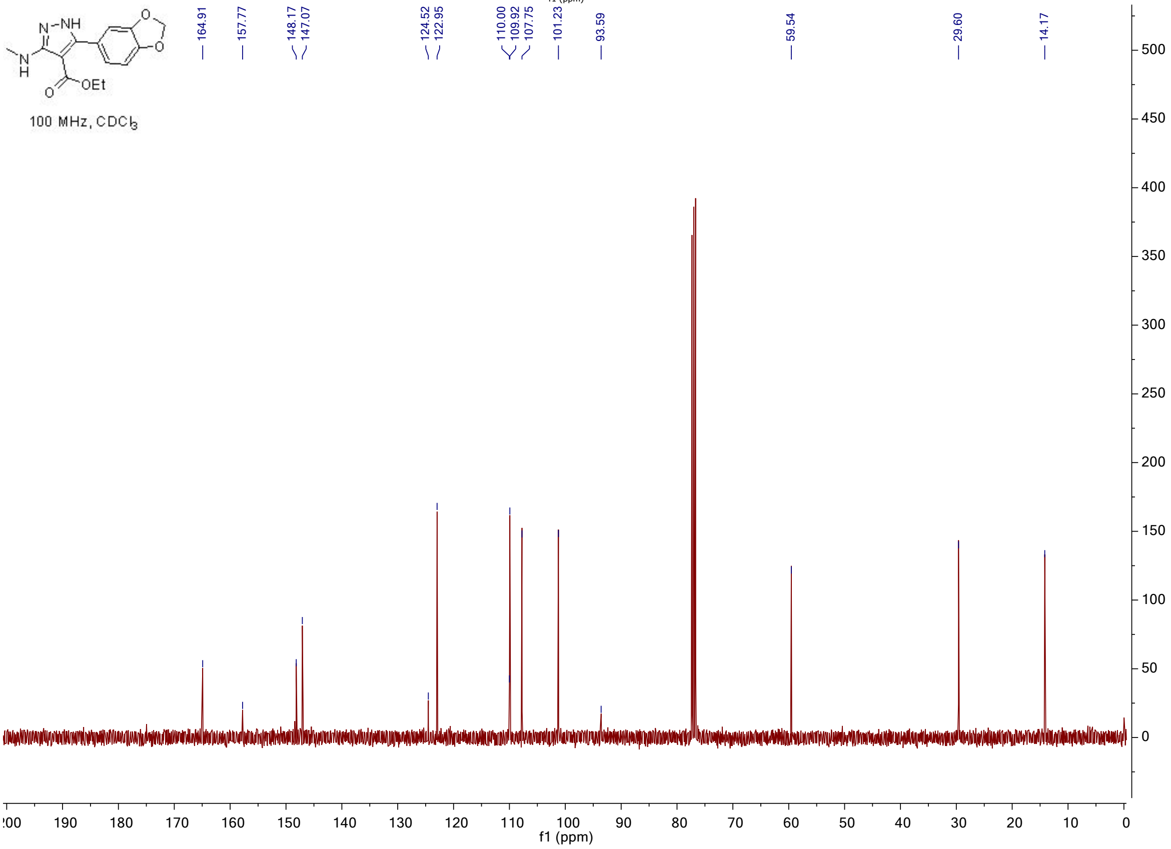

Ethyl 3-(methylamino)-5-(*p*-tolyl)-1*H*-pyrazole-4-carboxylate (4c)

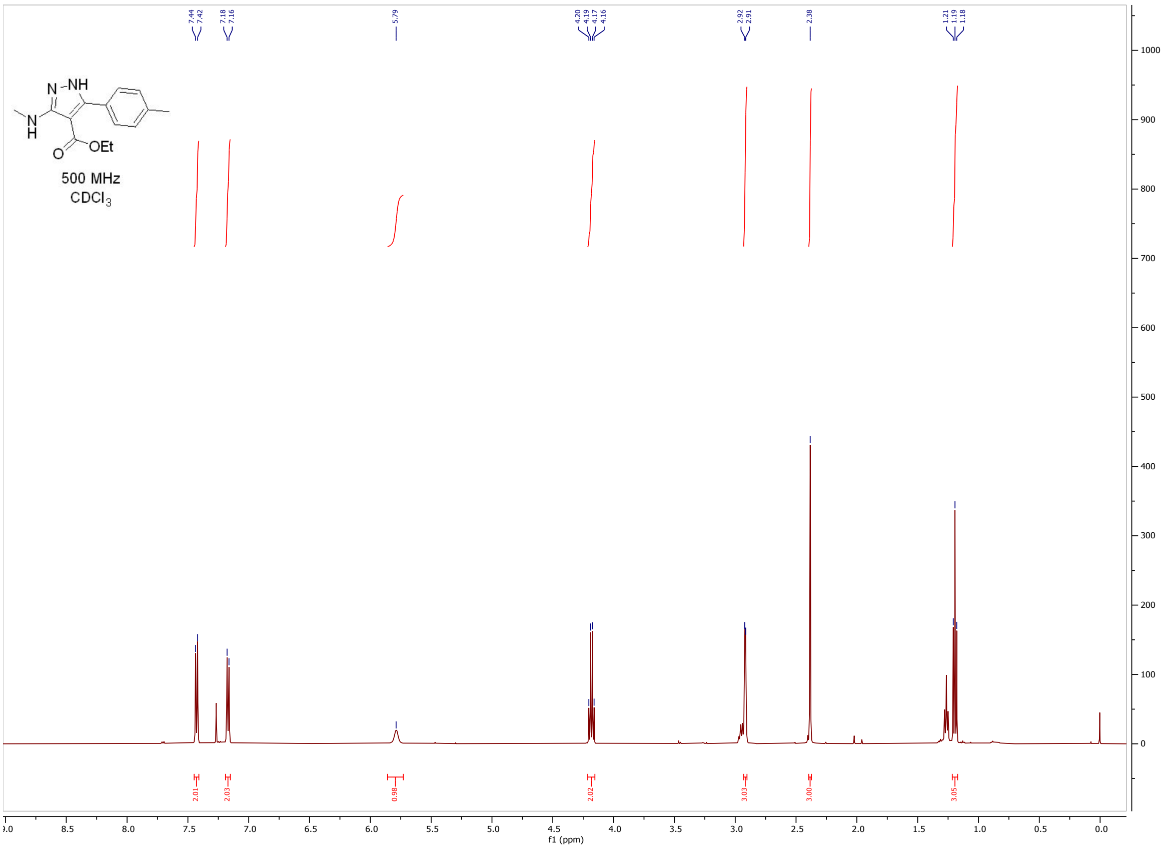

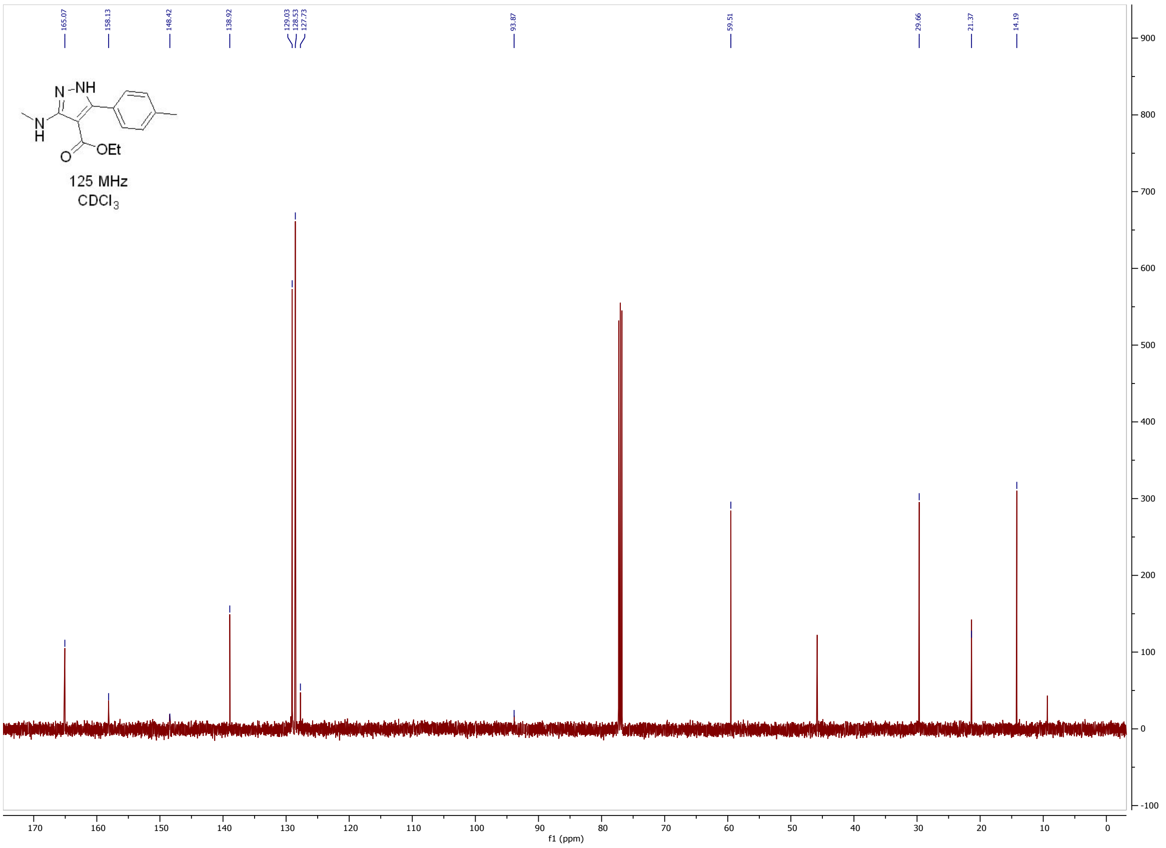

Ethyl 5-(4-bromophenyl)-3-(methylamino)-1*H*-pyrazole-4-carboxylate (4d)

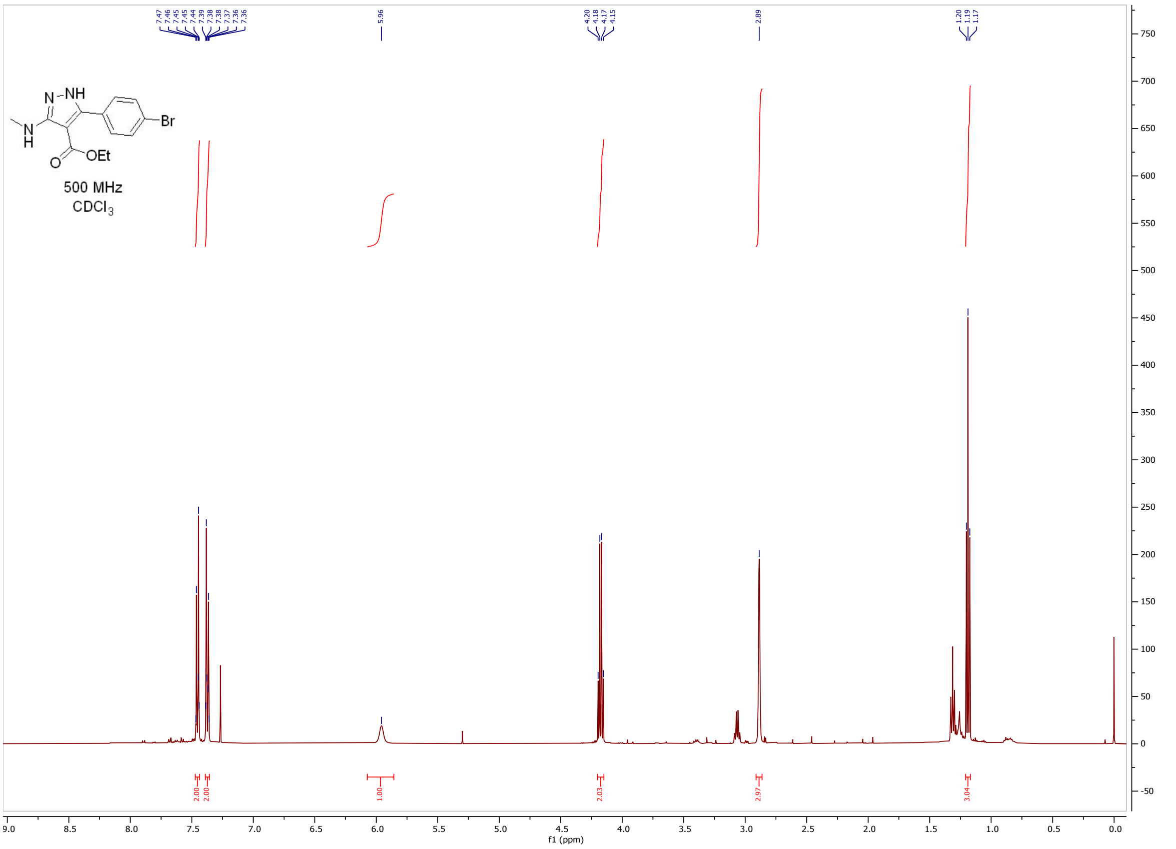

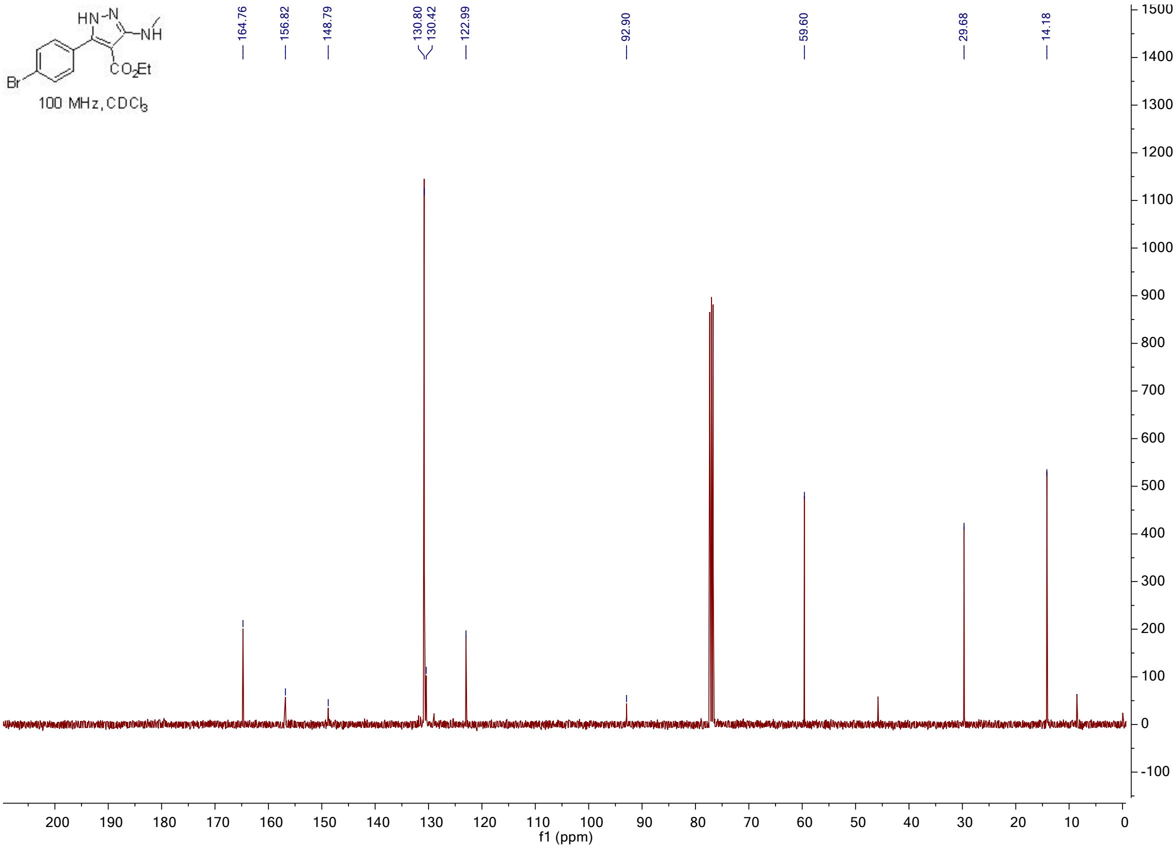

Ethyl 3-(methylamino)-5-(4-(trifluoromethoxy)phenyl)-1*H*-pyrazole-4-carboxylate (4e)

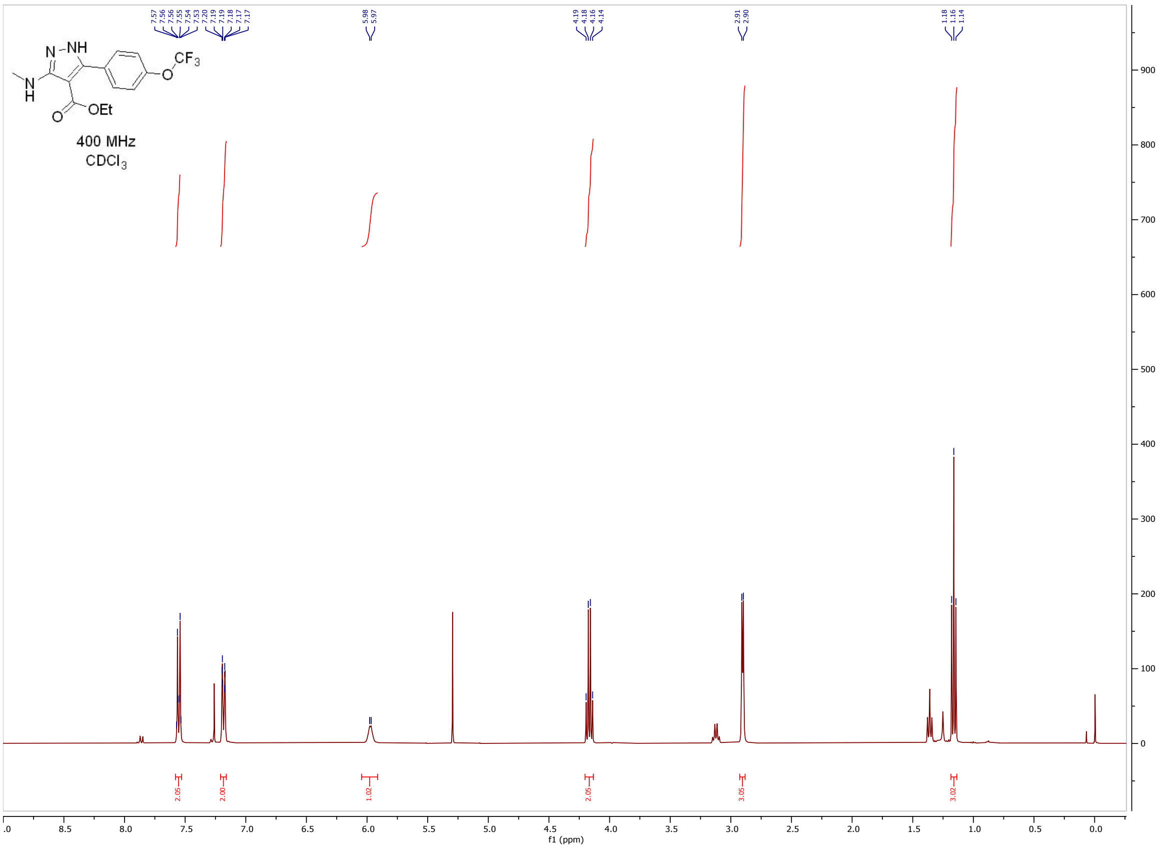

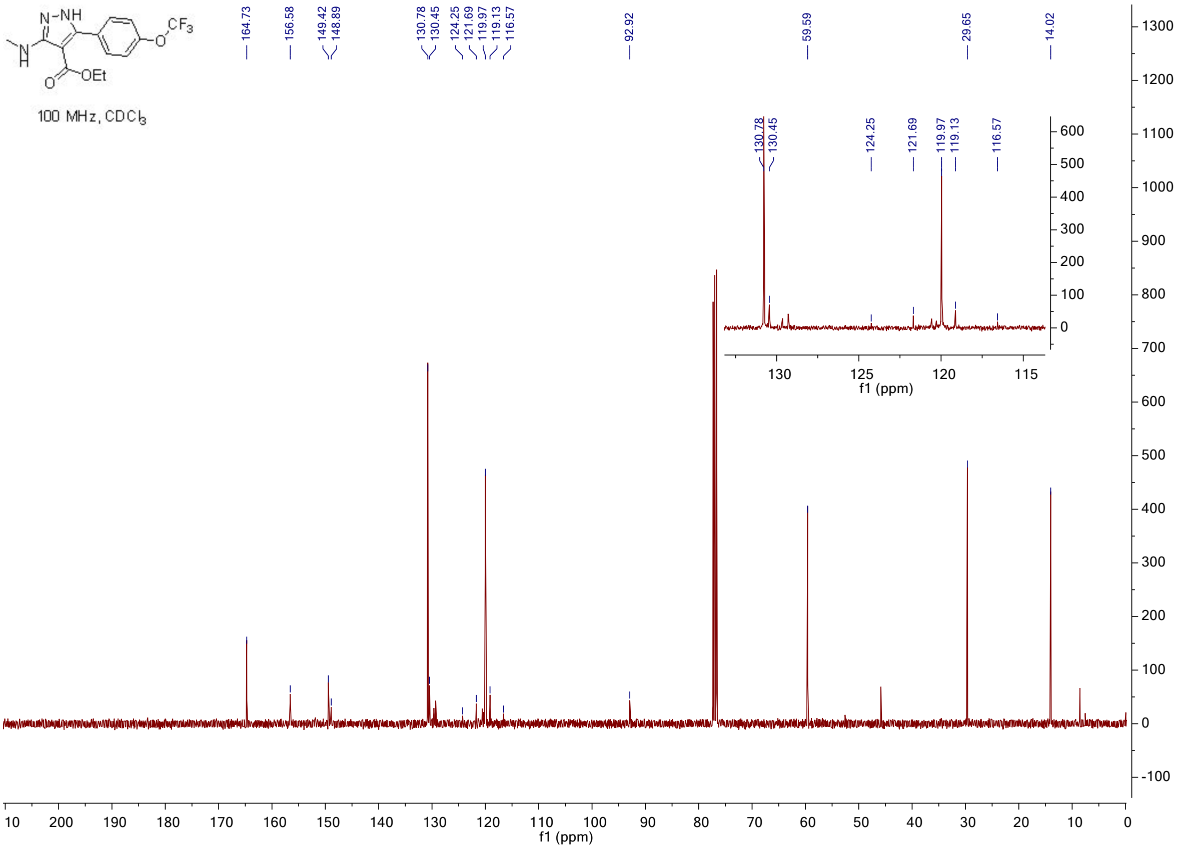

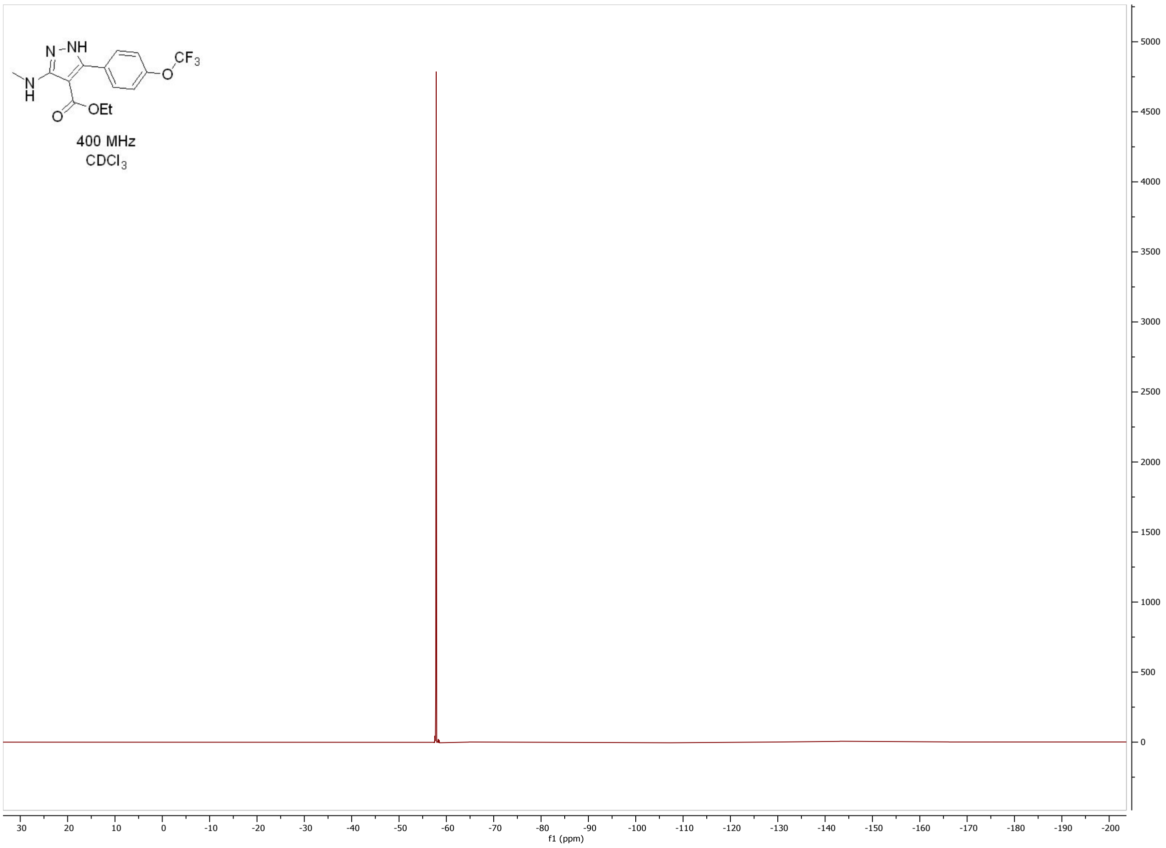

Ethyl 5-(3-chloro-4-methylphenyl)-3-(methylamino)-1*H*-pyrazole-4-carboxylate (4f)

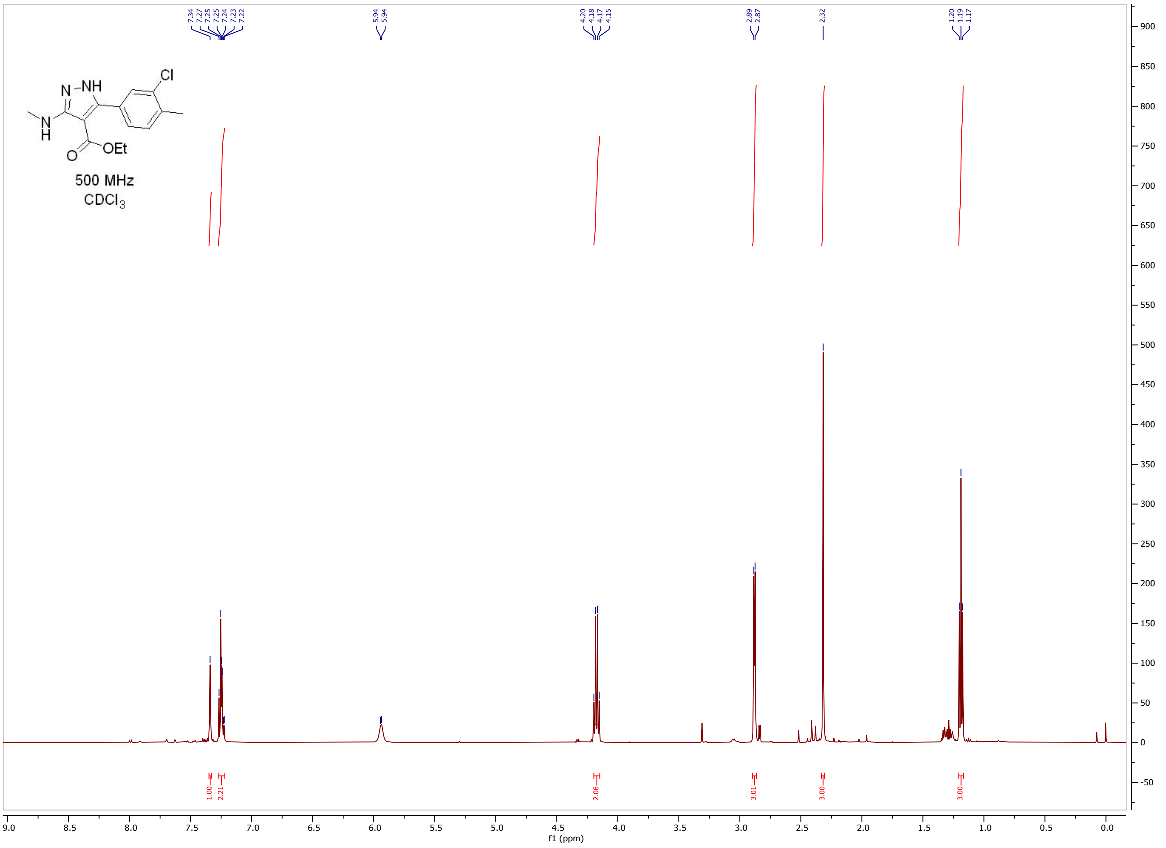

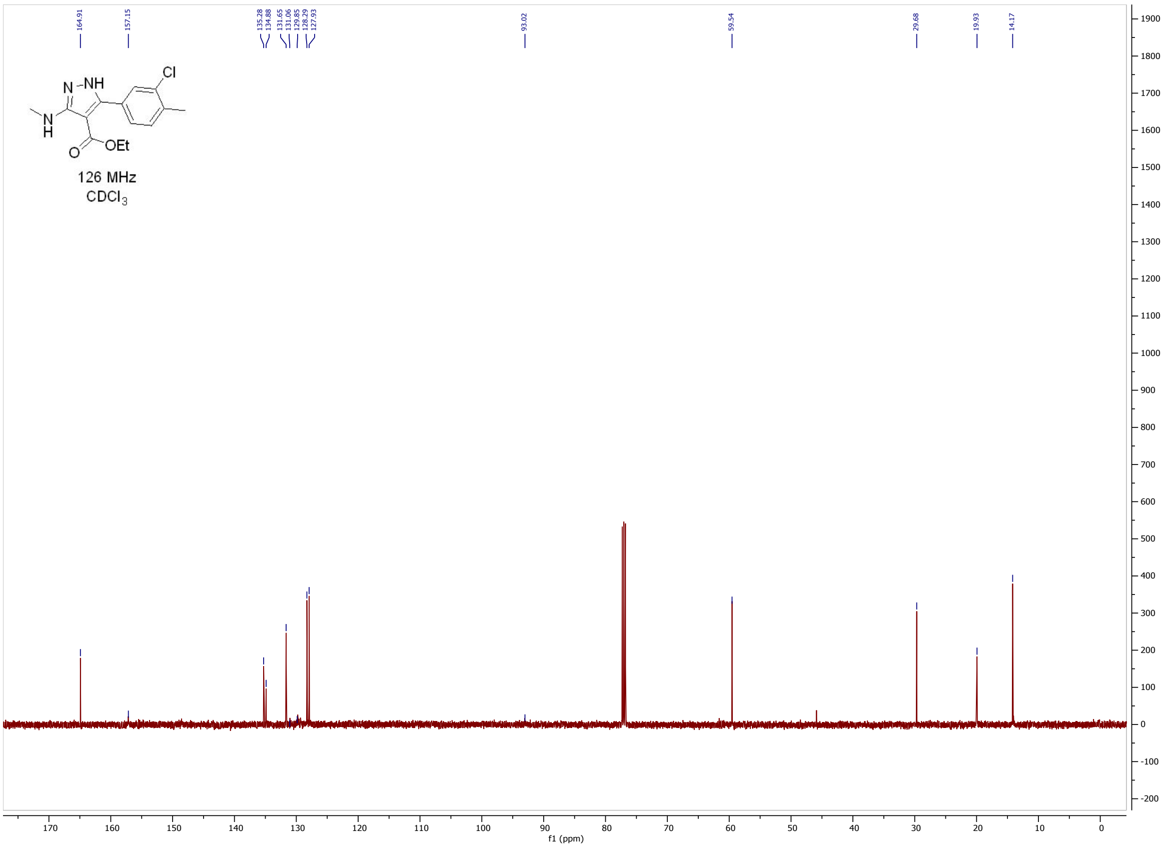
164.91
157.15
135.28
134.88
131.65
131.06
129.85
128.29
127.93
93.02
59.54
29.68
19.93
14.17
Cl
N
NH
N
H
O
OEt
126 MHz
CDCl3
f1 (ppm)

Ethyl 3-(methylamino)-1,5-diphenyl-1*H*-pyrazole-4-carboxylate (5a)

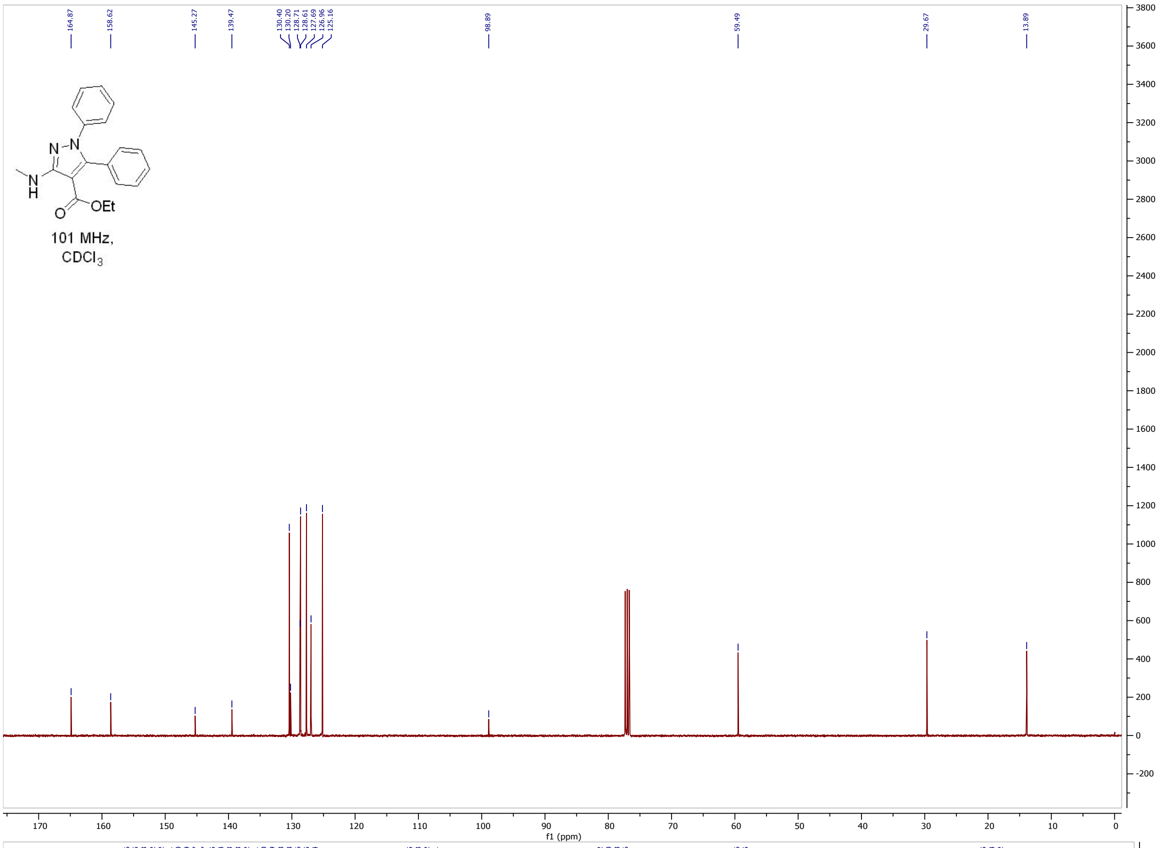

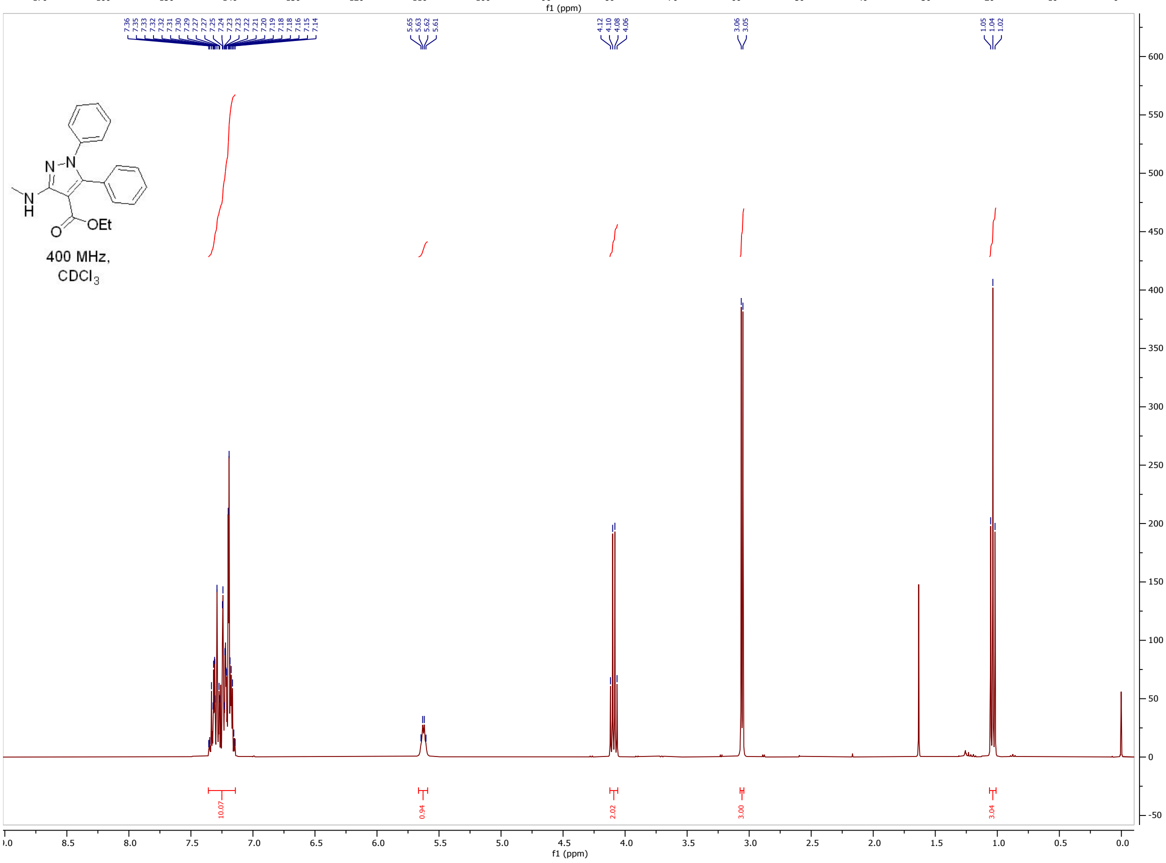

Ethyl 5-(methylamino)-1,3-diphenyl-1*H*-pyrazole-4-carboxylate (5a')

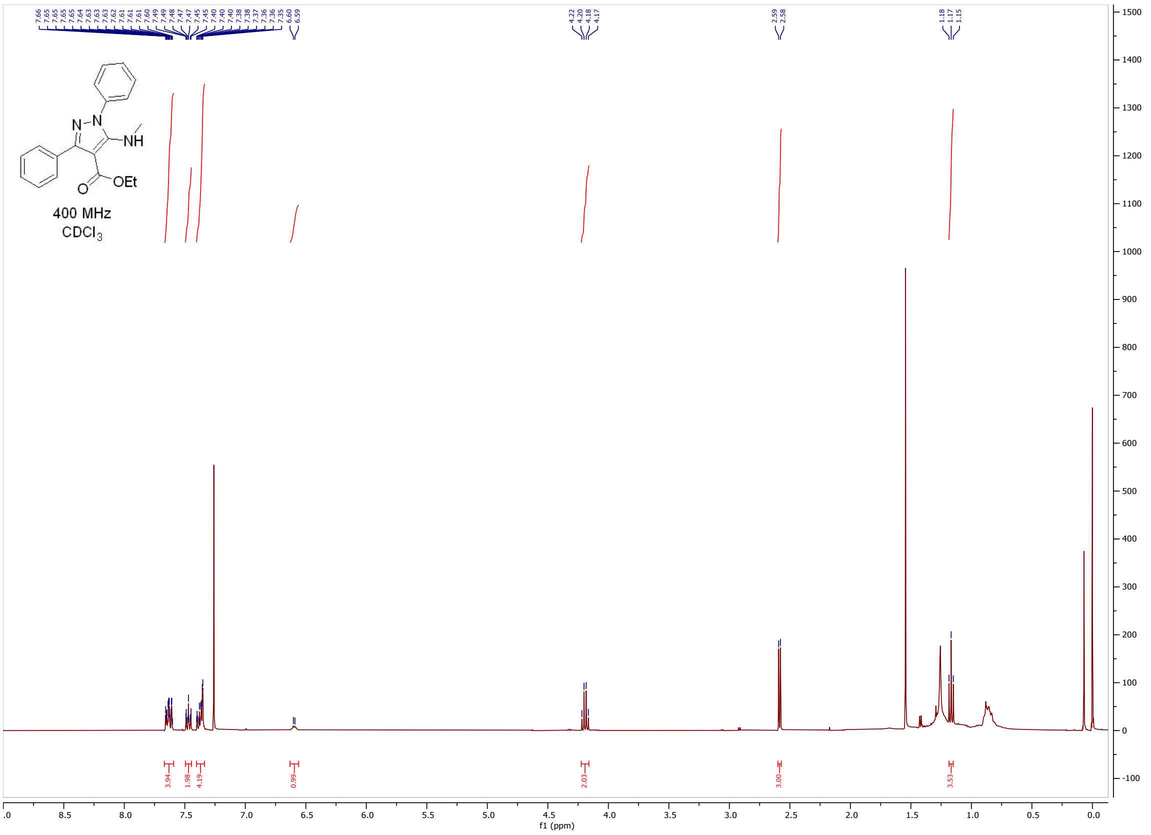

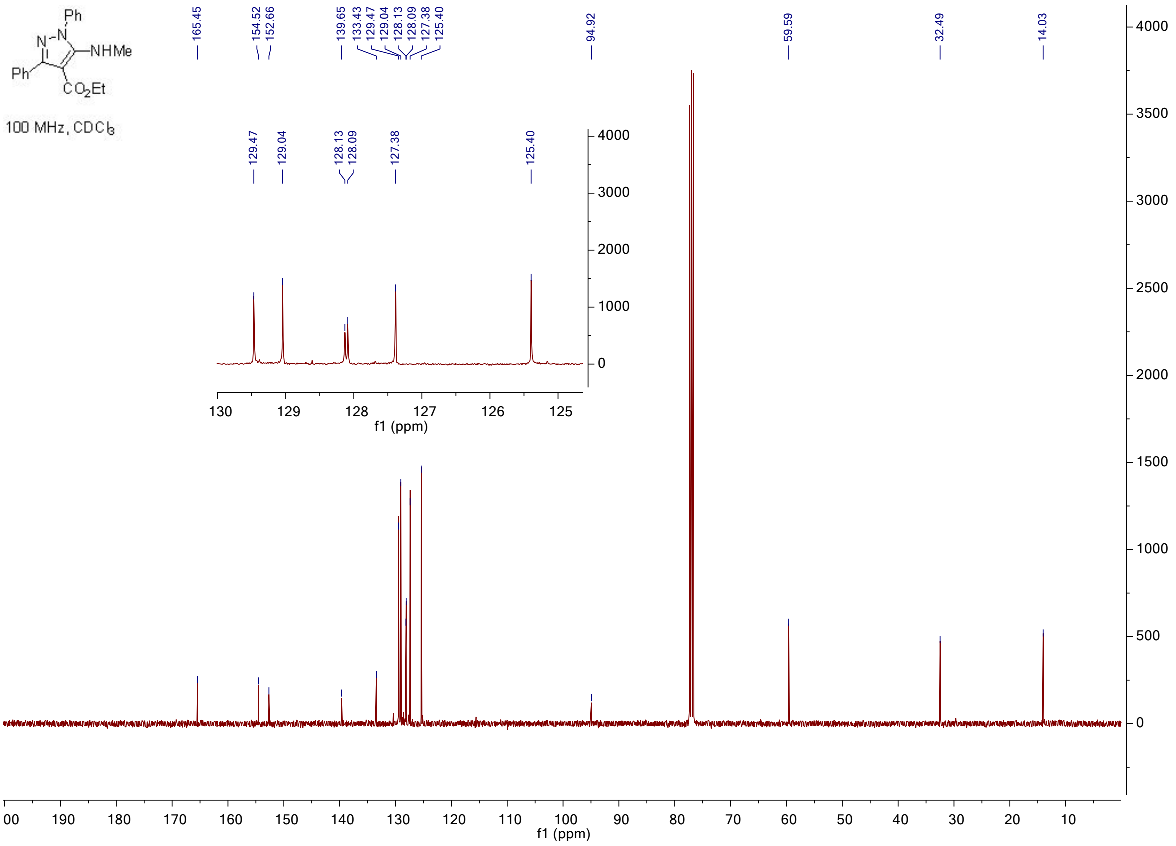

Ethyl 5-(benzo[*d*][1,3]dioxol-5-yl)-3-(methylamino)-1-phenyl-1*H*-pyrazole-4-carboxylate (5b)

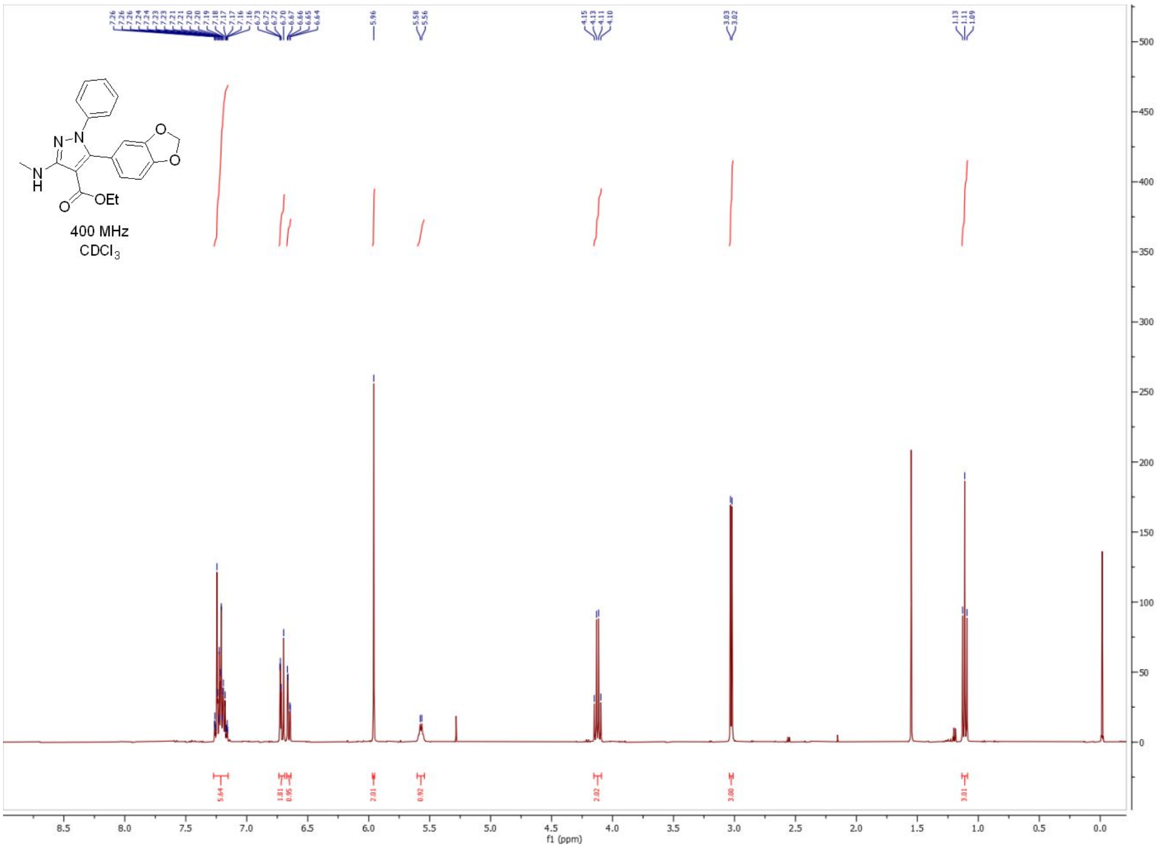

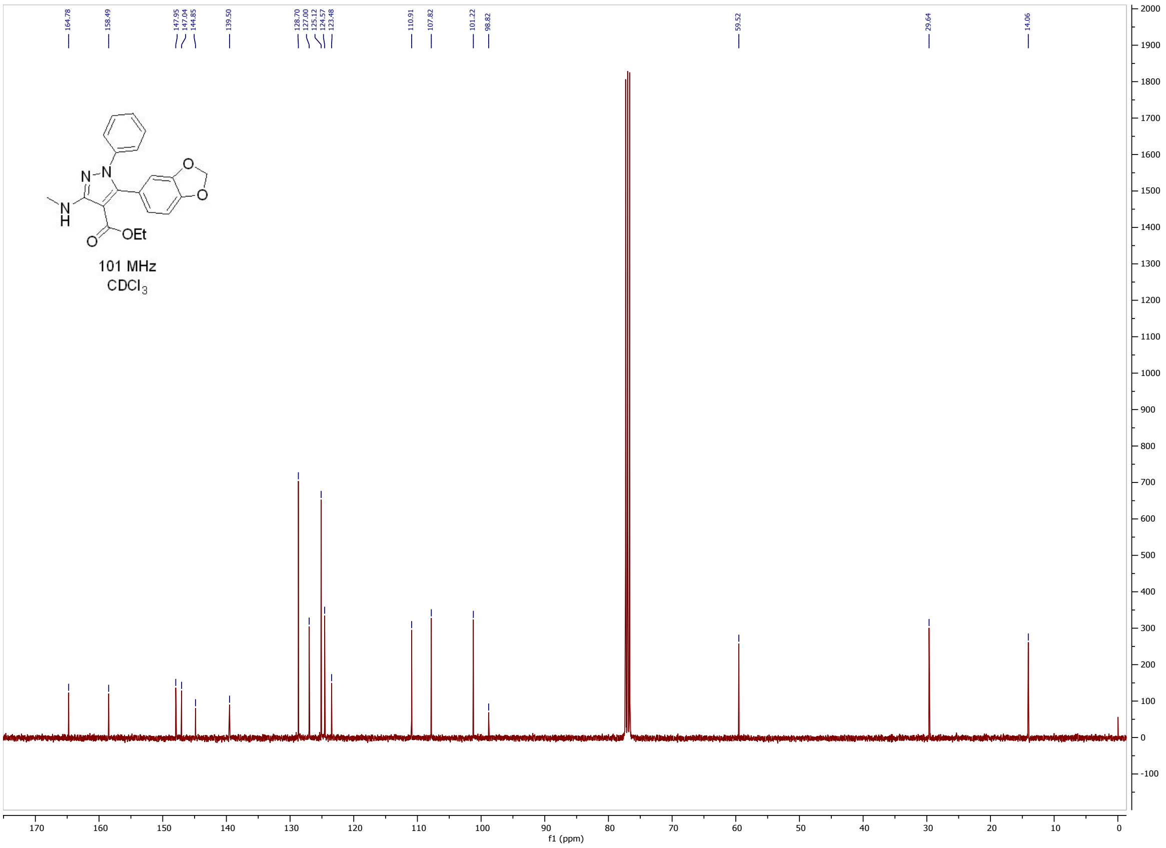

Ethyl 3-(benzo[*d*][1,3]dioxol-5-yl)-5-(methylamino)-1-phenyl-1*H*-pyrazole-4-carboxylate (5b')

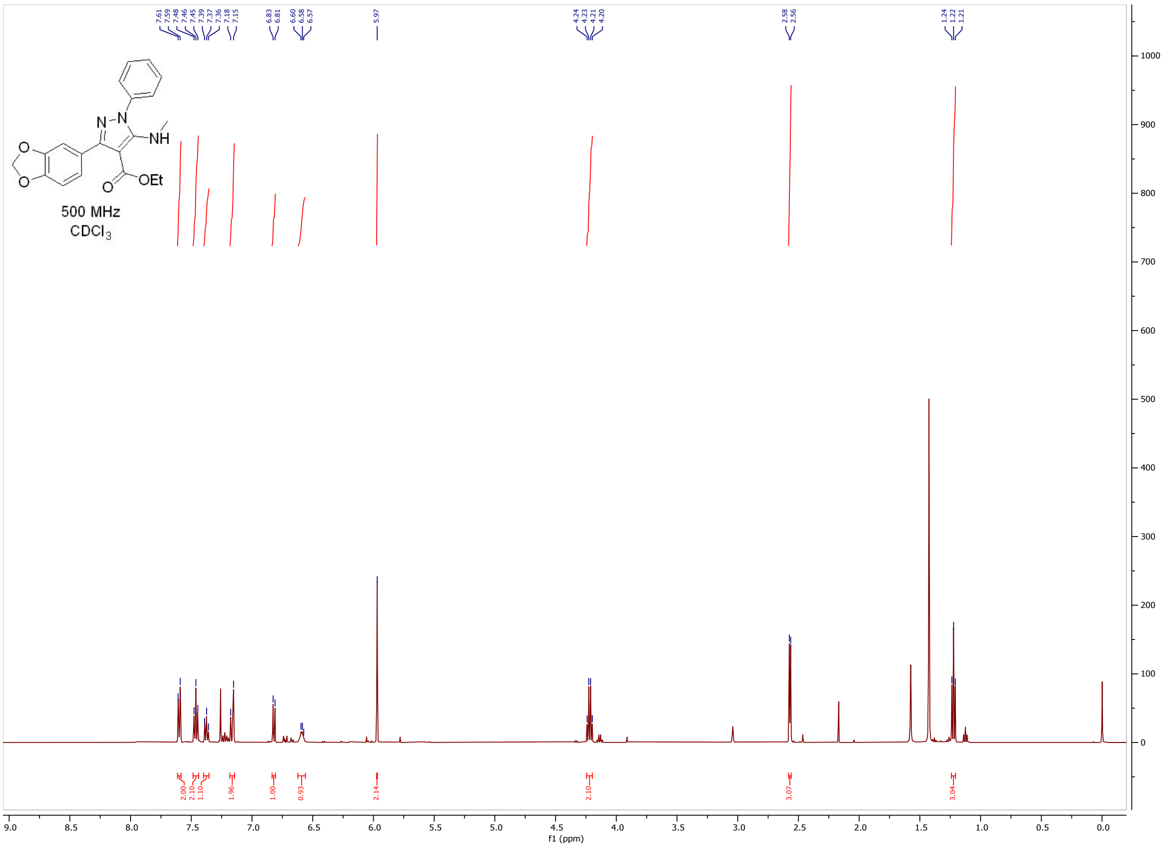

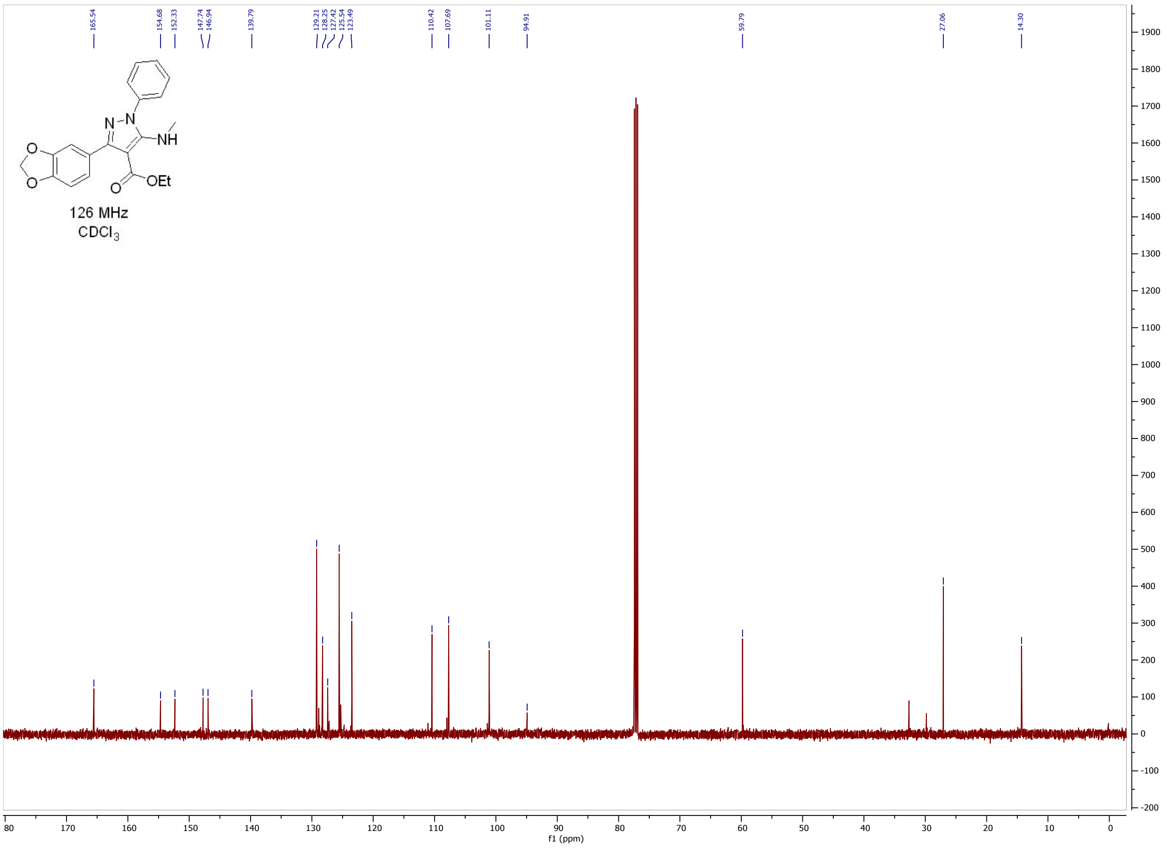

Ethyl 3-(methylamino)-1-phenyl-5-(*p*-tolyl)-1*H*-pyrazole-4-carboxylate (5c)

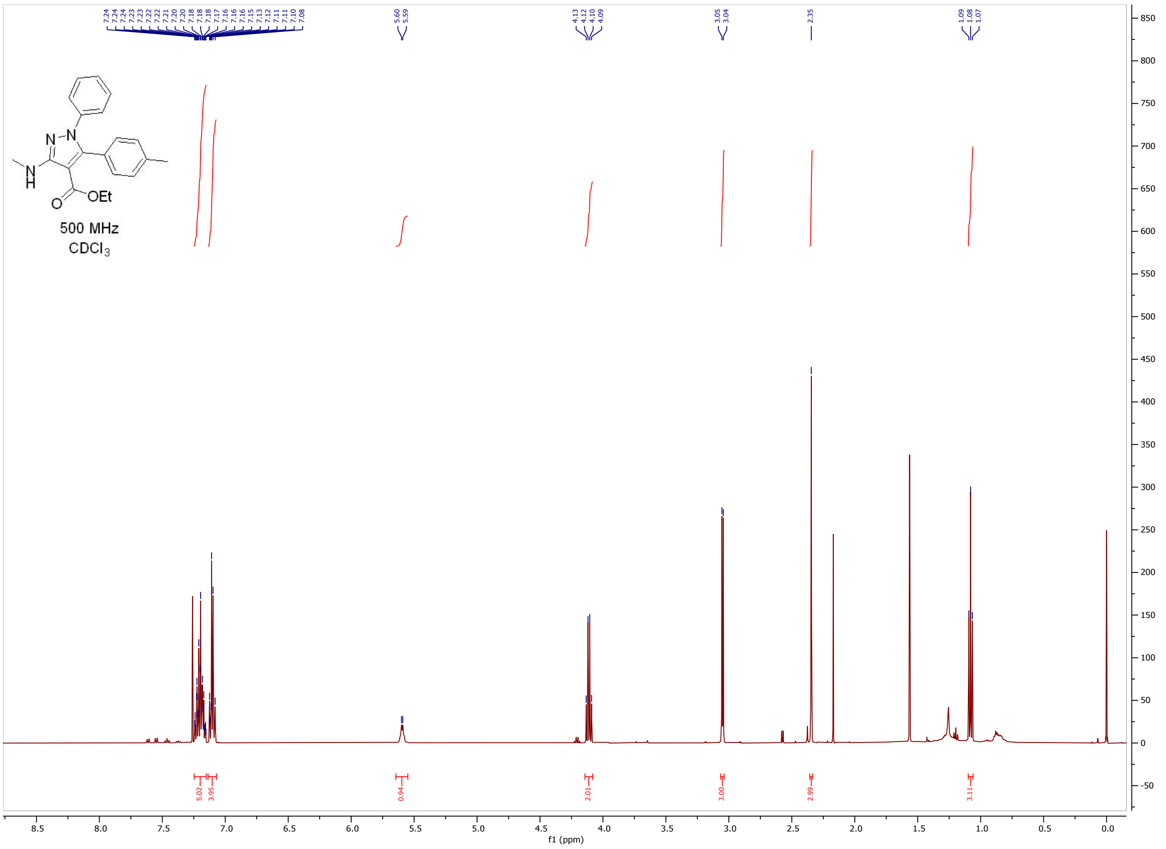

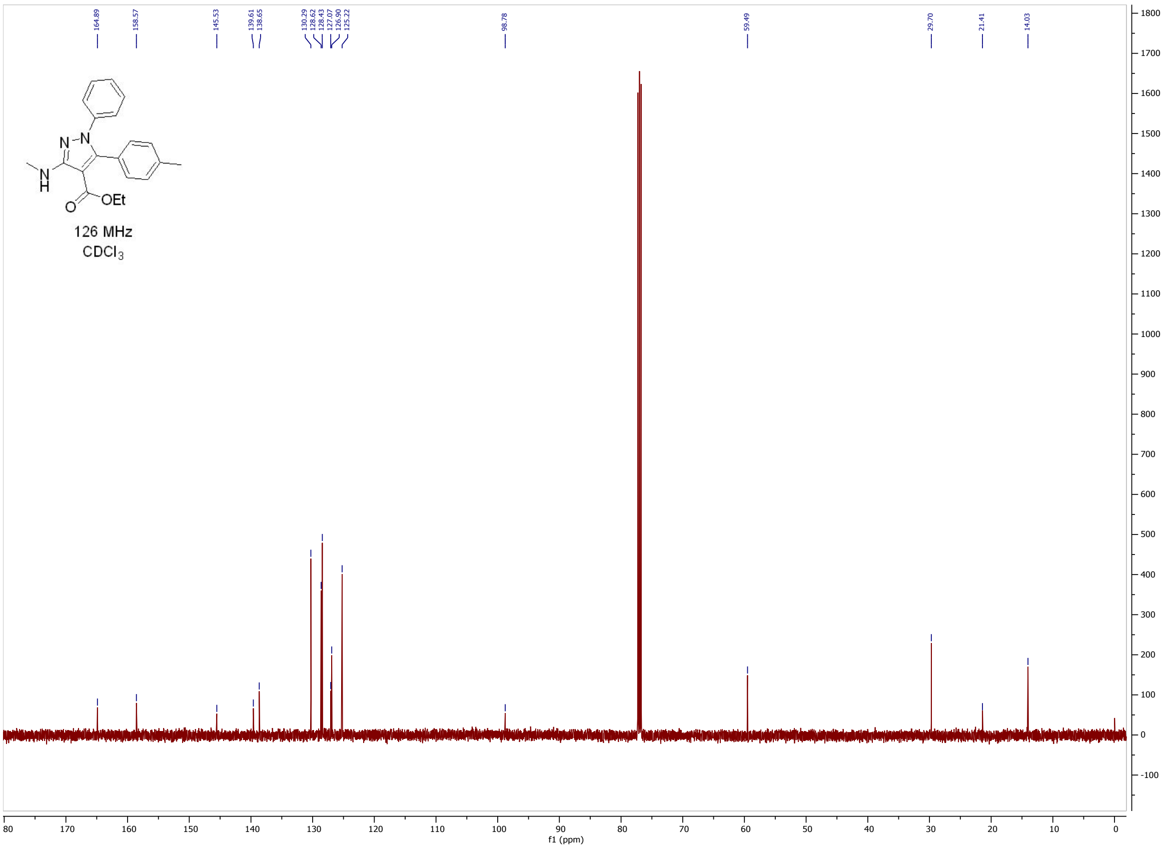

Ethyl 5-(methylamino)-1-phenyl-3-(*p*-tolyl)-1*H*-pyrazole-4-carboxylate (5c')

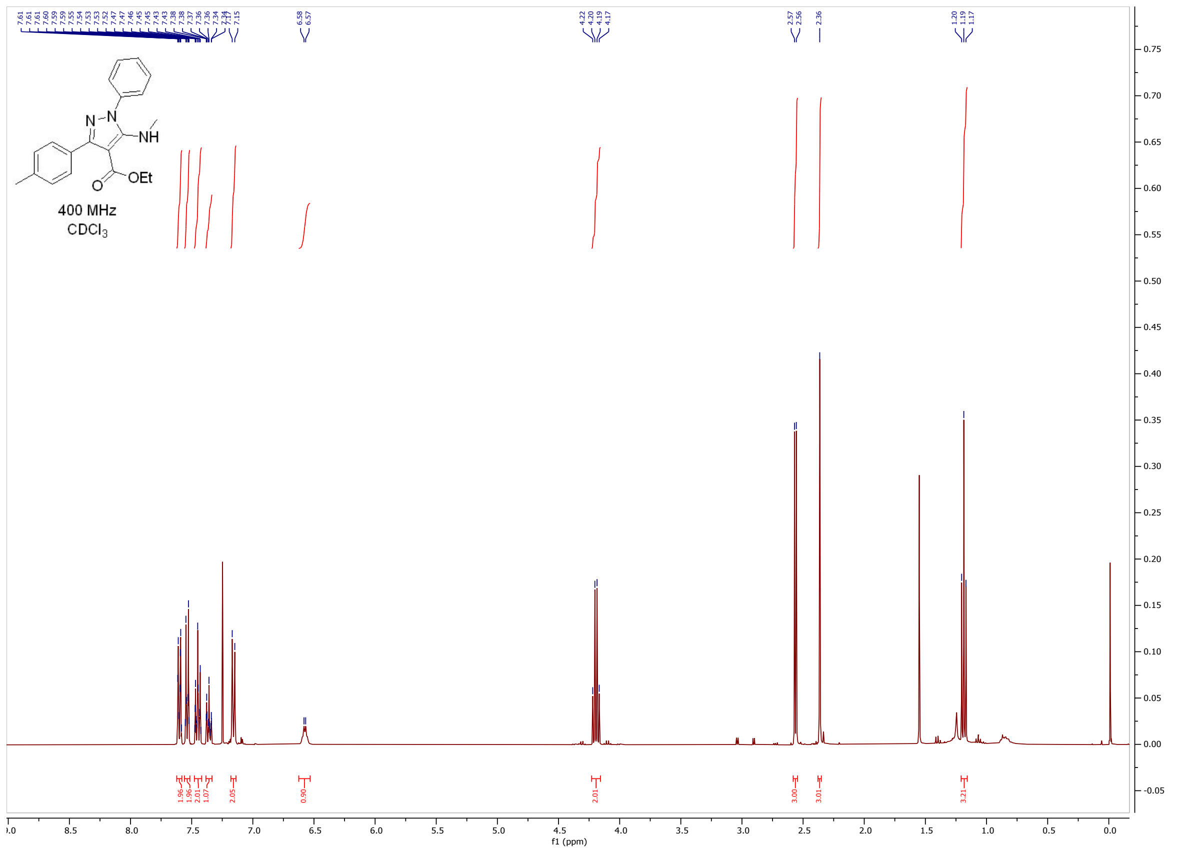

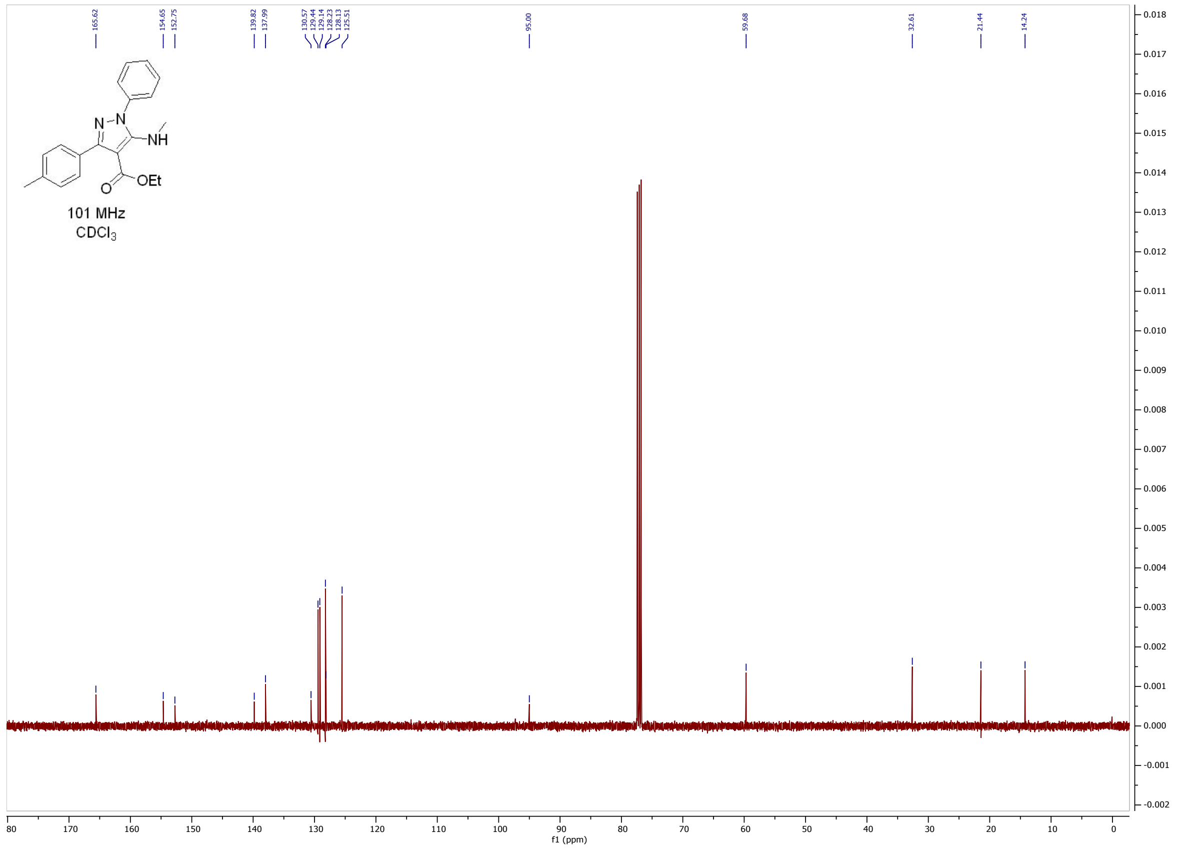

Ethyl 5-(4-bromophenyl)-3-(methylamino)-1-phenyl-1*H*-pyrazole-4-carboxylate (5d)

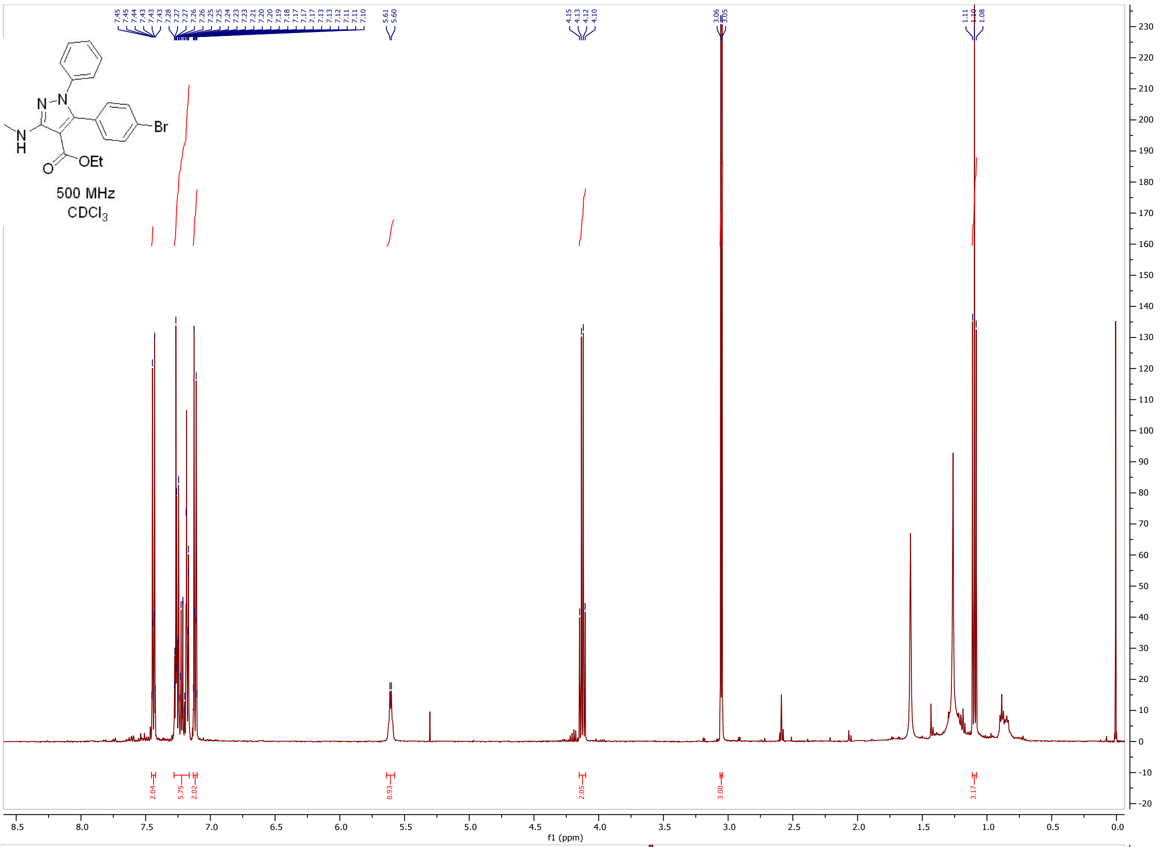

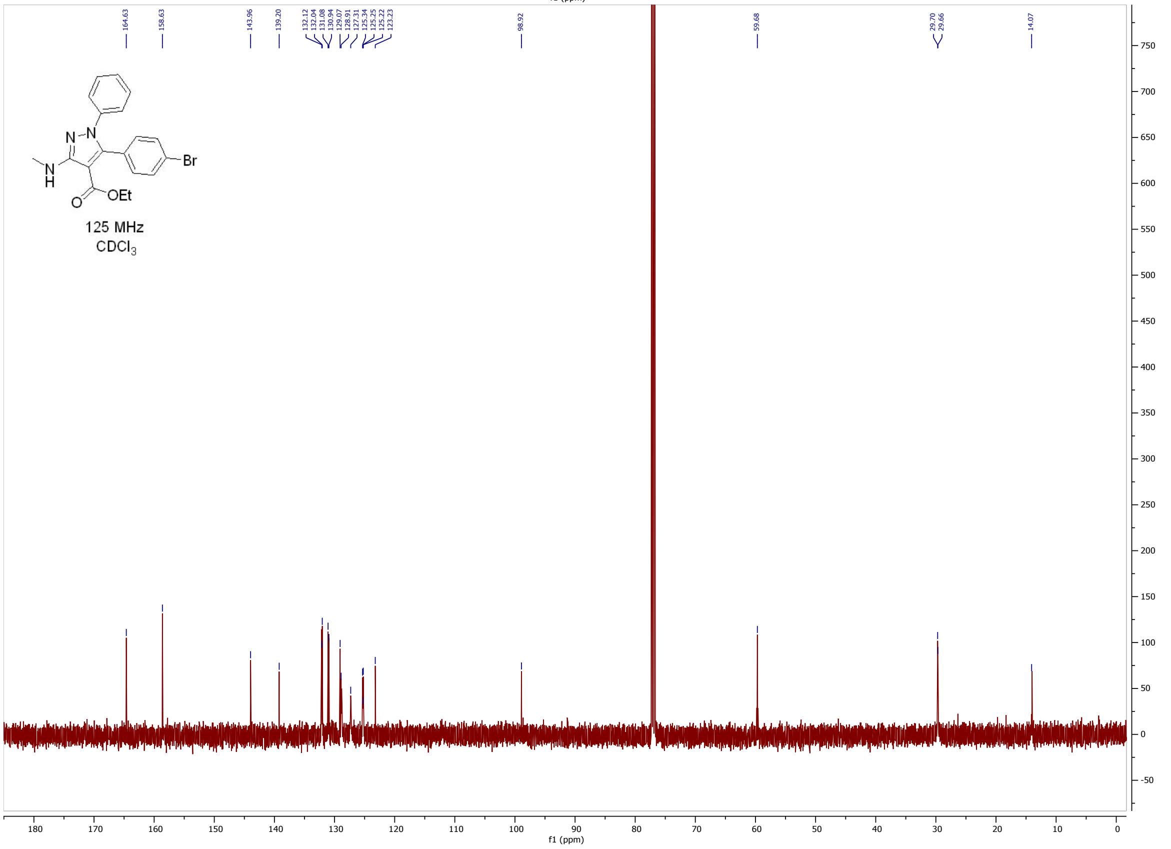

Ethyl 3-(4-bromophenyl)-5-(methylamino)-1-phenyl-1*H*-pyrazole-4-carboxylate (5d’)

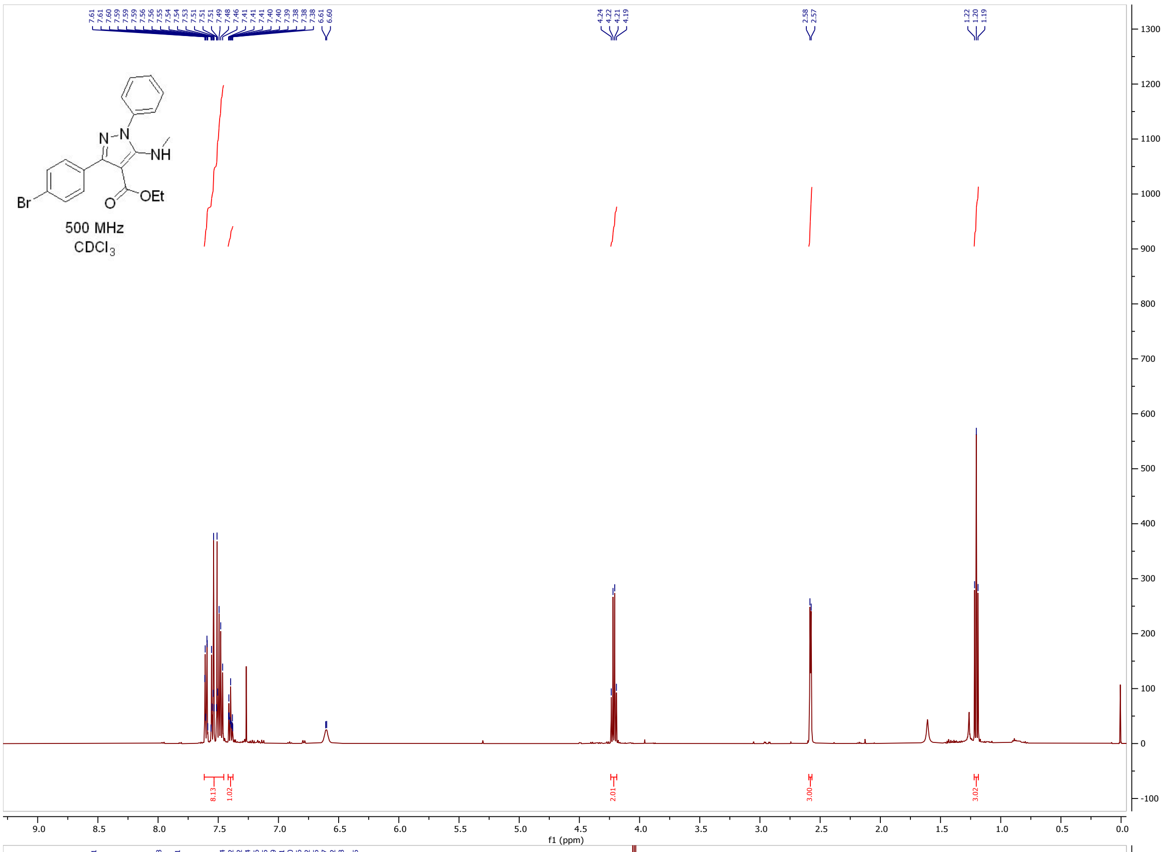
Br
N
N
NH
O
OEt
500 MHz
CDCl3
f1 (ppm)

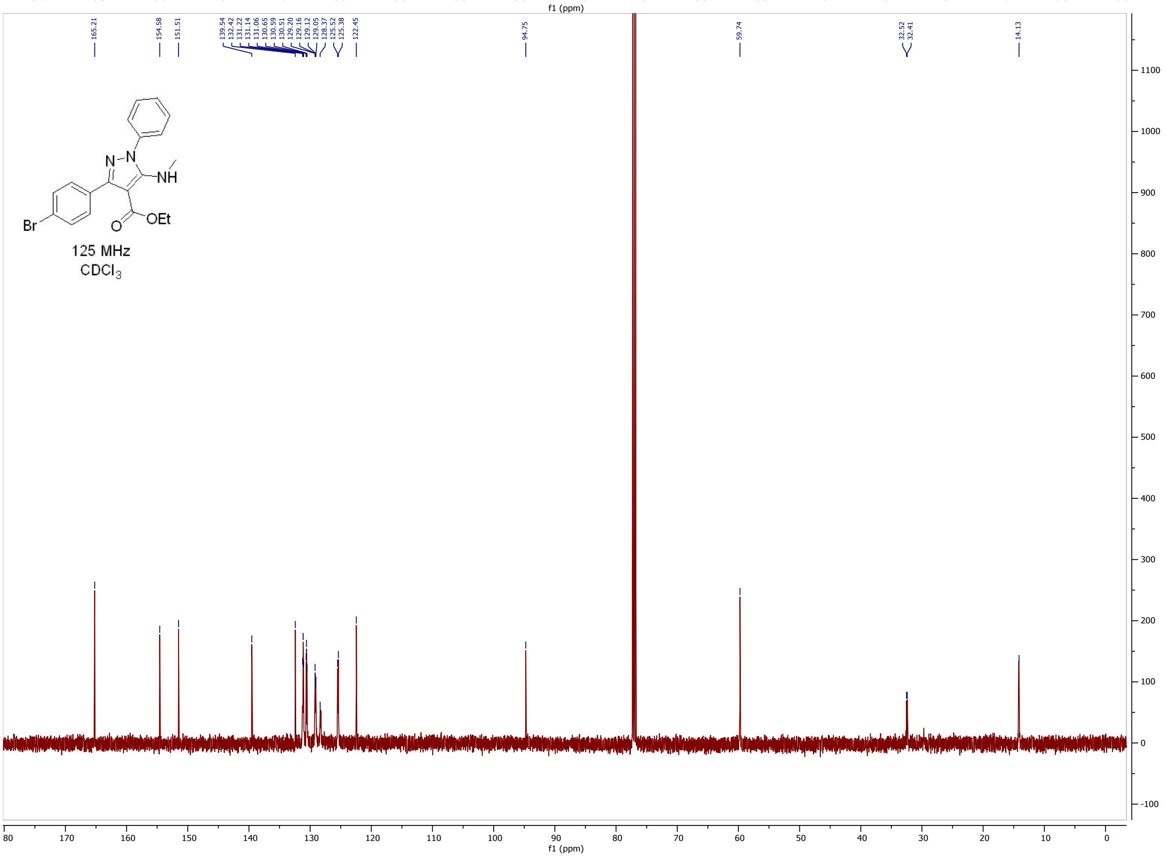
Br
N
N
NH
O
OEt
125 MHz
CDCl3
f1 (ppm)

Ethyl 3-(methylamino)-1-phenyl-5-(4-(trifluoromethyl)phenyl)-1*H*-pyrazole-4-carboxylate (5e)

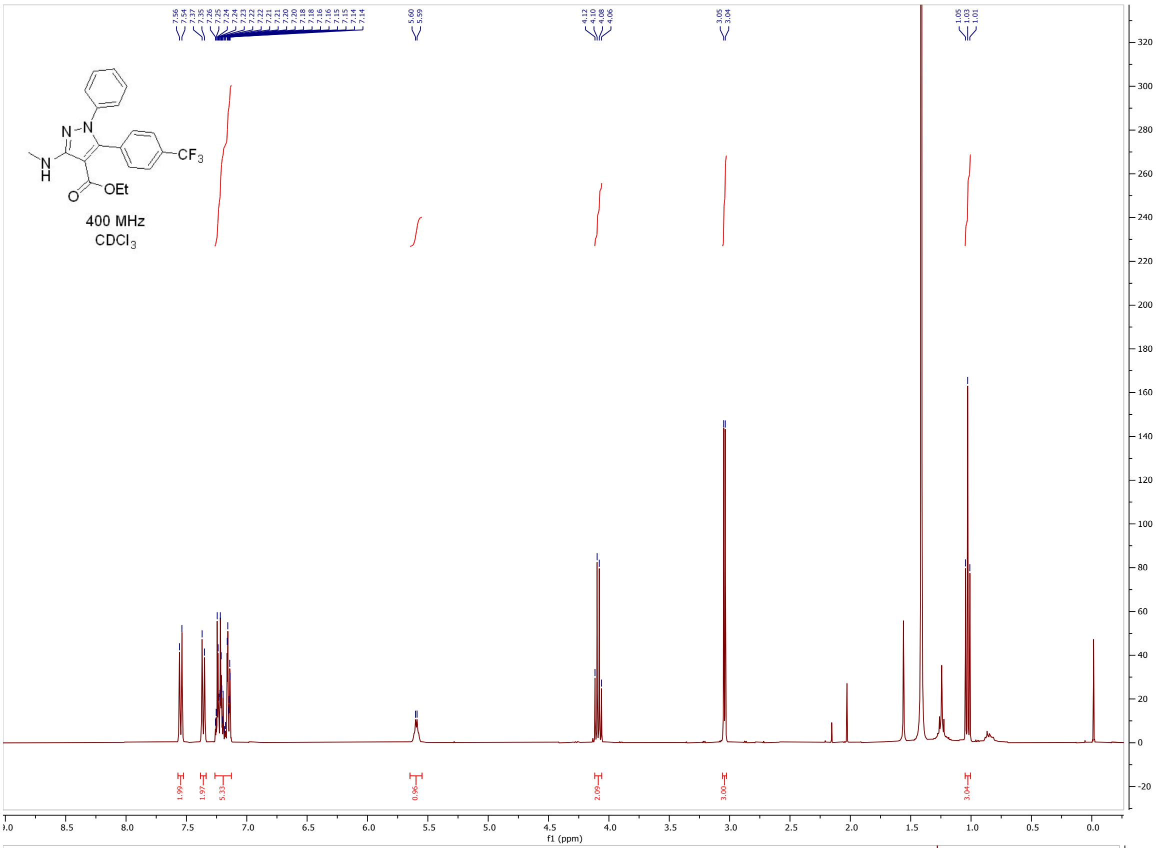

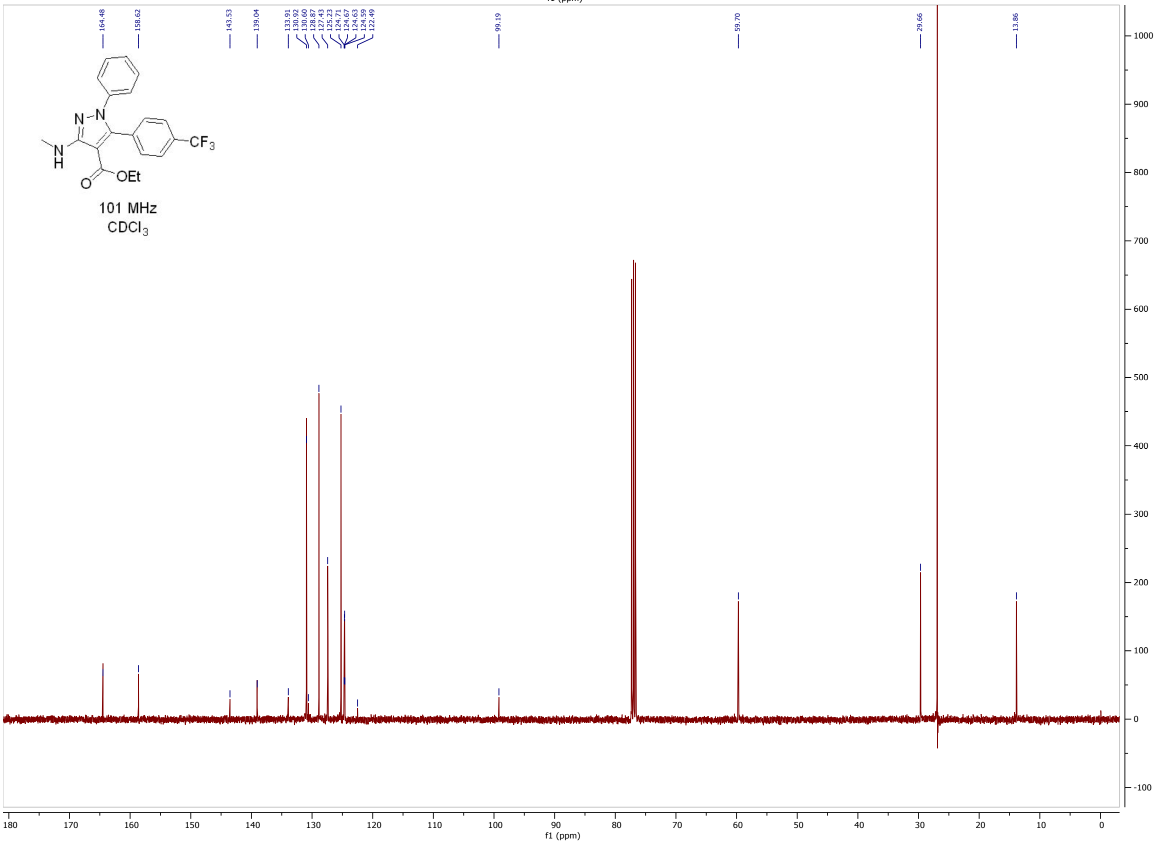

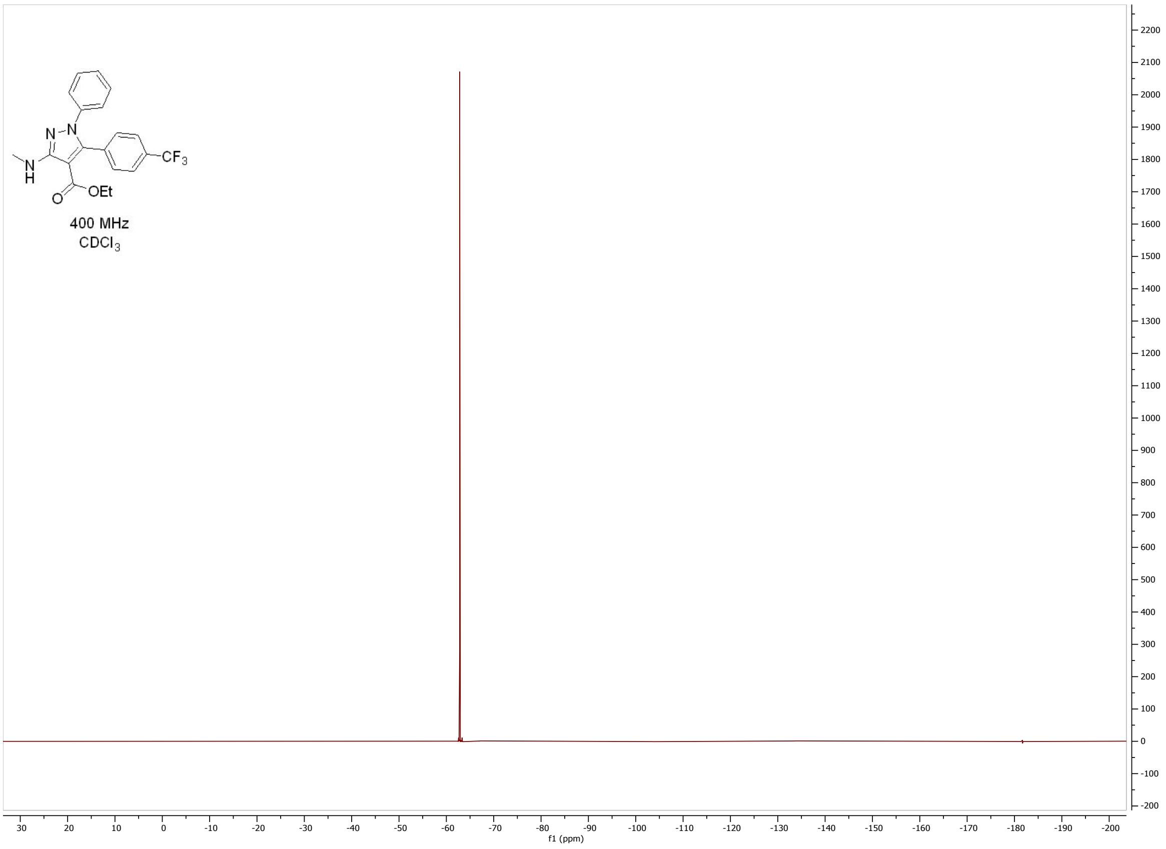

Ethyl 5-(methylamino)-1-phenyl-3-(4-(trifluoromethyl)phenyl)-1*H*-pyrazole-4-carboxylate (5e’)

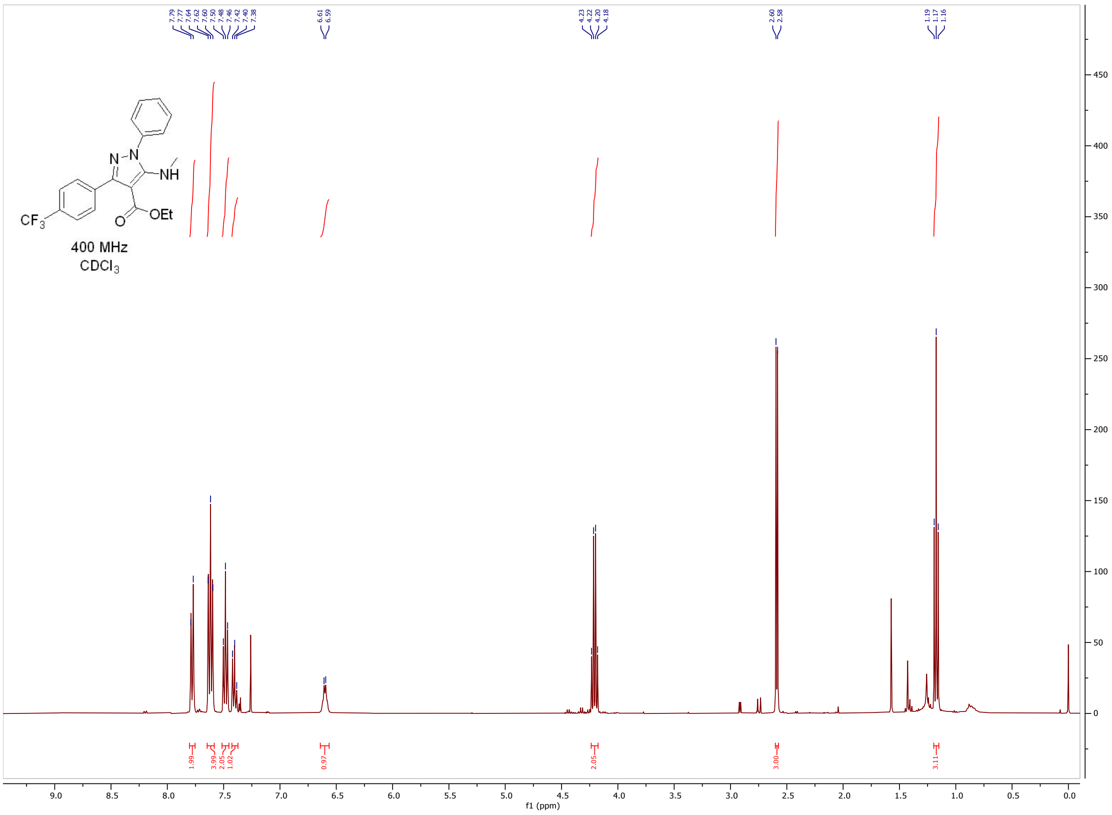

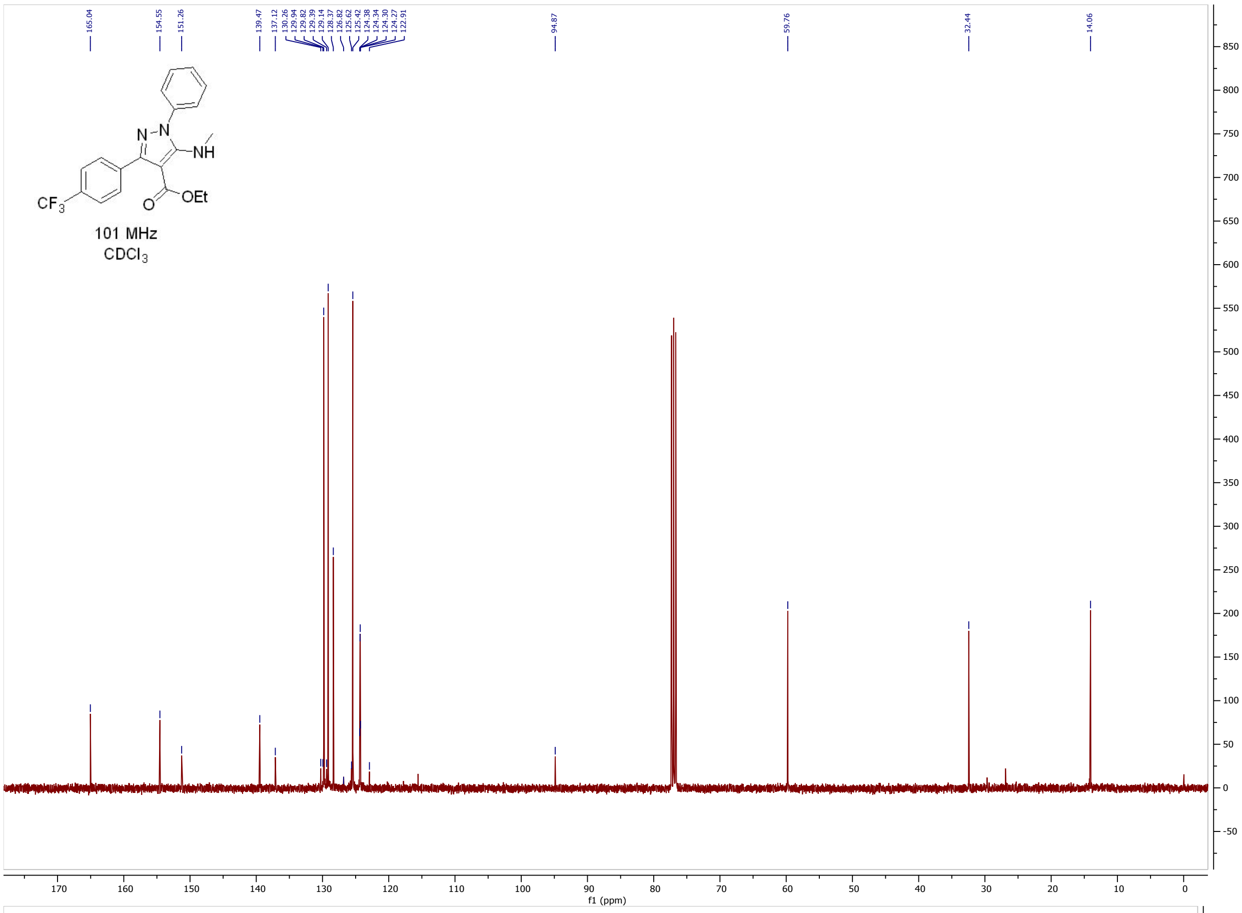
165.04
154.55
151.26
139.47
137.12
130.26
129.94
129.82
129.39
129.14
128.37
126.82
125.62
125.42
124.38
124.34
124.30
124.27
122.91
94.87
59.76
32.44
14.06
N
N
NH
CF3
O
OEt
101 MHz
CDCl3
f1 (ppm)

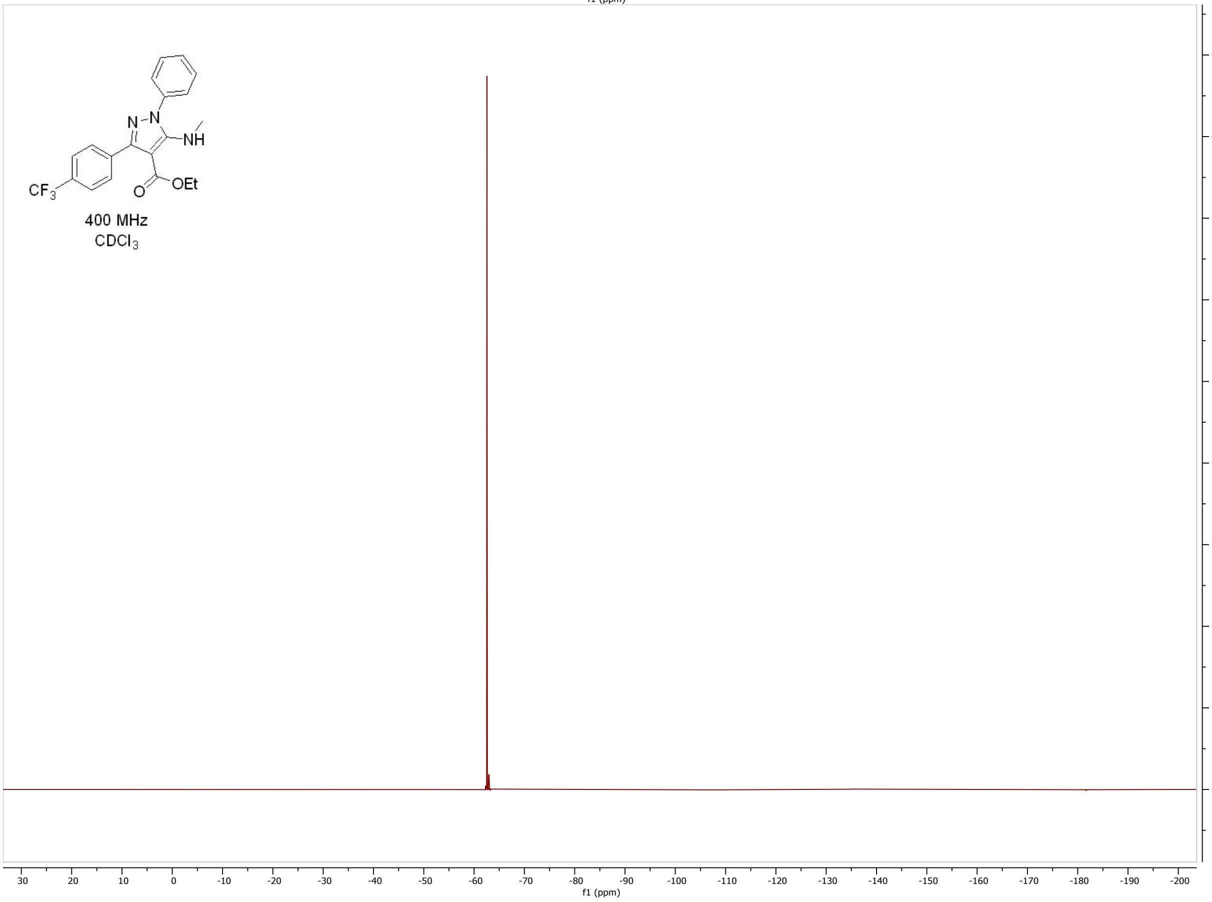
N
N
NH
CF3
O
OEt
400 MHz
CDCl3
f1 (ppm)
